# Genome-wide analyses reveal the detrimental impacts of SARS-CoV-2 viral gene Orf9c on human pluripotent stem cell-derived cardiomyocytes

**DOI:** 10.1016/j.stemcr.2022.01.014

**Published:** 2022-02-17

**Authors:** Juli Liu, Yucheng Zhang, Lei Han, Shuai Guo, Shiyong Wu, Emma Helen Doud, Cheng Wang, Hanying Chen, Michael Rubart-von der Lohe, Jun Wan, Lei Yang

**Affiliations:** 1 Department of Pediatrics, Indiana University School of Medicine, Herman B Wells Center for Pediatric Research, Indianapolis, IN 46202, USA; 2 Department of Medical and Molecular Genetics, Indiana University School of Medicine, Indianapolis, IN 46202, USA; 3 Department of Biochemistry and Molecular Biology, Indiana University School of Medicine, Indianapolis, IN 46202, USA

**Keywords:** SARS-CoV-2, Orf9c, apoptosis, cardiac dysfunction, cardiomyocyte, human pluripotent stem cell, ivermectin, meclizine

## Abstract

Patients with coronavirus disease 2019 (COVID-19) commonly have manifestations of heart disease. Severe acute respiratory syndrome coronavirus 2 (SARS-CoV-2) genome encodes 27 proteins. Currently, SARS-CoV-2 gene-induced abnormalities of human heart muscle cells remain elusive. Here, we comprehensively characterized the detrimental effects of a SARS-CoV-2 gene, Orf9c, on human pluripotent stem cell-derived cardiomyocytes (hPSC-CMs) by preforming multi-omic analyses. Transcriptomic analyses of hPSC-CMs infected by SARS-CoV-2 with Orf9c overexpression (Orf9c^OE^) identified concordantly up-regulated genes enriched into stress-related apoptosis and inflammation signaling pathways, and down-regulated CM functional genes. Proteomic analysis revealed enhanced expressions of apoptotic factors, whereas reduced protein factors for ATP synthesis by Orf9c^OE^. Orf9c^OE^ significantly reduced cellular ATP level, induced apoptosis, and caused electrical dysfunctions of hPSC-CMs. Finally, drugs approved by the U.S. Food and Drug Administration, namely, ivermectin and meclizine, restored ATP levels and ameliorated CM death and functional abnormalities of Orf9c^OE^ hPSC-CMs. Overall, we defined the molecular mechanisms underlying the detrimental impacts of Orf9c on hPSC-CMs and explored potentially therapeutic approaches to ameliorate Orf9c-induced cardiac injury and abnormalities.

## Highlights

• Orf9c overexpression (Orf9c^OE^) exhibits global detrimental effects on hiPSC-CMs
• SARS-CoV-2 viral infection and Orf9c^OE^ concordantly affect transcriptome of hiPSC-CMs
• Orf9c^OE^ reduces ATP level and induces apoptosis and dysfunctions of hiPSC-CMs
• Ivermectin and meclizine mitigate abnormalities of Orf9c^OE^ hiPSC-CMs

## Teaser

In this article, Yang and colleagues uncover the global detrimental effects by Orf9c overexpression (Orf9c^OE^) on the transcriptome, proteome, and interactome of hiPSC-CMs. They show Orf9c^OE^ compromises the cellular ATP level, survival, and electrical functions of hiPSC-CMs, and find two FDA-approved drugs, ivermectin and meclizine, could ameliorate Orf9c^OE^-induced abnormalities of hiPSC-CMs.

## Introduction

Since the end of 2019, severe acute respiratory syndrome coronavirus 2 (SARS-CoV-2) has infected more than 250 million people in the world and caused more than five million deaths world widely and more than 774,000 deaths in the United States. SARS-CoV-2 infects cells via binding angiotensin-converting enzyme 2 (ACE2), as well as transmembrane serine protease 2 (TMPRSS2) (Hoffmann et al., 2020) and TMPRSS4 (Zang et al., 2020). ACE2 is highly expressed in human lung, intestine, and heart (Hikmet et al., 2020), which could potentially account for SARS-CoV-2-induced respiratory symptoms (Zhu et al., 2020) and kidney (Cheng et al., 2020) and cardiovascular diseases (Guo et al., 2020). Although the primary cause of mortality by SARS-CoV-2 infection is respiratory failure, cardiac complications, including myocarditis and arrhythmias, prominently contribute to the overall mortality (Guo et al., 2020; Huang et al., 2020; Shi et al., 2020a). A clinical study reported the elevated serum cardiac troponin T levels consistent with the clinical manifestations of cardiac dysfunction in patients of coronavirus disease 2019 (COVID-19) (Guo et al., 2020). Concomitant cardiovascular disorders have been observed in 8%–25% of the overall SARS-CoV-2 infected population (Dhakal et al., 2020; Klassen et al., 2020; Nishiga et al., 2020). Recent studies found that SARS-CoV-2 could directly infect cardiomyocytes (CMs) of human myocardium (Bailey et al., 2021) and human pluripotent stem cell-derived CMs (hPSC-CMs) (Bojkova et al., 2020; Perez-Bermejo et al., 2020; Sharma et al., 2020). SARS-CoV-2-infected hPSC-CMs show increased cell death (Bojkova et al., 2020; Sharma et al., 2020), fractionated sarcomeres (Perez-Bermejo et al., 2020), and abnormal electrical and mechanical functions, which phenocopy the myocardium injuries of patients with COVID-19 (Yang et al., 2020). Therefore, hPSC-CMs provide an ideal *in vitro* system for modeling and studying the molecular mechanisms by which SARS-CoV-2 genes induced CM injury and dysfunction, which is critical for the management of heart conditions of patients under acute and post-acute SARS-CoV-2 infection.

It is known that the SARS-CoV-2 genome processes 14 open reading frames (ORFs) and encodes 27 proteins. Currently, individual SARS-CoV-2 gene-induced abnormalities of human heart muscle remain elusive. A recent study analyzed the SARS-CoV-2 protein interaction map in human HEK293 cells (Gordon et al., 2020) to reveal targets for drug repurposing and found that Orf9c, which is a non-structural accessory protein encoded by the SARS-CoV-2 genome, could interact with mitochondrial and innate immune pathway proteins (Gordon et al., 2020). Additionally, Orf9c overexpression in human lung epithelial cells could suppress antiviral responses and impair interferon signaling, antigen processing and presentation, complement signaling, and induce IL-6 signaling (Andres et al., 2020), implying that Orf9c could mediate the immune evasion of SARS-CoV-2. To date, the response of human CMs to Orf9c remains unknown. Since mitochondria play a key role in CM function and immune response is critical for SARS-CoV-2-induced inflammation of heart, we decided to define the global impacts of Orf9c on human heart muscle cells by overexpressing Orf9c (Orf9c^OE^) in hPSC-CMs. Genome-wide transcriptomic analysis revealed that both Orf9c^OE^ and SARS-CoV-2 infection of hPSC-CMs activated stress-related cell death, immune and inflammation signaling pathways, and reduced CM functional signaling pathways. Proteomics and interactomic studies uncovered the global impact of Orf9c on proteome of hPSC-CMs and Orf9c-interactive protein network within hPSC-CMs. Orf9c could interact with proteins essential for ATP metabolism and biosynthesis. Further experimental validation confirmed that Orf9c^OE^ induced prominent apoptosis, abnormal calcium handling and electrical properties, and significantly reduced ATP level of hPSC-CMs. Finally, we tested pharmacological approaches to sustain the ATP level of Orf9c^OE^ hPSC-CMs. Notably, two drugs approved by the U.S. Food and Drug Administration (FDA), namely, ivermectin and meclizine, enhanced the ATP level and ameliorated apoptosis and dysfunction of Orf9c^OE^ hPSC-CMs. Overall, we uncovered the global detrimental effects of Orf9c on hPSC-CMs, and explored the alternative usage of FDA-approved drugs for mitigating Orf9c-induced CM injury and dysfunction.

## Results

### Orf9c^OE^ and SARS-CoV-2 infection caused concordant transcriptomic changes of hiPSC-CMs

**Figure 1:**
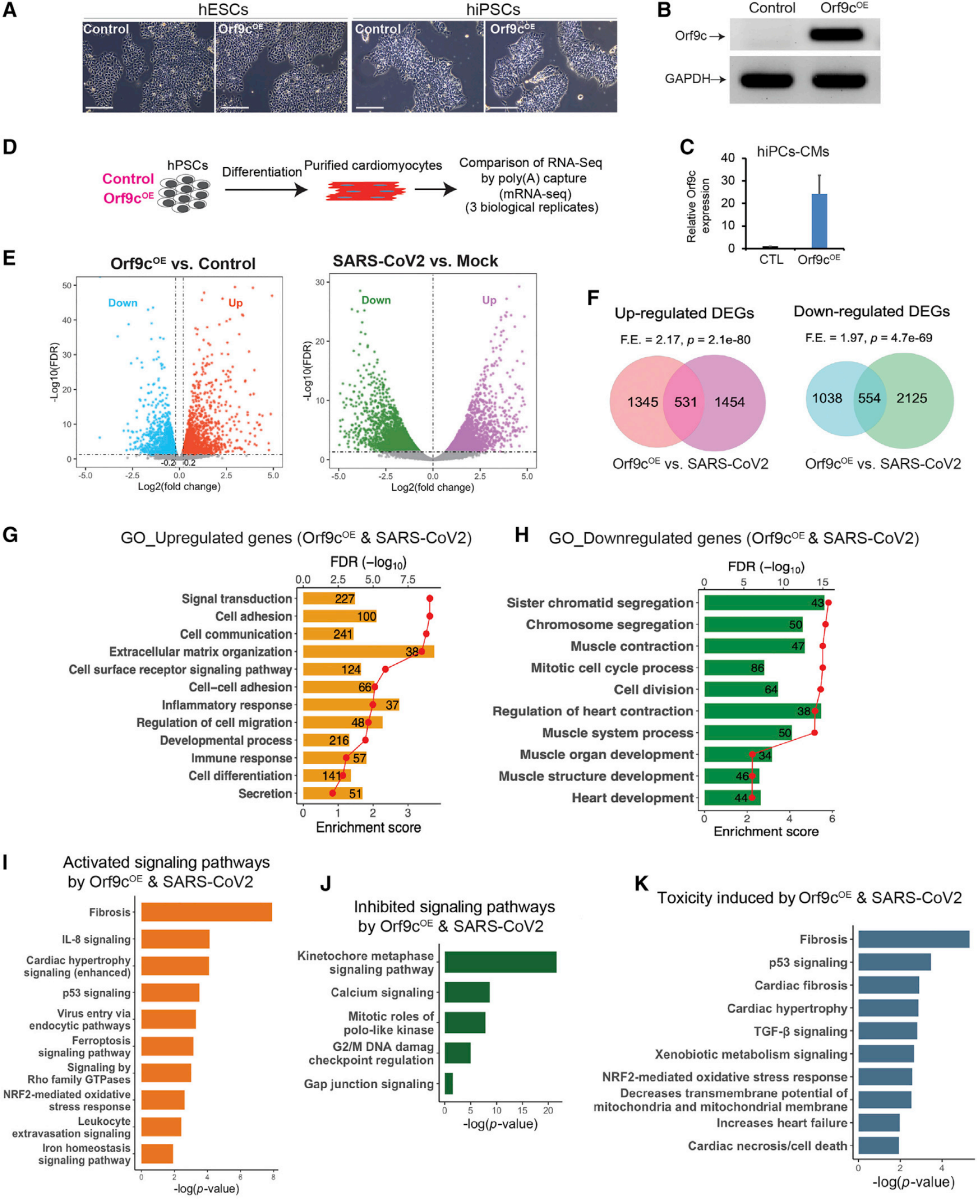
Integrated analyses of mRNA-seq data of hPSC-CMs with Orf9c overexpression and SARS-CoV-2 infection (A) Stable Orf9c^OE^ hESC/hiPSC cell lines cultured with mTesR medium. Scale bar, 200 μM. (B) qRT-PCR detection of Orf9c expression level in undifferentiated control and Orf9c^OE^ hESCs. (C) qRT-PCR detection of Orf9c expression level in control and Orf9c^OE^ hESC-CMs. n = 3. (D) The scheme of mRNA-seq analysis of Orf9c^OE^ and control hESC-CMs. (E) Genes with differential expression levels in hESC-CMs (Orf9c^OE^ vs. controls) and hiPSCs (SARS-CoV-2 vs. mock). (F) Comparison of DE-Gs between hESC-CMs (Orf9c^OE^ vs. Control) and hiPSCs (SARS-CoV-2 vs. Mock). The F.E. around 2 with remarkable *p* value indicates the significant overlap between two gene sets. (G) GO analysis of concordantly up-regulated genes (Orf9c^OE^ and SARS-CoV-2 infection). (H) GO analysis of concordantly down-regulated genes (Orf9c^OE^ and SARS-CoV-2 infection). (I) Canonical signaling pathways activated by both Orf9c^OE^ and SARS-CoV-2. (J) Canonical signaling pathways inhibited by both Orf9c^OE^ and SARS-CoV-2. (K) Cell toxicities induced by both Orf9c^OE^ and SARS-CoV-2.

**Figure 2:**
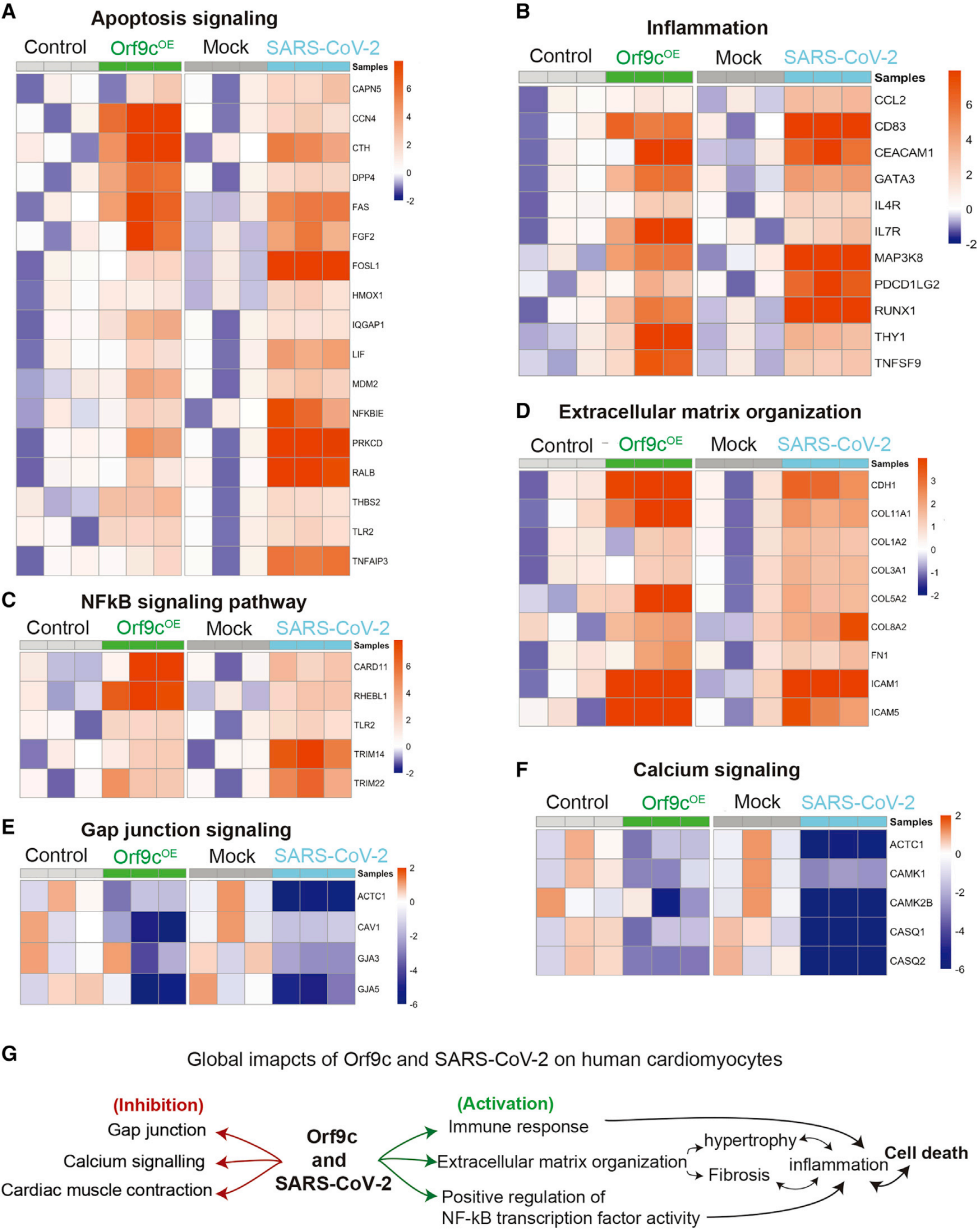
Transcriptomic analyses of the common impacts Orf9c^OE^ and SARS-CoV-2 infection on hPSC-CMs (A) Heatmap showing apoptosis signaling pathway genes activated by both Orf9c^OE^ and SARS-CoV-2 in hPSC-CMs. (B–D) Heatmap showing Orf9c^OE^ and SARS-CoV-2 co-activated stress-related signaling pathway genes in inflammation (B), nuclear factor κB (C) and extracellular matrix organization (D) signaling pathways in hPSC-CMs. (E and F) Orf9c^OE^ and SARS-CoV-2 both reduces expression of genes of gap junction (E) and calcium signaling (F) in hPSC-CMs. (G) Global view of the Orf9c^OE^ and SARS-CoV-2 infection-induced transcriptomic changes in hPSC-CMs from mRNA-seq data.

To study the impact of Orf9c on the transcriptome of human CMs, both Orf9c^OE^ H9 hESCs and S3 hiPSCs (Carvajal-Vergara et al., 2010) were established by using lentivirus (Figures 1A and 1B). The morphology of Orf9c^OE^ hESC/hiPSC colonies was indistinguishable from that of control colonies (Figure 1A). Orf9c^OE^ slightly reduced OCT4 and SOX2, but not NANOG expression of hiPSCs (Figure S1A). Next, both control and Orf9c^OE^ hESCs were differentiated into CMs, followed with CM enrichment by adding 4 mM lactate into the medium for 4 days (Tohyama et al., 2013). Orf9c was highly expressed in Orf9c^OE^ hESCs and Orf9c^OE^ hESC-CMs (Figures 1B and 1C). We monitored the expression of key mesoderm marker genes T and MESP1 at day 3 and cardiac marker genes PDGFRα, ISL1, and NKX2.5 at day 6 of differentiation. Orf9c^OE^ only slightly reduced expression of ISL1, but not the other genes (Figure S1B), indicating that Orf9c^OE^ did not prominently affect CM differentiation. Next, mRNA sequencing (mRNA-seq) was performed with control GFP^OE^ and Orf9c^OE^ hESC-CMs (Figure 1D) to profile transcriptomic changes (Figure 1E, left). A previously published mRNA-seq dataset from SARS-CoV-2-infected hiPSC-CMs was also analyzed in parallel (Sharma et al., 2020) (Figure 1E, right). Interestingly, both Orf9c^OE^ and SARS-CoV-2 could affect the transcriptome of hPSC-CMs (Figures 1E and 1F, Table S1). Orf9c^OE^ and SARS-CoV-2 up-regulated 1,345 and 1,454 genes, respectively, in addition to 531 genes increased by both (Figure 1F, left), indicating a significant overlap with fold enrichment (F.E.) of 2.17 and a p value of 2.1 × 10^−80^ between Orf9c^OE^ and SARS-CoV-2. In the meantime, Orf9c^OE^ and SARS-CoV-2 down-regulated 1,592 and 2,679 genes, of which 554 genes were repressed under both conditions, showing the significant (p = 4.7 × 10^−69^) overlap (F.E. = 1.97) (Figure 1F, right). Gene ontology (GO) analyses of all differentially expressed genes (DE-Gs) found that concordantly up-regulated genes were enriched into biofunctions including signal transduction, cell communication and adhesion, extracellular matrix organization, immune response, and inflammatory response, and so on(Figure 1G), suggesting that Orf9c^OE^ and SARS-CoV-2 could possibly promote cell adhesion via increasing extracellular matrix formation and stimulate inflammation response of host hPSC-CMs. In contrast, the concordantly down-regulated DE-Gs were enriched into muscle contraction, cell division, and muscle and heart development biofunctions (Figure 1H), suggesting that Orf9c^OE^ and SARS-CoV-2 could both compromise CM functions. Furthermore, Orf9c^OE^ and SARS-CoV-2 both activated stress-related signaling pathways including fibrosis, IL-8, p53, Rho, and oxidative stress response (Figure 1I), whereas repressed calcium and gap junction signaling pathways essential for CM function (Figure 1J). Toxicity enrichment analysis found the global detrimental effects of Orf9c and SARS-CoV-2 on hPSC-CMs, focusing on fibrosis, p53, mitochondrial damage, and cardiac cell death (Figure 1K). We then zoomed in the stress-related signaling events. Both Orf9c^OE^ and SARS-CoV-2 infection activated expressions of essential genes involved in apoptosis (Figure 2A), inflammation (Figure 2B), nuclear factor κB (Figure 2C) and extracellular matrix organization (Figure 2D) signaling pathways. Orf9c^OE^ and SARS-CoV-2 infection repressed expressions of genes that are key components of the CM gap junction (Figure 2E) and calcium signaling (Figure 2F). Together, Orf9c^OE^ and SARS-CoV-2 infection both globally influenced the transcriptome of hPSC-CMs by activating immune and inflammation responses and cell death signaling pathway, but inhibiting CM calcium and electrical functions (Figure 2G). These concordance analyses suggest that Orf9c could play a key role in SARS-CoV-2 infection-caused CM injuries. For example, Orf9c^OE^ increased mRNA and protein expression levels of P53 in hESC-CMs (Figures S1C–S1E). However, we also noticed that more than 1,000 DE-Gs were only induced by SARS-CoV-2 (Figure 1F), indicating that SARS-CoV-2 genes other than Orf9c could also affect the transcriptome of host hPSC-CMs by targeting other genes.

### Orf9c globally influences the proteome of hPSC-CMs

**Figure 3:**
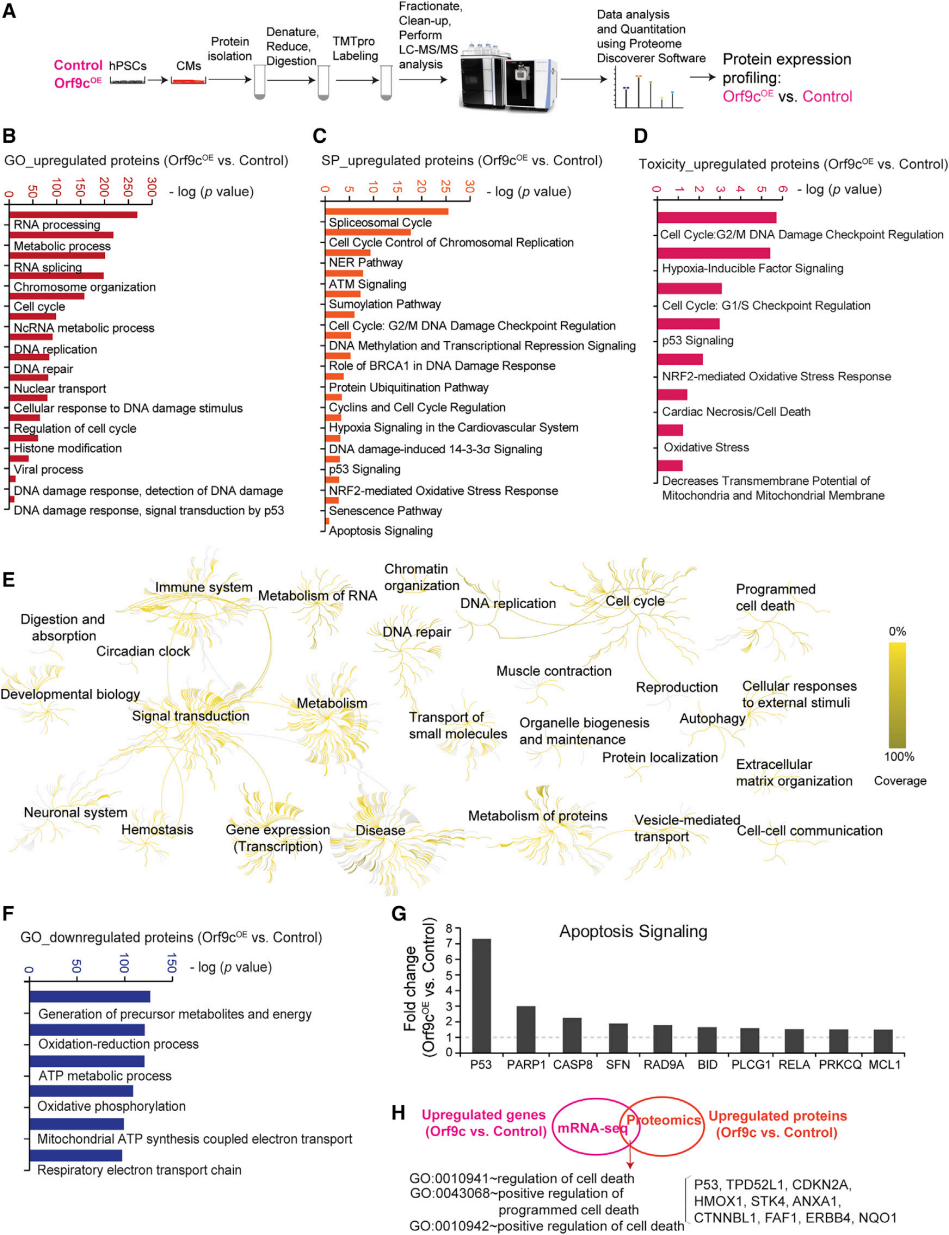
Proteomic analysis of Orf9c^OE^ hESC-CMs (A) The scheme of TMT-MS analysis of Orf9c^OE^ and control hESC-CMs. (B) GO analysis of all up-regulated proteins in Orf9c^OE^ vs. control hESC-CMs. (C) Signaling pathway (SP) analysis of all up-regulated proteins in Orf9c^OE^ vs. control hESC-CMs. (D) Cell toxicity analysis of all up-regulated proteins (Orf9c^OE^ vs. control). (E) Reactome pathway analysis of all up-regulated proteins (Orf9c^OE^ vs. Control). (F) GO analysis of all down-regulated proteins (Orf9c^OE^ vs. Control). (G) Relative expression levels of proteins related to apoptosis signaling pathway. Fold change was calculated by normalizing Orf9c^OE^ to control. Bars represent the average from duplicates. (H) GO analysis of up-regulated genes/proteins identified by overlapping mRNA-seq and proteomic data.

Tandem mass tag-mass spectrometry (TMT-MS) was performed to profile the global protein expression changes in Orf9c^OE^ vs. control hESC-CMs (Figure 3A). A complete list of proteins identified by TMT-MS can be found in Table S2. GO enrichment analysis found that up-regulated proteins (Orf9c^OE^ vs. controls) were over-represented in biofunctions of RNA processing/splicing, metabolism, chromosome/histone organization and DNA damage (Figure 3B), as well as in immune and inflammation responses and cell death (Figures S2A–S2C). Canonical signaling pathway enrichment analysis found that those up-regulated proteins were also involved in spliceosome cycle, cell cycle control, DNA damage regulation, and apoptosis signaling pathways (Figure 3C). Oxidative stress response and cardiac cell death were enriched by up-regulated proteins upon Orf9c^OE^ according to cytotoxicity enrichment analysis (Figure 3D). The reactome pathway analysis provided a global view of key biofunctions that are associated with the up-regulated proteins (Orf9c^OE^ vs. control), including some significant cellular injury events, such as programmed cell death, metabolism, and immune response (Figure 3E). As a comparison, down-regulated proteins (Orf9c^OE^ vs. controls) were enriched into essential cellular energy metabolism events, such as ATP metabolic process and oxidation-reduction process and respiratory electron transport chain (Figures 3F, S2D, and S2E), suggesting that ATP homeostasis of hESC-CMs might be impaired by Orf9c. Notably, TMT-MS data found that the expression levels of many essential apoptotic-related factors were up-regulated in Orf9c^OE^ hESC-CMs compared with control hESC-CMs (Figure 3G). Immunostaining results confirmed the increased protein levels of P53 in Orf9c^OE^ vs. control hESC-CMs (Figures S1D and S1E). Furthermore, by overlapping the Orf9c^OE^-up-regulated genes from mRNA-seq and TMT-MS, we found that the overlapped genes were condensed into cell death events (Figure 3H). Altogether, unbiased transcriptome- and proteome-wide analyses suggest that Orf9c^OE^ could compromise ATP metabolism and induce apoptosis of hPSC-CMs.

### Orf9c overexpression induces apoptosis of hPSC-CMs

**Figure 4:**
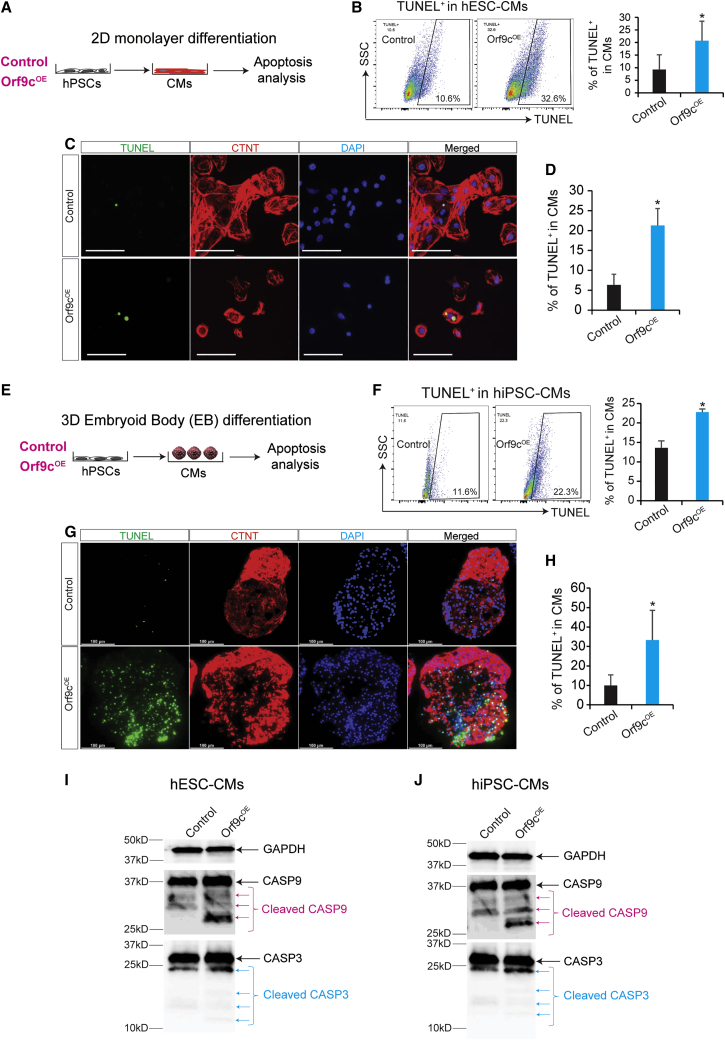
Orf9c^OE^ induces apoptosis of hPSC-CMs (A) HESCs were differentiated into CMs in a 2D monolayer culture, followed by apoptosis analyses. (B) Flow cytometry analysis of the ratios of TUNEL^+^ CMs in hESC-CMs. (C) Representative immunostaining shows TUNEL^+^ and CTNT^+^ CMs in hESC-CMs. Scale bar, 100 μm. (D) Statistical data analysis of immunostaining results of (C). (E) HiPSCs were differentiated into CMs by forming 3D EBs, followed with apoptosis assays. (F) Flow cytometry analysis of TUNEL^+^ CMs in hiPSC-EBs. (G) Representative immunostaining shows TUNEL^+^ CMs in hiPSC-EBs. Scale bar, 100 μm. (H) Statistical analysis of immunostaining results of (G). (I) Western blotting results of CASP3/9, cleaved-CASP3/9 and GAPDH in hESC-CMs. (J) Western blotting results of CASP3/9, cleaved-CASP3/9 and GAPDH in hiPSC-CMs. All bars are shown as mean ± SD (n = 3). A two-tailed unpaired *t* test was used to calculate p values: ^∗^p < 0.05. n = 3.

Orf9c^OE^ and control hESCs were differentiated into CMs using a 2D monolayer differentiation method (Figure 4A) (Lian et al., 2013). After 12 days of differentiation, flow cytometry was conducted to quantify the ratios of TUNEL^+^ cells in CTNT^+^ hESC-CMs. We found significantly increased ratios of TUNEL^+^ CMs in Orf9c^OE^ hESC-CMs when compared with control hESC-CMs (Figure 4B). Immunostaining of the 2D cultured hESC-CMs also observed increased TUNEL^+^ CMs in Orf9c^OE^ vs. control hESC-CMs (Figures 4C and 4D). Next, beating embryoid bodies (EBs) were differentiated from hiPSCs to mimic 3D heart tissues by using our established method (Lin et al., 2012) (Figure 4E). Similar as in 2D culture, prominently increased ratios of TUNEL^+^ CMs were found in Orf9c^OE^ hiPSC-derived EBs when compared with control hiPSC-EBs by using flow cytometry and immunostaining (Figures 4F–4H). To further confirm the results of TUNEL assay, we quantified the ratios of Annexin V^+^/PI^−^ CMs and observed increased ratios of Annexin V^+^/PI^−^ CMs in Orf9c^OE^ hESC-CMs than control hESC-CMs (Figure S3A). Annexin V has a strong affinity for phosphatidylserine residues on the cell surface that is an early marker of apoptosis. We then quantified the ratios of cleaved-CASP3^+^ CMs. Cleaved-CASP3 is an activated form of Caspase 3 responsible for apoptosis execution. Increased percentages of cleaved-CASP3^+^ CMs were found in Orf9c^OE^ hESC-CMs compared with control hESC-CMs, shown by the data from flow cytometry (Figure S3B). Last, the protein levels of apoptotic markers, cleaved-CASP3 and cleaved-CASP9, both increased in Orf9c^OE^ hESC/hiPSC-CMs when compared with control (Figures 4I, 4J, and S3C). Overall, these results demonstrate that Orf9c could induce apoptosis of human heart muscle cells.

### Orf9c interactome in hPSC-CMs

**Figure 5:**
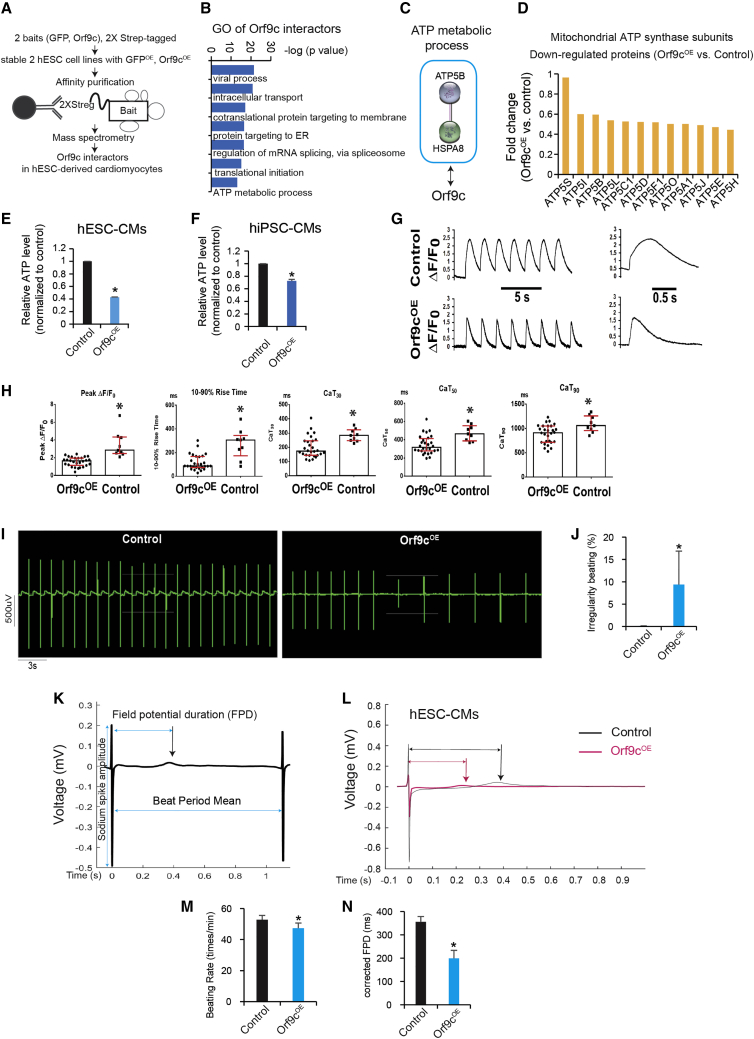
Orf9c^OE^ affects ATP biosynthesis, calcium handing and electrical properties of hPSC-CMs (A) The scheme of co-immunoprecipitation-mass spectrometry (Co-IP MS) to study Orf9c interactors in hESC-CMs. (B) GO analysis of Orf9c interactors in hESC-CMs. (C) Orf9c interaction with ATP metabolic process proteins shown by protein-protein interaction map analysis. (D) Proteomic data of protein expression changes of mitochondrial ATP synthase subunits in Orf9c^OE^ vs. control hESC-CMs. Fold change was calculated by normalizing Orf9c^OE^ to control. (E) Quantification of cellular ATP level of hESC-CMs. All bars are shown as mean ± SD (n = 3). A two-tailed unpaired *t* test was used to calculate p values: ^∗^p < 0.05. (F) Quantification of cellular ATP level of hiPSC-CMs. All bars are shown as mean ± SD (n = 3). A two-tailed unpaired *t* test was used to calculate p values: ^∗^p < 0.05. (G) Representative ΔF/F0(t) traces recorded in a control and an Orf9c^OE^ hESC-CM during 0.5 Hz electrical stimulation. Right panels show the average of three consecutive transients of the same cells on the left. Orf9c^OE^ CM exhibits faster upstroke and decay compared with control CM. The increase in [Ca^2+^]i was biphasic in the control CM, with a fast early and a slow late phase. (H) Summary of [Ca^2+^]i transient properties. Bar height represents median, whereas the whiskers (shown in red) indicate the 25%–75% IQR. Values are from 28 Orf9c^OE^ CMs and nine control CMs. ^∗^p < 0.01 by Mann-Whitney test (peak ΔF/F0, 10–90% rise time, CaT30) or t test (CaT50, CaT90). (I) Representative recordings of spontaneous electrical activities of Orf9c^OE^ and control hESC-CMs by using multi-electrode arrays (MEAs). (J) Statistical results of beating irregularity of MEA data in hESC-CMs. (K) Scheme of electrical properties by MEAs in hPSC-CMs. (L) Electrophysiology analysis of hESC-CMs by MEAs. Representative traces illustrate the change of FPD (ms) between Orf9c^OE^ and control hESC-CMs. (M) Beating rate analysis of MEA data in hESC-CMs. (N) The FPD_c_ of MEA data in hESC-CMs. In (J, M, and N), all bars are shown as mean ± SD (n = 4). A two-tailed unpaired *t* test was used to calculate p values. ^∗^*p* < 0.05. All bars are shown as mean ± SD.

In the Orf9c^OE^ and control GFP hPSCs, Orf9c and GFP were each fused with a Strep-tag (Gordon et al., 2020), which allowed us to pull down Orf9c interactive proteins. Therefore, affinity purification was performed in Orf9c^OE^ and control hESC-CMs by using an anti-Strep antibody, followed with mass spectrometry (Co-IP-MS) to capture and identify all Orf9c interacting proteins (Figure 5A). A complete list of Orf9c interactors can be found in Table S3. GO enrichment analysis revealed that Orf9c interactors were involved in important cellular events, including viral process, protein transport/targeting, mRNA splicing, and ATP metabolic process (Figures 5B and S4A). Notably, we conducted protein-protein interaction map analysis as previously described (Gordon et al., 2020) and found that Orf9c interacted with essential protein factors of ATP metabolism and biosynthesis (Figure 5C). Additionally, from the proteomic data, we found that Orf9c^OE^ reduced protein levels of several ATPase subunits in hESC-CMs (Figures 5D and S2D). These results implied that Orf9c could possibly interfere with ATP biosynthesis of hPSC-CMs. Therefore, we quantified the cellular ATP level of hPSC-CMs and found Orf9c^OE^ significantly reduced the ATP level of hESC-CMs/hiPSC-CMs when compared with control CMs (Figures 5E and 5F). These data confirmed that Orf9c^OE^ impaired ATP biosynthesis of hPSC-CMs. Since compromised ATP biosynthesis could lead to cell death (Eguchi et al., 1997; Izyumov et al., 2004; Shiraishi et al., 2001; Skulachev, 2006; Tatsumi et al., 2003), these data also suggested that the enhanced apoptosis of Orf9c^OE^ hESC-CMs could possibly be due to the reduced ATP level.

### Orf9c compromises calcium handling and function of hPSC-CMs

Cellular ATP level is critical for the electrophysiology of heart muscle in that insufficient ATP level could impair intracellular Ca^2+^ signaling and excitation-contraction coupling of CMs, which result in reduced contractile capacity (Bers, 2008; Bers and Guo, 2005; Fearnley et al., 2011). Accordingly, we recorded electrically evoked [Ca^2+^]_i_ transients in fluo-4-loaded Orf9c^OE^ hESC-CMs using confocal fluorescence microscopy. Representative *Δ*F/F0 transients recorded from a control and an Orf9-expressing hiPSC-CM during 0.5 Hz electrical field stimulation are shown in Figure 5G. Peak *Δ*F/F0 elevation was smaller and *Δ*F/F0 transient duration was shorter in the Orf9c^OE^ CMs. Diastolic *Δ*F/F0 returned to pre-stimulation, i.e., resting, levels in the Orf9c^OE^ CMs, but remained elevated in the control CMs. Plots of temporally averaged transients on an expanded timescale (right panels in Figure 5G) revealed distinct differences in *Δ*F/F0 amplitude and kinetics. The *Δ*F/F0 rise was biphasic in the control CM, with a fast early and a slow late phase, whereas it was monophasic and peaked earlier in the Orf9c^OE^ CM. The *Δ*F/F0 recovery was faster and the overall duration of the *Δ*F/F0 transient was shorter in the Orf9c^OE^ vs. control CM. Properties of electrically evoked *Δ*F/F0 transients are summarized in Figure 5H. Median values for peak *Δ*F/F0, 10%–90% rise time, CaT_30_, CaT_50_, and CaT_90_ were all significantly reduced in Orf9c^OE^ vs. control CMs. Overall, these results indicate that Orf9c^OE^ altered mechanisms underlying [Ca^2+^]_i_ rise and decay of human CMs.

Next, multiple electrodes array (MEA) assays were performed to capture the field potential of spontaneously beating Orf9c^OE^ hPSC-CMs. While control hESC-CMs exhibited normal and rhythmic beating, Orf9c^OE^ hESC-CMs showed increased beating irregularities (Figures 5I and 5J). In MEAs assay, field potential duration (FPD) was used to assess the repolarization of beating CMs (Figure 5K). FPDs of Orf9c^OE^ hESC-CMs were shorter than those of control hESC-CMs (Figure 5L). The same result was observed in Orf9c^OE^ hiPSC-CMs (Figure S4B). Statistically, Orf9c^OE^ significantly reduced the beating rate (Figure 5M), elongated the beat period mean (Figure S4C), and shortened the FPDs (Figure S4D) of hESC-CMs. Spike amplitude, but not spike slope, was reduced by Orf9c^OE^ (Figure S4E). After FPD was adjusted for beating rate by using Bazett’s formula, the corrected FPD (FPD_c_) value was still significantly decreased in Orf9c^OE^ vs. control hESC-CMs (Figure 5N), suggesting that Orf9c altered the repolarization properties of hESC-CMs. Altogether, our data demonstrate that Orf9c^OE^ could compromise the calcium signaling, automaticity, and repolarization of hPSC-CMs.

### FDA-approved drugs ameliorate Orf9c-induced apoptosis and dysfunction of hPSC-CMs

**Figure 6:**
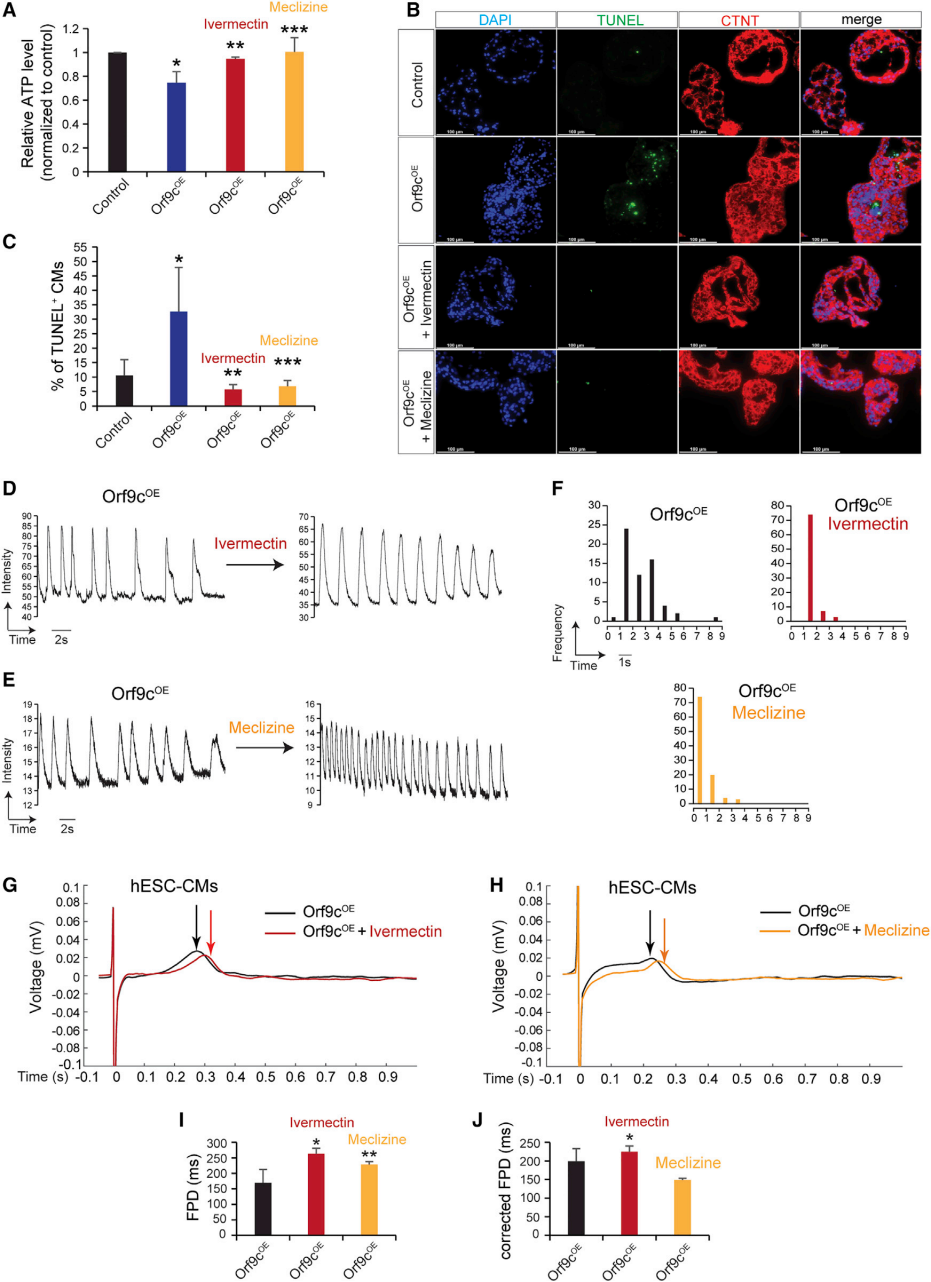
FDA-approved drugs ameliorate cell death and electrical dysfunction of Orf9c^OE^ hPSC-CMs (A) Quantification of cellular ATP level of hESC-CMs. Final concentration of ivermectin and meclizine was 0.5 μM. Treatment time was 3 h. All bars are shown as mean ± SD (n = 3). A two-tailed unpaired *t* test was used to calculate p values. ^∗^p < 0.05 (vs. control), ^∗∗^p < 0.05 (vs. Orf9c^OE^), ^∗∗∗^p < 0.05 (vs. Orf9c^OE^). (B) Representative immunostaining of TUNEL^+^ and CTNT^+^ CMs in hiPSC-EBs. Final concentration of ivermectin and meclizine was 0.5 μM. Treatment time was 48 h. (C) Statistical analysis of immunostaining results of (B). (D) Calcium imaging of hESC-CMs treated with ivermectin. The y axis shows intensity and the x axis represents time (in seconds, s). Ivermectin final concentration was 0.5 μM. Treatment time was 1 h. (E) Calcium imaging of hESC-CMs treated with meclizine. The y axis shows intensity and the x axis represents time (in seconds, s). Meclizine final concentration was 0.5 μM. Treatment time was 1 h. (F) ISI distribution data from (D and E). The y axis means beating frequency in a specific time window. The x axis means time (in seconds, s). (G and H) Analysis of data collected by multi-electrode arrays (MEAs) in Orf9c^OE^ hESC-CMs treated with ivermectin (G) or meclizine (H). Representative traces illustrate the change of FPD (ms) after treatment. Final concentration of ivermectin and meclizine was 0.5 μM. Treatment time was 2 h. (I) FPD of hESC-CMs. All bars are shown as mean ± SD (n = 4). A two-tailed unpaired *t* test was used to calculate p values. ^∗^p < 0.05 (vs. control), ^∗∗^p < 0.05 (vs. control). (J) The FPD_c_ of hESC-CMs. All bars are shown as mean ± SD (n = 4). A two-tailed unpaired *t* test was used to calculate p values. ^∗^p < 0.05 (vs. control).

Given the central role of cellular ATP level in viability of various cell types including CMs (Eguchi et al., 1997; Miyoshi et al., 2006; Tatsumi et al., 2003; Tsujimoto, 1997), we posited that enhancing ATP level could rescue Orf9c-induced CM abnormalities. We found two FDA-approved drugs, ivermectin (antiparasitic) and meclizine (antiemetic), were previously reported to protect mitochondrial function and sustain cellular ATP level (Caly et al., 2020; Gohil et al., 2010; Nagai et al., 2017). Interestingly, ivermectin (0.5 μM) or meclizine (0.5 μM) treatment for 3 h could significantly enhance the ATP level of Orf9c^OE^ hESC-CMs (Figure 6A). Importantly, 48 h of ivermectin (0.5 μM) or meclizine (0.5 μM) treatment prominently reduced the ratios of TUNEL^+^ Orf9c^OE^ hiPSC-CMs when compared with non-treated Orf9c^OE^ hiPSC-CMs (Figures 6B and 6C). These results demonstrate that ivermectin and meclizine could preserve cellular ATP levels to mitigate Of9c-induced apoptosis. Since reduced ATP levels could also impair intracellular Ca^2+^ signaling and excitation-contraction coupling of CMs (Bers, 2008; Bers and Guo, 2005; Fearnley et al., 2011), we next asked whether drug treatment would attenuate [Ca^2+^]_i_ abnormality of Orf9c^OE^ hESC-CMs. We found that ivermectin (Figure 6D) (0.5 μM) or meclizine (Figure 6E) (0.5 μM) treatment for 1 h prominently reduced the irregularity of [Ca^2+^]_i_ transients of Orf9c^OE^ hESC-CMs. Histogram analysis of the interspike intervals (ISIs) generated from [Ca^2+^]_i_ transient recordings showed that drug treatment markedly reduced the variations of ISIs of Orf9c^OE^ hESC-CMs (Figure 6F). Finally, ivermectin and meclizine did not affect the regular beating and FPD of control hESC-CMs (Figures S4F–S4F′), but prolonged the FPD of Orf9c^OE^ hESC-CMs when compared with non-treated hESC-CMs (Figures 6G and 6H). Similar results were observed in ivermectin- or meclizine-treated Orf9c^OE^ hiPSC-CMs (Figure S4G). A statistical analysis of MEA data showed ivermectin increased both FPD and FPD_c_, whereas meclizine only increased FPD of Orf9c^OE^ hESC-CMs (Figures 6I and 6J). Collectively, these results demonstrate that the FDA-approved drugs ivermectin and meclizine could ameliorate Orf9c-induced apoptosis and dysfunctions of hPSC-CMs.

## Discussion

Understanding the molecular etiology of cardiac disorders of patients with COVID-19 is critical for developing therapies. This study provides insights of the global impacts of Orf9c on transcriptome and proteome of host hPSC-CMs. Orf9c^OE^ in hPSC-CMs led to concordant transcriptomic changes with SARS-CoV-2-infected hPSC-CMs, particularly concentrated on apoptosis, inflammation, and the CM dysfunction signaling pathways. Orf9c^OE^ induced global protein expression changes and interacted with ATPase subunits, which reduced cellular ATP level of hPSC-CMs. Ivermectin and meclizine could restore ATP levels and ameliorate Orf9c^OE^-induced apoptosis and functional abnormalities of hPSC-CMs. Our findings thus provide mechanistic insights of the detrimental effects of a SARS-CoV-2 gene, Orf9c, on survival and function of human CMs.

COVID-19-associated cardiac manifestations include acute myocardial infarction, myocarditis, inflammatory response, acute heart failure, arrhythmia, hypoxemia, pulmonary embolism, and even stress cardiomyopathy, and multiple cardiac conditions could even coexist in the same patient (Shi et al., 2020b). Recently, Bailey et al. provided evidence that SARS-CoV-2 can directly and specifically infect CMs of patients with COVID-19 (Bailey et al., 2021). CM infection was identified by intramyocyte expressions of viral RNA and protein, and macrophage infiltration associated with areas of myocyte cell death. Although SARS-CoV-2 genome encodes 27 genes (Gordon et al., 2020), most studies were focused on the spike protein. Orf9c is one of the eight accessory proteins encoded by the SARS-CoV-2 genome, and interacts with mitochondrial and innate immune pathway proteins in human HEK293 cells (Gordon et al., 2020). In this study, we observed that Orf9c^OE^ in hPSC-CMs activated mRNA and protein levels of essential genes of immune and inflammation signaling pathways, and Orf9c interacted with ATPase subunits, which were consistent from previous findings from non-CMs. Notably, in hPSC-CMs, Orf9c^OE^ significantly reduced the ATP level and enhanced the protein levels of apoptotic markers, cleaved-CASP3 and cleaved-CASP9. Additionally, Orf9c impaired calcium handling and function of hPSC-CMs, which phenocopied SARS-CoV-2-infected hPSC-CMs showing enhanced apoptosis (Bojkova et al., 2020; Sharma et al., 2020) and electrical dysfunctions (Marchiano et al., 2021). Particularly, both Orf9c^OE^ and SARS-CoV-2 infection showed concordant impacts on transcriptome of hPSC-CMs (Figures 1E and 1F), with 531 elevated genes and 554 repressed genes by both conditions (Figure 1F). Considering the significant p values of 2.1 × 10^−80^ for up-regulated genes and 4.7 × 10^−69^ for down-regulated genes between Orf9c^OE^ and SARS-CoV-2, and the fact that those genes with concordant expression changes were concentrated into key signaling pathways for survival, function, and inflammation signaling pathways of human CMs, our results indicate that, of all SARS-CoV-2 viral genes, Orf9c might play a critical role in SARS-CoV-2-induced detrimental effects on human heart muscle cells. Interestingly, the integrated analysis of mRNA-seq datasets from Orf9c^OE^ hPSC-CMs and SARS-CoV-2-infected hPSC-CMs also identified more than 1,000 DEGs solely induced by SARS-CoV-2 but not by Orf9c^OE^ (Figure 1F), suggesting that the other SARS-CoV-2 genes might affect the transcriptome of host human CMs via different mechanisms than Orf9c, which will be further pursued.

An interactome analysis found that Orf9c interacted with ATPase subunits and compromised cellular ATP level of hPSC-CMs. Although the detailed mechanism requires further investigation, our results reveal the central role of ATP homeostasis in Orf9c-induced CM injuries, which is probably also important in SARS-CoV-2-casued tissue injuries of lung, kidney, and blood mononuclear cells (Ajaz et al., 2021). Besides an impaired cellular ATP level of hPSC-CMs, Orf9c^OE^ also led to the up-regulated expressions of proteins for mRNA splicing, and protein translation and transport (Figures 3B and 3C), which were observed in SARS-CoV-2-infected HEK293T cells (Gordon et al., 2020). A protein-protein interaction map analysis found Orf9c interacted with RPS15A, which catalyzes protein synthesis and is associated with viral mRNA translation and activation of the mRNA upon binding of the cap-binding complex and eIFs (Genuth and Barna, 2018; Li, 2019). All these findings suggest that Orf9c could broadly affect host human CMs through various mechanisms.

HPSC-CMs are not identical to mature CMs of adult human heart. Orf9 caused distinct alterations of CM [Ca^2+^]_i_ transients, including reduced peak amplitude and accelerated rise and decay times (Figure 5H). Our proteomic analyses revealed the reduced expressions of SERCA2 and RyR2 in Orf9c^OE^ hPSC-CMs (Table S2), suggesting the possibility that reduced SR Ca^2+^ content and/or density of the SR Ca^2+^ release channels might contribute to Orf9c-induced negative inotropy. In mature CMs, transverse tubular membranes enable the fast radial spread of the action potential, resulting in a spatiotemporally uniform SR Ca^2+^ release throughout the cell. However, it is known that hPSC-CMs typically exhibit an immature phenotype with a poorly developed tubular system (Guo et al., 2020). Thus, the monophasic and abbreviated [Ca^2+^]_i_ rise seen in Orf9c^OE^ hPSC-CMs could possibly be due to the altered organization of an immature transverse tubular network compared with that in the control hPSC-CMs. Although reduced expressions of SERCA2 and SLC8A1 were expected to slow diastolic recovery of [Ca^2+^]_i_, Orf9c^OE^ accelerated the [Ca^2+^]_i_ diastolic recovery of CMs. We can only speculate that this is a combined effect by Orf9c^OE^ to dysregulate the compensatory mechanisms to effectively offset the decreased SERCA2 and SLC8A1 in immature hPSC-CMs.

Ivermectin is one of a series of 16-membered macrocyclic lactone compounds discovered in 1967, and received FDA approval in 1987 for treating parasitic infections. Ivermectin was also found to enhance ATP production in the mitochondria of HL-1 CMs by up-regulating the transcription of Cox6a2, a subunit of the mitochondrial respiratory chain (Nagai et al., 2017). In this study, we observed that Orf9c^OE^ in hPSC-CMs down-regulated expressions of protein factors essential for mitochondrial ATP biosynthesis (Figure S2D) and respiratory electron transport chain, including multiple COX5/6/7 genes (Figure S2E), suggesting that ivermectin might mitigate apoptosis and dysfunction of Orf9c^OE^ hPSC-CMs at the transcription level. Additionally, a recent study reported that ivermectin could inhibit the replication of SARS-CoV-2 virus *in vitro* (Caly et al., 2020). However, a recent clinic study including 476 adult patients with COVID-19 with mild disease and symptoms found that a 5-day course of ivermectin did not significantly improve the time to resolution of symptoms when compared with placebo (Lopez-Medina et al., 2021). Although these findings suggested that ivermectin was not generally efficacious for treatment of patients with mild COVID-19 (Lopez-Medina et al., 2021), whether ivermectin could be used alone or in combination with other drugs for treating cardiac manifestations remains unknown, and more randomized controlled trials are required.

Meclizine is primarily used as an antihistamine. However, a nutrient-sensitized screening identified a cardio-protection effect of meclizine through promoting CM glycolysis to increase ATP synthesis (Gohil et al., 2010). Meclizine could stimulate glycolysis, mitigate ATP depletion, and protect mitochondrial function (Zhuo et al., 2016). These studies support our findings and can, at least in part, explain why meclizine could increase the ATP level to mitigate apoptosis of Orf9c^OE^ hPSC-CMs. Overall, our results indicate that maintaining ATP balance could possibly be a central target for the therapy of COVID-19-associated heart injuries and abnormalities.

## Experimental procedures

Detailed methods are provided in the supplemental experimental procedures.

### Human pluripotent stem cells culture and CM differentiation and purification

Human embryonic stem cell (hESC) line H9 and human induced pluripotent stem cell (hiPSC) line S3 were cultured on Matrigel (BD Biosciences)-coated plates in mTesR1 medium (Ludwig et al., 2006a, 2006b). For CM differentiation, the monolayer differentiation method was used based on a published protocol (Lian et al., 2013). After 14 days of differentiation, DMEM medium (no glucose) with 4 mM lactate (Sigma, #CAT-L1750-10G) was used to enrich CMs according to the published protocol (Tohyama et al., 2013). For EBs differentiation (3D differentiation), hPSCs were differentiated toward CMs using the previously established protocol (Lin et al., 2012; Yang et al., 2008). For drug treatment assay, all beating EBs were maintained in DMEM medium (no glucose, Gibco) with 10% FBS. The final concentration of ivermectin or meclizine was 0.5 μM (drugs were dissolved in DMSO). The drug treatment time for apoptosis assay was 2 days. All cytokines were from R&D Systems. All chemicals and drugs were from Sigma Aldrich.

### Lentivirus production and cell transduction

The lentiviral vector pLVX-EF1alpha-IRES-Puro-2xStrep-GFP or pLVX-EF1alpha-IRES-Puro-2xStrep-Orf9c (Addgene plasmid #141395 and #141393) was transfected into the HEK293T cells with packaging plasmids psPAX2 and pMD2.G using X-treme GENE nine transfection reagent (Roche). After 48 h, viral supernatant was collected and cellular debris was removed by syringe filtering (0.45 μm pore size; Millipore). H9 hESCs or S3 hiPSCs were incubated with virus supernatant (20% vol:vol) for 4 h and same infection was repeated after 24 h. Puromycin was added to select puromycin-resistant H9 hESCs or S3 hiPSCs clones after 48 h of viral infection.

### Whole mRNA-seq and proteomic experiment and data analysis

The mRNA-seq were conducted by Indiana University Genomic Core, and the mass spectrometry (MS) analysis, bioinformatics and data evaluation were performed in the Proteomics Core Facility at the Indiana University School of Medicine. Details are provided in the Supplemental information.

### Protein-protein interaction analysis

Protein-protein interaction networks analysis was carried out with the STRING software (https://string-db.org/).

### Quantification and statistical analysis

Data comparisons between two groups (gene overexpression versus control) were conducted using an unpaired two-tailed *t* test. All data were presented as mean ± SD from at least three independent experiments. Differences with *p* values of less than 0.05 were considered significant. The enrichment of overlap between two sets of genes, e.g., DE-Gs from Orf9c^OE^ and SARS-CoV-2 infection as shown in Figure 1F, was evaluated by the F.E., defined as the ratio of number of observed overlapping genes to number of expected overlaps by the random selections. The statistical significance was calculated by hypergeometric model.

### Data and code availability

The accession number for the mRNA-seq dataset reported in this paper is GSE171370.

## Author contributions

J.L. and L.Y. initiated and designed studies. J.L. performed most of experiments and data analyses. L.H., S.W., C.W., and H.C. assisted in CM differentiation, IHC, and RT-PCR. E.H.D. supported proteomics. Y.Z. and J.W. assisted in bioinformatics analyses. S.G. and M.L. supported calcium handling and MEA experiments and data interpretation. J.L., J.W., M.L., and L.Y. wrote and revised the manuscript.

## Conflicts of interest

The authors declare that they have no conflict of interest.

## Supplemental information

Document S1. Supplemental experimental procedures and Figures S1–S4

Table S1. Whole mRNA-seq data analysis

Table S2. Global proteomics data analysis

Table S3. CoIP-MS results of hPSC-CMs

Table S4. Oligonucleotides information

Document S2. Article plus supplemental information
