# Supplementary material for: Genome-wide analyses reveal the detrimental impacts of SARS-CoV-2 viral gene Orf9c on human pluripotent stem cell-derived cardiomyocytes
Source: Stem Cell Reports. 2022 Feb 17;17(3):522–37. doi: 10.1016/j.stemcr.2022.01.014 (PMC8851680; doi:10.1016/j.stemcr.2022.01.014)
Supplement: Document S1. Supplemental experimental procedures and Figures S1–S4 [file mmc1.pdf]

Stem Cell Reports, Volume *17*

Supplemental Information

Genome-wide analyses reveal the detrimental impacts of SARS-CoV-2 viral gene Orf9c on human pluripotent stem cell-derived cardiomyocytes

Juli Liu, Yucheng Zhang, Lei Han, Shuai Guo, Shiyong Wu, Emma Helen Doud, Cheng Wang, Hanying Chen, Michael Rubart-von der Lohe, Jun Wan, and Lei Yang

Supplemental experimental procedures

RT-qPCR

RNAs were isolated by RNeasy Kit (Qiagen, USA). CDNA synthesis was carried out using High-Capacity RNA-to-cDNA™ Kit (Applied Biosystems). RT-qPCR was performed on the QuantStudio 6 Real-Time PCR Systems with Fast SYBR Green Master Mix (Applied Biosystems, USA) according to the manufacturer's instructions. The RT-qPCR analysis were normalized to internal control GAPDH or beta-ACTIN using $2^{-\Delta\Delta Ct}$ method (Peltier and Latham, 2008). RT-qPCR data were presented with mean ± S.D. from at least three independent experiments. For all primer information, please see the Table S4 Oligonucleotides.

Western blotting

Proteins were extracted by a Complete™ Lysis-M EDTA-free kit (Roche, 04719964001, USA). Protein samples were mixed with NuPAGE™ LDS Sample Buffer (4X), boiled for 5 min at 95 °C, run on 4–15% Mini-PROTEAN TGX Gels (Bio-Rad, USA), transferred to PVDF membrane by using Trans-Blot® Turbo™ Transfer System (Bio-Rad, USA), blocked with 1X TBST buffer containing 10% not-fat milk, and incubated overnight at 4 °C with the corresponding primary antibodies with 5% BSA in 1X TBST buffer. Membranes were washed three times X 5 mins each with 1X TBXT buffer, and then incubated for 1 h at room temperature with horseradish-peroxidase-conjugated secondary antibodies in blocking solution with 5% BSA in 1X TBST buffer. Membranes were washed again three

times X 5 mins with 1X TBST, loaded with ECL Western Blotting Substrate (Pierce, USA), and imaged using a ChemiDoc Imaging System (Bio-Rad, USA).

Flow cytometry

Flow cytometry was performed as previously described (Lu et al., 2013). Briefly, CMs were harvested and dissociated by using 0.25% trypsin-EDTA at 37°C incubator for 10 mins. The dissociated cells were fixed in 4% PFA (diluted with 16% Paraformaldehyde (formaldehyde) aqueous solution) at room temperature for 10 mins and washed 3 times with 1X PBS. CMs were incubated in 1X blocking PBS buffer (containing 2% goat serum or 5% BSA plus 0.1% saponin) with corresponding primary antibodies at 37°C for 1h, following with corresponding secondary antibodies staining at 37°C for 1 hr. For TUNEL (Terminal deoxynucleotidyl transferase dUTP nick end labeling) experiments, staining was carried out by using an In Situ Cell Death Detection Kit, Fluorescein (11684795910 Roche, USA) according to the manual. Flow cytometry analysis was performed on an Attune NxT Flow Cytometer (Thermo Fisher Scientific, USA). Data were analyzed by using the FlowJo software (Treestar, USA).

Whole mRNA-seq

Total RNAs were extracted by using the RNeasy Kit (Qiagen, USA), followed with QC by using an Agilent Bioanalyzer 2100. One hundred nanograms of total RNAs were used for cDNA library preparation includes mRNA purification/enrichment, RNA fragmentation, cDNA synthesis, ligation of index adaptors and amplification, by following the KAPA

mRNA Hyper Prep Kit Technical Data Sheet, KR1352-v4.17 (Roche Corporate). Each resulting indexed library was quantified and its quality was accessed by Qubit and Agilent Bioanalyzer, and multiple libraries were pooled in equal molarity. The pooled libraries were then denatured and neutralized before being loaded to a NovaSeq 6000 sequencer at 300pM final concentration for 100b paired-end sequencing (Illumina, Inc.). Approximately 40-60M reads per library were generated, more than 90% of which reached a high Phred quality score, Q30 (corresponding to 99.9% base call accuracy). The mRNA-seq data could be accessed via the GEO number GSE171370.

mRNA-seq Data analyses

Quality control for raw mRNA-seq data was generated by FastQC v0.11.5 (http://www.bioinformatics.babraham.ac.uk/projects/fastqc/). Illumina adapter sequences and low-quality bases were trimmed by Trim Galore v0.4.5 (http://www.bioinformatics.babraham.ac.uk/projects/trim_galore/), followed by sequence mapping of high-quality paired-end reads to human genome (hg38) with the aligner STAR v2.7.2b (Dobin et al., 2013). We further used bam-filter in ngsutilsj v0.4.8 (https://compgen.io/ngsutilsj) to keep only properly and uniquely mapped paired reads (MAPQ ≥ 10) for downstream analysis. FeatureCounts from package subread v1.6.5 (Liao et al., 2019) was employed for quantification of gene expression by summarizing mapped reads to corresponding genes according to GENECODE v31 annotation. Analysis of differential expression genes (DEGs) was performed by edgeR v3.32.1(Robinson et al., 2010), with read counts normalized by the trimmed mean of M-values (TMM) method after lowly expressed genes filtered out by the filterByExpr function using default settings. DEGs due to Orf9c^{OE} were

identified if their FDR-adjusted *p*-values were less than 0.05 and amplitudes of fold changes (FCs) in base-2 log scale were larger than 0.2 given the comparison between the Orf9c^{OE} and the control. The gene expression profile of SARS-CoV-2-infected hiPSC-CMs (GSE150392) were achieved from NCBI GEO database (https://www.ncbi.nlm.nih.gov/geo/). The dataset includes raw read counts on genes from three hiPSC-CM samples infected with SARS-CoV-2 and three mock control hiPSC-CMs without virus infection (Sharma et al., 2020). DEG analysis was conducted by using edgeR with the same parameters used for the Orf9c^{OE} study. The cutoff of DEGs were set as FDR-adjusted *p*-values less than 0.05.

Co-immunoprecipitation mass spectrometry (Co-IP MS)

Beads were submitted to the IU Proteomics Core where they were covered in 8 M Urea, 50 mM Tris-HCl, pH 8.5, reduced with 5 mM tris (2-carboxyethyl) phosphine hydrochloride (TCEP) at room temperature for 30 mins and alkylated with 10 mM chloroacetamide (CAM) for 30 mins in dark at room temperature. Digestion was carried out using Trypsin/Lys-C Mass spec grade protease mix (Promega, V5072) at a 1:100 protease to substrate ratio, overnight at 37 °C. The reaction was quenched with 0.5 % formic acid prior to LC-MS. Samples were analyzed using a 5 cm trap column and 15 cm (2 μm particle size, 50 μm diameter) EasySpray (801A) column on an UltiMate 3000 HPLC and Q-Exactive Plus mass spectrometer (Thermo Fisher Scientific). Solvent B was increased from 5%-28% over 155 mins, to 35% over 5 mins, to 65% over 10 mins and back to 5% over 12 mins (Solvent A: 95% water, 5% acetonitrile, 0.1% formic acid; Solvent B: 100% acetonitrile, 0.1% formic acid). A data dependent top 20 method acquisition method was

used with MS scan range of 350-1600 m/z, resolution of 70,000, AGC target 3e6, maximum IT of 50 ms. MS2 settings of fixed first mass 100 m/z, normalized collision energy of 36, isolation window of 1.5 m/z, resolution of 35,000, target AGC of 1e5, and maximum IT of 250 ms. For dd acquisition, a minimum AGC of 2e3 and charge exclusion of 1, and ⩾7 were used.

Tandem Mass Tag-mass spectrometry (TMT-MS)

Cell and tissue preparation. Cells were lysed in 8 M urea, 50 mM Tris-HCl, pH 8.5. Samples were sonicated in a Bioruptor® sonication system from Diagende Inc. (30 sec/30 sec on/off cycles for 15 mins, 4 °C). Following centrifugation at 12,000 rpm for 15 mins, protein concentrations were determined using a Bradford protein assay (cat. num. 5000002, Bio-Rad). Protein samples in equal amounts (30 µg) were reduced with 5 mM TCEP and alkylated with 10 mM (CAM). Samples were diluted with 100 mM Tris-HCl to a final urea concentration of 2 M and digested overnight with Trypsin/Lys-C Mix Mass Spectrometry (1:100 protease: substrate ratio, cat. num. V5072, Promega) (Levasseur et al., 2019; Peck Justice et al., 2020; Plubell et al., 2017).

Peptide purification and labeling. Peptides were desalted on 50 mg Sep-Pak® Vac (Waters Corporation) employing a vacuum manifold. After elution from the column in 70% acetonitrile (ACN) 0.1% formic acid (FA), peptides were dried by speed vacuum and resuspended in 24 µL of 50 mM triethylammonium bicarbonate (TEAB). Peptide concentration was measured using Pierce Quantitative Colorimetric Peptide Assay Kit (cat. num. 23275, Thermo Fisher Scientific) to ensure that an equal amount of each

sample was labeled. Samples were then Tandem Mass Tag (TMT) labeled with 0.2 mg of reagent resuspended in 20 µL acetonitrile for two hours at room temperature (cat. num. 90309, Thermo Fisher Scientific TMT10plex™ Isobaric Label Reagent Set; lot no. UH285567 and 131C lot UD280157A). Labelling reactions were quenched with hydroxylamine at room temperature 15 minutes. Labelled peptides were then mixed and dried by speed vacuum.

High pH basic fractionation. The peptide mixture was resuspended in 0.1% TFA (trifluoroacetic acid) and fractionated on a Pierce^{TM} High pH reversed-phase peptide fractionation spin column using vendor methodology (cat. num. 84868). Each fraction was dried by speed vacuum and resuspended in 30 µL 0.1% FA.

Nano-LC-MS/MS Analysis. Nano-LC-MS/MS analyses were performed on an EASY-nLC™ HPLC system coupled to an Orbitrap Fusion™ Lumos™ mass spectrometer (Thermo Fisher Scientific). Half of each fraction was loaded onto a reversed phase PepMap™ RSLC C18 column with Easy-Spray tip at 400 nL/min (ES802A, 2 µm, 100 Å, 75 µm x 25 cm). Peptides were eluted from 4-33% B over 120 minutes, 33%-80% B over 5 mins, and dropping from 50-10%B over the final 4 min (Mobile phases A: 0.1% FA, water; B: 0.1% FA, 80% Acetonitrile). Mass spectrometer settings include capillary temperature of 300 °C and ion spray voltage was kept at 1.9 kV. The mass spectrometer method was operated in positive ion mode with a 4 second cycle time data-dependent acquisition with advanced peak determination and Easy-IC on (internal calibrant). Precursor scans (m/z 375-1600) were done with an orbitrap resolution of 120000, 30% RF lens, 105 ms maximum inject time (IT), standard automatic gain control (AGC) target. MS2 filters included an intensity threshold of 2.5e-4, charges states of 2 to 6, 70%

precursor fit threshold, and 60 s dynamic exclusion with dependent scan being performed on only one charge state per precursor. Higher-energy collisional dissociation (HCD) MS2 scans were performed at 50k orbitrap resolution, fixed collision energy of 37%, 200% normalized AGC target, and dynamic maximum IT.

Data analysis. Resulting RAW files were analyzed in Proteome Discover™ 2.4 (Thermo Fisher Scientific) with FASTA databases including Swiss-Prot UniProt *Homo sapiens* sequences (downloaded 09/17/2019) plus common contaminants. SEQUEST HT searches were conducted with a maximum number of 2 missed cleavages; precursor mass tolerance of 10 ppm; and a fragment mass tolerance of 0.02 Da. Static modifications used for the search were, 1) carbamidomethylation on cysteine (C) residues; 2) TMT sixplex label on lysine (K) residues and the N-termini of peptides (for TMT quant samples only). Dynamic modifications used for the search were oxidation of M, phosphorylation on S, T, Y, and acetylation of N-termini. IP-MS Sequest results were imported into Scaffold (Proteome Software) for Fishers exact test comparison. TMT quantification methods utilized isotopic impurity levels available from Thermo Fisher. Percolator False Discovery Rate was set to a strict setting of 0.01 and a relaxed setting of 0.05. Values from both unique and razor peptides were used for quantification. In the consensus workflow, peptides were normalized by total peptide amount with no scaling. Resulting abundance values for each sample, and abundance ratio values from Proteome Discoverer™ were exported to Microsoft Excel and are available in a supplemental file.

Apoptosis assay

Apoptosis analysis of live cells was performed using Annexin-V-FLUOS Staining Kit (11858 777001, Roche, USA) according to the manual. Briefly, cells were harvested and dissociated by using 0.25% trypsin-EDTA at 37°C incubator for 10 mins. Single cells were washed in 1X PBS, then resuspend and incubated in 100 µl of Annexin-V-FLUOS labeling solution for 15 mins at room temperature, followed with the analysis on BD LSRII cytometer (Becton Dickinson, USA) or Attune NxT Flow Cytometer (Thermo Fisher Scientific, USA). Data were analyzed by using FlowJo software (Treestar, USA).

ATP detection assay

The cellular ATP level was measured by using the Luminescent ATP Detection Assay Kit (ab113849, Abcam, USA) according to the manual. Briefly, a hemocytometer was used to count the number of live cells. The same number of cells for each group was used for the ATP quantification. Live cells were resuspended in 50 µl detergent solution with 1200 rpm shaking on Eppendorf ThermoMixer C for 5 mins at room temperature. Then 50 µl substrate solution was added to the detergent solution with 1200 rpm shaking on Eppendorf ThermoMixer C for 5 mins at room temperature. All solution was transferred to Nunc™ MicroWell™ 96-Well, (Nunclon Delta-Treated, Flat-Bottom Microplate, White Polystyrene Plate, Thermo Scientific, 136101, USA), followed with luminescence detection on GloMax Discover Microplate Reader (GM3000, Promega, USA). For drug treatment assays, the final concentration of ivermectin or meclizine was 0.5 µM, and the treatment time was 3 hrs.

Intracellular calcium imaging

For confocal Ca^{2+} imaging, $[Ca^{2+}]_i$ transients were recorded in hPSC-CMs loaded with fluorescent Ca^{2+} dye fluo-4 AM, on a laser scanning confocal microscope (Olympus Fluoview 1000) equipped with a 60x oil immersion lens (NA = 1.4). Fluo-4 (loaded by 20 min incubation with 10 μM of acetoxymethylester dissolved in normal Tyrode's solution supplemented with F-127. Fluo-4 was excited with the 488-nm line of an argon laser. Fluorescence was collected through a 515- to 560-nm bandpass filter. Fluo-4 images were recorded in line-scan mode with 512 pixels per line at line rates ranging from 1.5 to 2 ms/line. To record cell-wide $[Ca^{2+}]_i$ transients, cells were excited at 0.5 Hz by field stimulation (20-40 V, 4 ms) using two parallel platinum electrodes. To determine the properties of electrically evoked changes in fluo-4 fluorescence, we first calculated the average signal intensity of each successive line in a line-scan image, to obtain the time course of the line average fluorescence, F(t). The F(t) traces were condensed into one-cycle recordings by averaging over three consecutive transients, and converted to $\Delta F/F_0$(t), where F_0 is the average F over the time before the Ca^{2+} rise, and ΔF equals $F_{(t)}$-F_0. From the averaged $\Delta F/F_0$(t) we determined the magnitudes of peak $\Delta F/F_0$, 10-90% rise time, and 30%, 50% and 90% recovery times (CaT_{30}, CaT_{50}, CaT_{90}; computed as the interval from peak ΔF/F0 to the time when ΔF/F0 is 30, 50 or 90% recovered). For Calcium irregularity analysis, hPSC-CMs were attached on 12-well plate coated with Matrigel and loaded with X-Rhod-1 (X14210, Invitrogen, USA) in DMEM medium (no glucose) with 10% FBS containing Pluronic F-127 (final concentration 0.02%, P2443, Sigma, USA) for 15 mins at 37 °C incubator. Videos were acquired at a rate of 50 frames per second using the All-in-One Fluorescence Microscope BZ-X800 (KEYENCE CORPORATION, Japan).

For drug treatment assay, the final concentration of ivermectin or meclizine was 0.5 μM (drugs were dissolved in DMSO). The calcium handling signaling was acquired after 1 hr. of drug treatment. Videos were analyzed by ImageJ software using a custom script that calculated the temporal changes in calcium fluorescence intensity.

Multi-electrode arrays (MEAs)

MEAs assay was performed with cardiomyocytes after 30 days of differentiation. All experiments were performed in DMEM medium (no glucose, Gibco, USA) with 10% FBS. Cardiomyocytes were attached on the cytoview MEA 24-well white plate (M384-tMEA-24W-5, Axion Biosystems, USA) coated with Matrigel. Electrophysiology data were acquired by using the Maestro Edge MEA System (Axion Biosystems, USA) with the integrated environment chamber controlling the heat at 37 °C and CO_2 at 5%. The Interspike interval was the time duration of two adjacent spikes (beating peaks). For the Interspike interval (ISI) distribution analysis, we calculated both of the number of spikes (beating peaks) and the ISI. Then we grouped the ISI as the time window 0-1s, 1-2s, 2-3s,3-4s, 4-5s, 5-6s, 6-7s and 7-8s and calculated the spike number frequency according to every time window to generate ISI distribution. In the Interspike interval (ISI) distribution analysis results figures. Y-axis means spikes (beating peaks) number (frequency) in a specific time window. X-axis means time (in second, s).

Immunofluorescence

For immunostaining of attached cells, cells were fixed with 4% PFA (diluted from 16% Paraformaldehyde aqueous solution) for 10 mins at room temperature. After washing with 1X PBS, cells were blocked for 1 h with 1X PBS blocking buffer containing 2% goat serum (or 5% BSA) and 0.1% saponin. Staining with corresponding primary antibodies diluted with blocking buffer was performed at 4°C for overnight. Staining with secondary antibodies were performed on the next day, following with nucleus staining with DAPI. For beating EBs immunostaining, live EBs were fixed with 4% PFA (diluted from 16% Paraformaldehyde aqueous solution) for 10 mins at room temperature, followed with 15% sucrose solution at room temperature until all EBs sunk. Then EBs were embed in OCT and cryosectioning was performed. The EBs immunostaining protocol was the same as that of attached cells. For TUNEL (Terminal deoxynucleotidyl transferase dUTP nick end labeling) experiments, staining of attached cells or EBs sections were carried out using In Situ Cell Death Detection Kit, Fluorescein (11684795910 Roche, USA) according to the manual. Leica DM6B image system was used for imaging.

Functional enrichment analysis

The GO enrichment analysis was performed by using DAVID (https://david.ncifcrf.gov/), with assistance from Gene Ontology enRIchment anaLysis and visuaLizAtion tool (http://cbl-gorilla.cs.technion.ac.il/). The canonical signaling pathway and toxicity analysis were performed by Ingenuity Pathway Analysis (QIAGEN IPA).

Supplemental References

Dobin, A., Davis, C.A., Schlesinger, F., Drenkow, J., Zaleski, C., Jha, S., Batut, P., Chaisson, M., and Gingeras, T.R. (2013). STAR: ultrafast universal RNA-seq aligner. Bioinformatics *29*, 15-21.

Levasseur, E.M., Yamada, K., Pineros, A.R., Wu, W., Syed, F., Orr, K.S., Anderson-Baucum, E., Mastracci, T.L., Maier, B., Mosley, A.L., et al. (2019). Hypusine biosynthesis in beta cells links polyamine metabolism to facultative cellular proliferation to maintain glucose homeostasis. Sci Signal *12*. eaax0715.

Liao, Y., Smyth, G.K., and Shi, W. (2019). The R package Rsubread is easier, faster, cheaper and better for alignment and quantification of RNA sequencing reads. Nucleic Acids Res *47*, e47.

Lu, T.Y., Lin, B., Li, Y., Arora, A., Han, L., Cui, C., Coronnello, C., Sheng, Y., Benos, P.V., and Yang, L. (2013). Overexpression of microRNA-1 promotes cardiomyocyte commitment from human cardiovascular progenitors via suppressing WNT and FGF signaling pathways. J Mol Cell Cardiol *63*, 146-154.

Peck Justice, S.A., Barron, M.P., Qi, G.D., Wijeratne, H.R.S., Victorino, J.F., Simpson, E.R., Vilseck, J.Z., Wijeratne, A.B., and Mosley, A.L. (2020). Mutant thermal proteome profiling for characterization of missense protein variants and their associated phenotypes within the proteome. J Biol Chem. *295*, 16219-16238.

Peltier, H.J., and Latham, G.J. (2008). Normalization of microRNA expression levels in quantitative RT-PCR assays: Identification of suitable reference RNA targets in normal and cancerous human solid tissues. Rna *14*, 844-852.

Plubell, D.L., Wilmarth, P.A., Zhao, Y., Fenton, A.M., Minnier, J., Reddy, A.P., Klimek, J., Yang, X., David, L.L., and Pamir, N. (2017). Extended Multiplexing of Tandem Mass Tags (TMT) Labeling Reveals Age and High Fat Diet Specific Proteome Changes in Mouse Epididymal Adipose Tissue. Mol Cell Proteomics *16*, 873-890.

Robinson, M.D., McCarthy, D.J., and Smyth, G.K. (2010). edgeR: a Bioconductor package for differential expression analysis of digital gene expression data. Bioinformatics *26*, 139-140.

Sharma, A., Garcia, G., Wang, Y.Z., Plummer, J.T., Morizono, K., Arumugaswami, V., and Svendsen, C.N. (2020). Human iPSC-Derived Cardiomyocytes Are Susceptible to SARS-CoV-2 Infection. Cell Rep Med *1*. 100052.

Legends of Supplementary Tables

Table S1. Whole mRNA-seq data analysis

Table S2 Global proteomics data analysis

Table S3 CoIP-MS results of hPSC-CMs

Table S4 Oligonucleotides information

REAGENT or RESOURCE	SOURCE	IDENTIFIER
**Antibodies**		
Rabbit IgG	Millipore	MAGNARIP01, RRID:AB_10681285
Mouse IgG	Millipore	MAGNARIP01, RRID:AB_2617156
Mouse IgG Isotype Control	R&D system	MAB002, RRID:AB_357344
Rabbit IgG	Cell Signaling Technology (CST)	#2729, RRID:AB_1031062
Alexa Fluor® 647 Mouse Anti-Cardiac Troponin T	BD Pharmingen	# 565744, RRID:AB_2739341
Cardiac Troponin T antibody (mouse)	Thermo Fisher	MS-295-P, RRID:AB_61806
APC goat anti-mouse IgG	BD	550826, RRID:AB_398465
Strep Tag Monoclonal Antibody (GT661)	Thermo Fisher Scientific	# MA5-17283, RRID:AB_2538749
Anti-Oct4 antibody	Cell Signaling Technology (CST)	Cat# 5177, RRID:AB_10693303
Cleaved Caspase-3 (Asp175) (5A1E) Rabbit mAb	Cell Signaling Technology (CST)	# 9664, RRID:AB_2070042
Anti-CASP3 antibody	neomarkers	RB-1197-P0, RRID:AB_60471
Anti-GAPDH Mouse mAb (6C5)	Sigma-Aldrich	CB1001, RRID:AB_2107426
Anti-cleaved CASP9 antibody (D315)	Cell Signaling Technology (CST)	9505P, RRID:AB_2290727
Anti-CASP9 antibody	Cell Signaling Technology (CST)	9502P, RRID:AB_2068621
Goat anti-Mouse IgG (H+L) Cross-Adsorbed Secondary Antibody, Alexa Fluor 647	Invitrogen	A-21235, RRID:AB_2535804
Goat anti-Rabbit IgG (H+L), Secondary Antibody, Alexa Fluor 488	Invitrogen	A27034, RRID:AB_2536097

Goat anti-Mouse IgG (H+L) Cross-Adsorbed Secondary Antibody, Cyanine5	Invitrogen	A10524, RRID:AB_2534033
**Chemicals, Peptides, and Recombinant Proteins**		
RhBMP4	R&D system	314-BP
RhFGF2	R&D system	233-FB
RActivin A	R&D system	338-AC
XAV 939	R&D system	3748
Meclizine hydrochloride	Sigma-Aldrich	1377009
Ivermectin	Sigma-Aldrich	I8898
X-Rhod-1, AM, cell permeant	Invitrogen	X14210
Pluronic® F-127	Sigma-Aldrich	P2443
DreamTaq Green PCR Master Mix (2X)	Thermo Fisher Scientific	K1081
L-(+)-Lactic acid	Sigma-Aldrich	L1750
CHIR99021	R&D system	4423
Saponin	Sigma	S4521-25G
L-Ascorbic acid 2-phosphate sesquimagnesium salt hydrate	Sigma-Aldrich	A8960-5G
Puromycin dihydrochloride	Sigma-Aldrich	P8833-10MG
Polybrene	Sigma-Aldrich	H9268-5G
TrypLE™ Express Enzyme (1X), phenol red	Gibco	12605010
DAPI Fluoromount-G	southernbiotech	0100-20
UltraPure™ 0.5M EDTA, pH 8.0	Thermo Fisher Scientific	15575020
Fast SYBR Green Master Mix	Applied Biosystems	4385612
**Critical Commercial Assays**		
B-27™ Supplement, minus insulin	Thermo Fisher Scientific	A1895601
B-27™ Supplement (50X), serum free	Thermo Fisher Scientific	17504044
4–20% Mini-PROTEAN® TGX™ Precast Protein Gels (60X)	Bio-rad	4561096
SuperSignal™ West Atto Ultimate Sensitivity Substrate	Thermo Fisher Scientific	A38555
In Situ Cell Death Detection Kit, TMR red	Roche	12156792910
Luminescent ATP Detection Assay Kit	abcam	ab113849
High-Capacity RNA-to-cDNA™ Kit	Applied Biosystems	4387406
Classic Magnetic IP/Co-IP Kit	Pierce	88804
RPMI 1640 Medium	Gibco	11875093
DMEM/F12	Gibco	11320082

DMEM, high glucose	Gibco	11965118
DMEM, no glucose	Gibco	11966025
StemPro™-34 SFM (1X)	Gibco	10639011
Pierce™ ECL Western Blotting Substrate	Pierce	32209
cOmplete™ Lysis-M EDTA-free	Roche	04719964001
RNeasy Mini Kit	Qiagen	74106
QIAquick Gel Extraction Kit<br>Print	Qiagen	28706
QIAquick PCR Purification Kit<br>Print	Qiagen	28106
Corning® Matrigel® Growth Factor Reduced (GFR) Basement Membrane Matrix, *LDEV-Free, 10mL	Corning	354230
**Experimental Models: Cell Lines**		
293T cells	ATCC	CRL-3216
Wildtype Human iPSC line S3	Carvajal-Vergara et al., 2010	N/A
Wildtype Human H9 hESCs	ATCC	HTB-176
Human S3 iPSCs with overexpression of SARS-CoV-2 gene Orf9c<br>Human S3 iPSCs with overexpression of EGFP	This paper	N/A
Human H9 hESCs with overexpression of SARS-CoV-2 gene Orf9c<br>Human H9 hESCs with overexpression of EGFP<br>Human H9 hESCs with overexpression of blank lentivirus backbone	This paper	N/A
**Oligonucleotides**		
Primers see Table S oligonucleotides	This paper	N/A
**Recombinant DNA**		
pLVX-EF1alpha-eGFP-2xStrep-IRES-Puro	Addgene, please see the citation [6].	Addgene number: 141395
pLXV-EF1alpha-2xStrep-SARS-CoV-2-orf9c-IRES-Puro	Addgene, please see the citation [6].	Addgene number: 141393
psPAX2	From Dr. Gang Hu lab (NIH)	N/A
pMD2.G	From Dr. Gang Hu lab (NIH)	N/A
**Software and Algorithms**		
Image J	National Institutes of Health	https://imagej.nih.gov/ij/

Ingenuity Pathway Analysis (IPA)	QIAGEN	https://www.qiagen.com/us/products/discovery-and-translational-research/next-generation-sequencing/informatics-and-data/interpretation-content-databases/ingenuity-pathway-analysis/?clear=true#orderinginformation
GENE ONTOLOGY	THE GENE ONTOLOGY RESOURCE	http://geneontology.org/
STRING	STRING CONSORTIUM	https://string-db.org
Adobe Illustrator	Adobe Inc.	https://www.adobe.com/products/illustrator.html
FlowJo (Treestar)	FlowJo, LLC	https://www.flowjo.com/about/company
**Bacterial and viral strains**		
MAX Efficiency™ DH5α Competent Cells	Invitrogen	18258012
One Shot™ Stbl3™ Chemically Competent E. coli	Invitrogen	C737303

Supplementary Figure 1

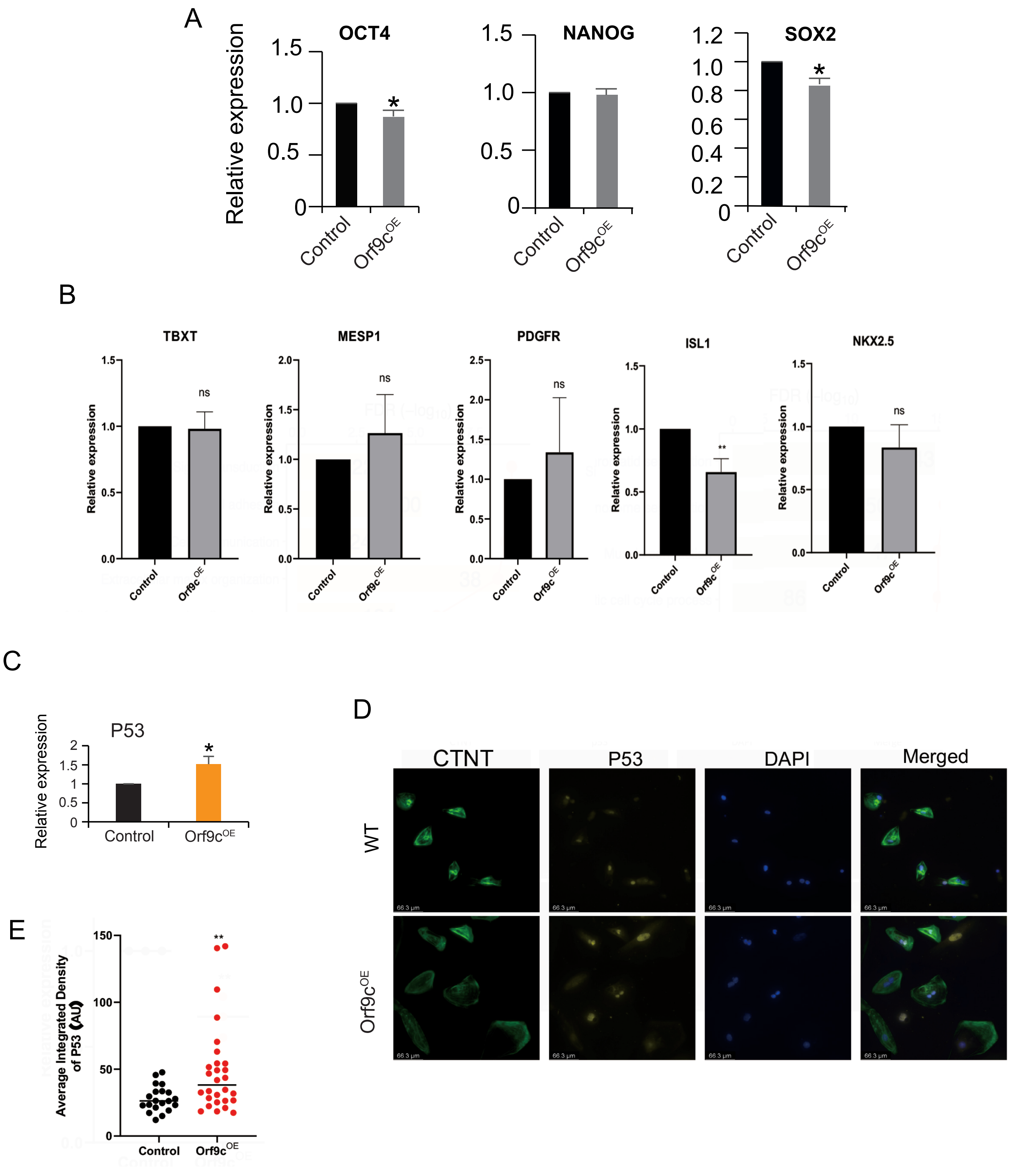

Supplementary Figure 1. Effect of Orf9c on gene expressions of hPSC-CMs.
(A) RT-qPCR analysis of the expression levels of pluripotency marker genes. n=3. t-test. *$p < 0.05$.
(B) RT-qPCR analysis of the expression levels of cardiac marker genes. n=3. t-test. **$p < 0.01$.
(C) RT-qPCR analysis of the P53 in control and Orf9cOE hPSC-CMs. n=3. t-test. *$p < 0.05$.
(D) Representative immunostaining of P53 in in control and Orf9cOE hPSC-CMs, with statistical analysis of fluorescence desnsity of P53 in CMs of (E). t-test. **$p < 0.01$.

Supplementary Figure 2

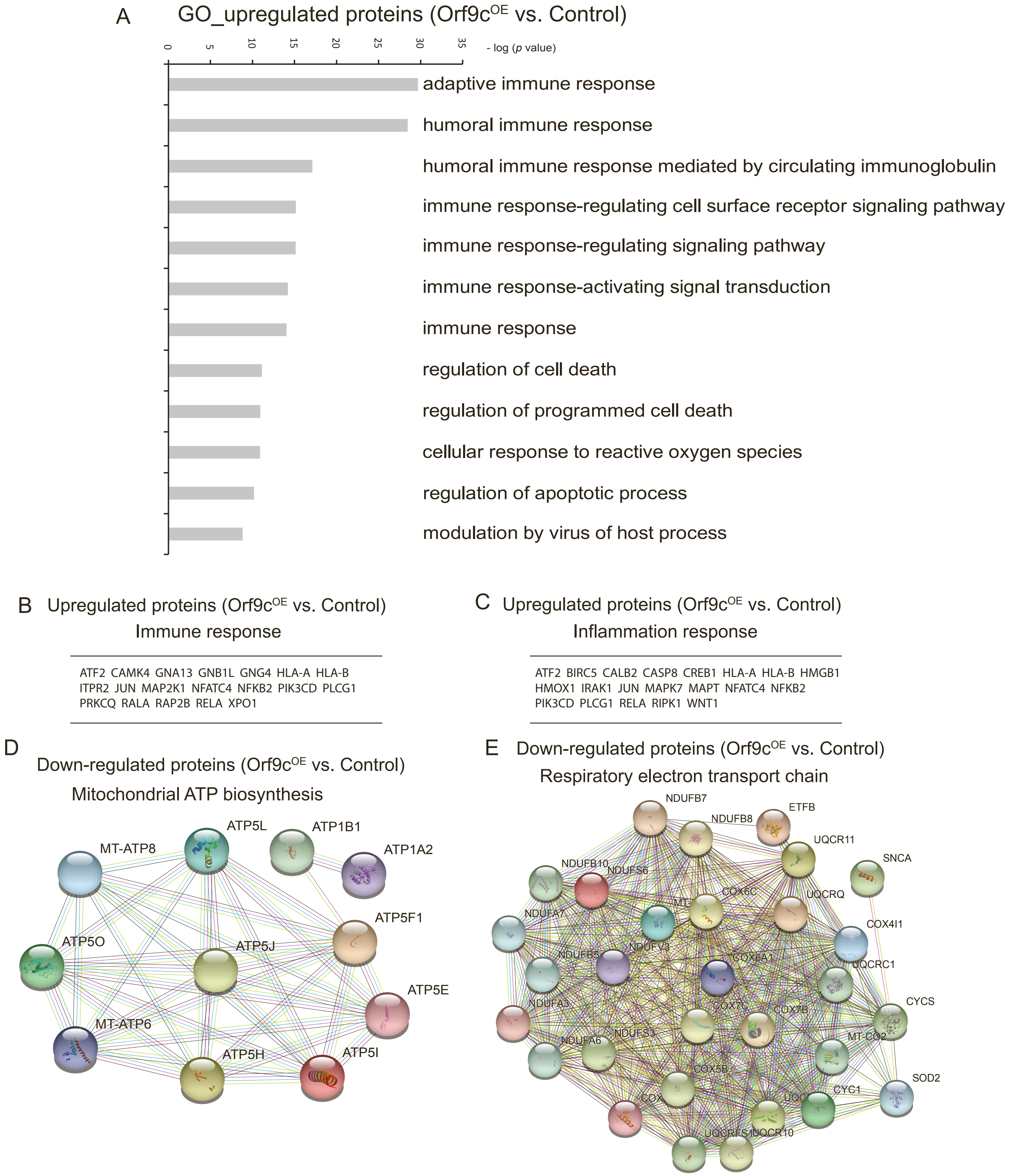

Supplementary Figure 2. Genome-wide proteomics and protein-protein interaction analysis reveals proteins with differential expression levels induced by SARS-CoV-2 gene Orf9c.
(A) Gene Ontology (GO) analysis of upregulated proteins (Orf9cOE vs. Control) in hiPSC-CMs.
(B) Upregulated proteins involved in immune response by Orf9c overexpression in hiPSC-CMs.
(C) Upregulated proteins involved in inflammation response induced by Orf9c overexpression in hiPSC-CMs.
(D) Protein-protein interaction analysis showing down-regulated proteins, induced by Orf9c overexpression in hiPSC-CMs, associate with mitochondrial ATP biosynthesis.
(E) Protein-protein interaction analysis showing down-regulated proteins, induced by Orf9c in hiPSC-CMs, associate with respiratory electron transport chain.

Supplementary Figure 3

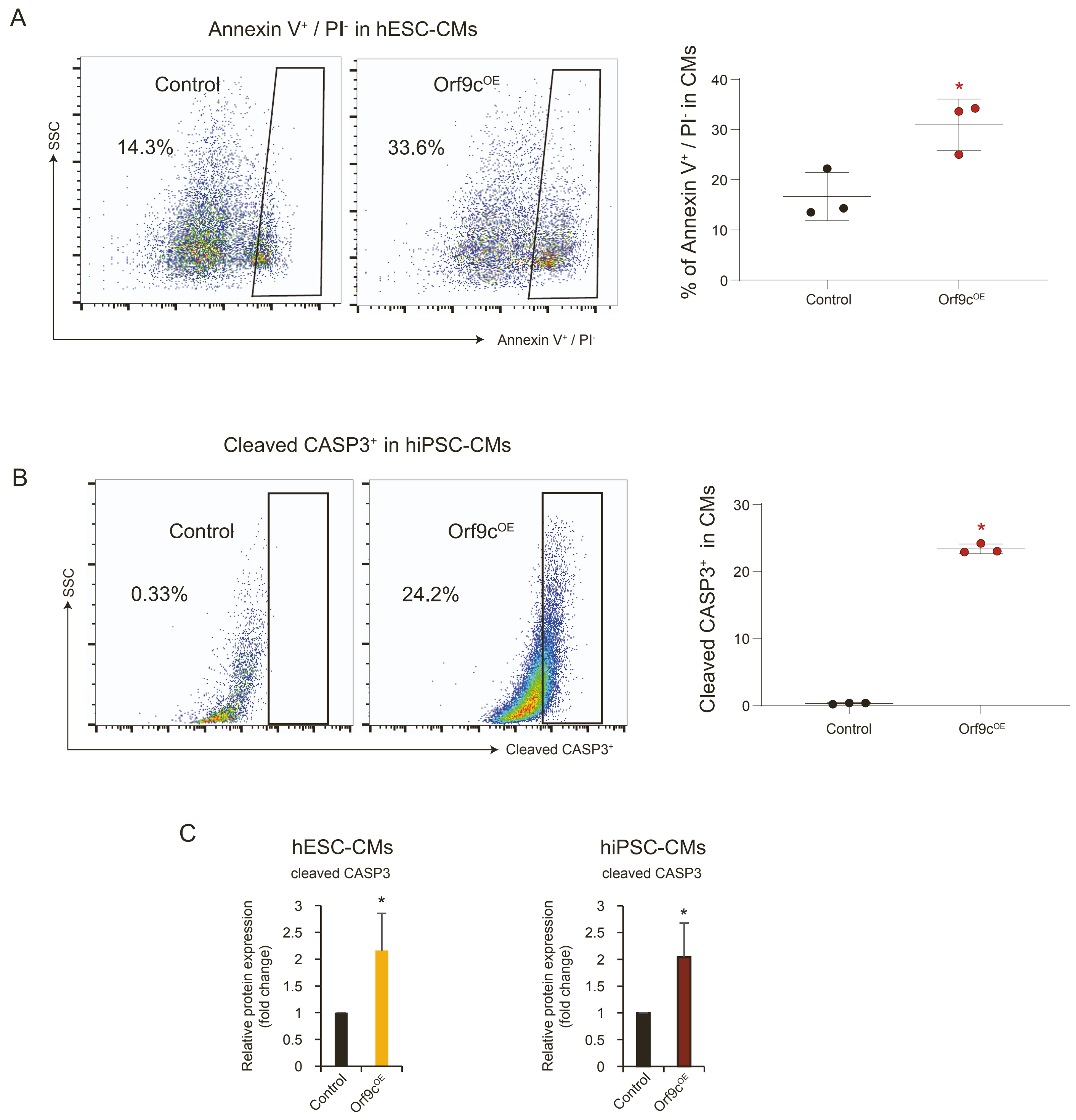

Supplementary Figure 3. Orf9c induces cell death in hPSC-CMs.

(A) Flow cytometry analysis of Annexin V+ / PI- CMs in CM population of hESC-CMs. All bars are shown as mean ± SD. (n=3). A two-tailed unpaired t-test was used to calculate P-values: *$p < 0.05$ (vs. Control).

(B) Flow cytometry analysis of Cleaved-CASP3+ CMs in CM population of hiPSC-CMs. All bars are shown as mean ± SD. (n=3). A two-tailed unpaired t-test was used to calculate P-values: *$p < 0.05$ (vs. Control).

(C) Statistical results of the cleaved CASP3 protein levels in control and Orf9c-OE hPSC-CMs. A two-tailed unpaired t-test was used to calculate P-values: *$p < 0.05$ (vs. Control).n=3.

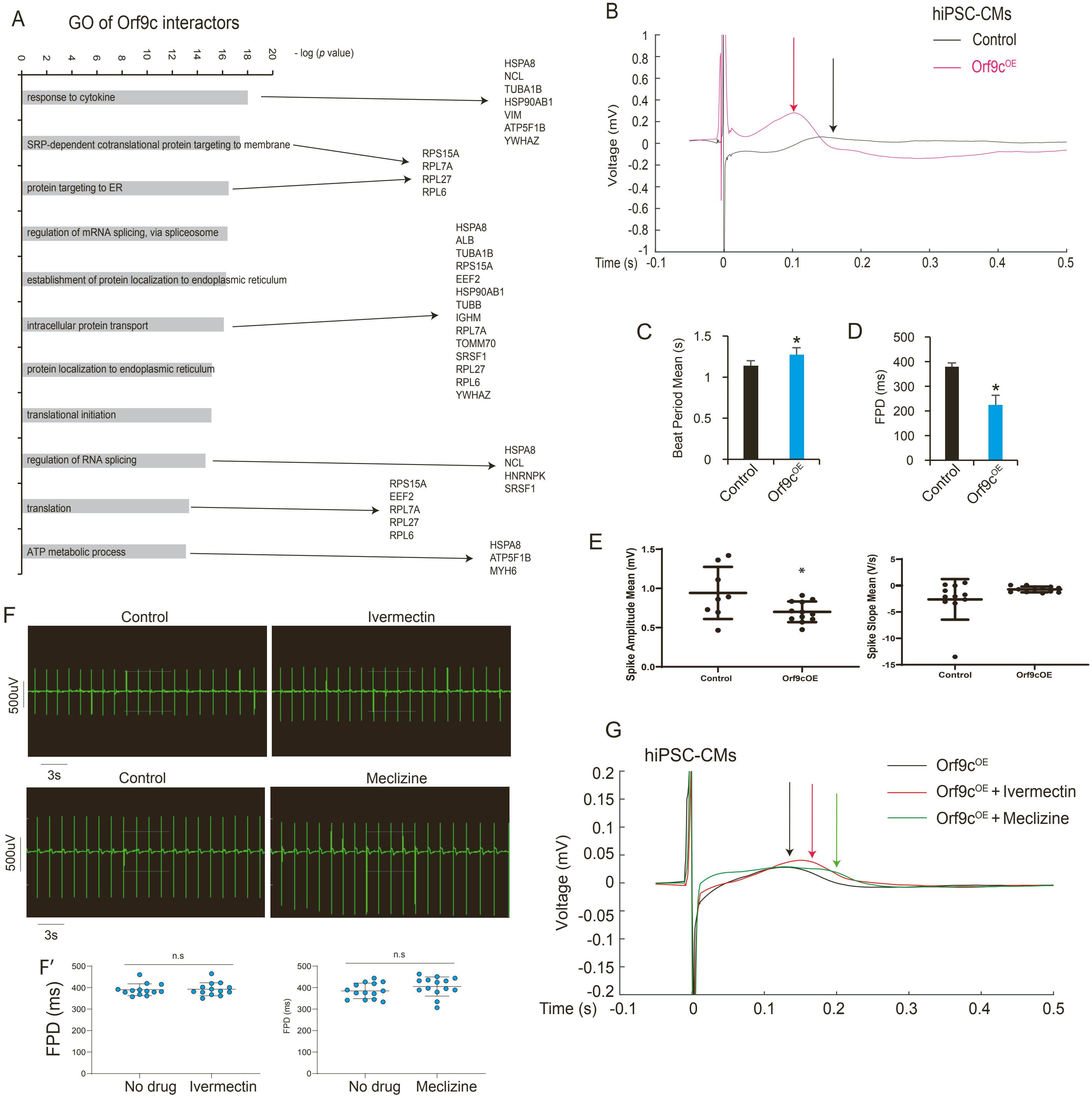

Supplementary Figure 4. The function of Orf9c in hPSC-CMs and FDA-approved drugs elongated FPDs of Orf9cOE hPSC-CMs.

(A) Gene Ontology (GO) analysis of Orf9c interactors in hESC-CMs.

(B) Representative traces illustrate the change of field potential duration (FPD, ms) in Orf9c-OE vs. control hiPSC-CMs.

(C) Beat Period Mean of MEA data in hESC-CMs. Bars are shown as mean ± SD. (n = 4). A two-tailed unpaired t-test was used to calculate P-values. *p < 0.05.

(D) Field potential duration (FPD) analysis of MEA data in hESC-CMs. *p < 0.05. Same statistical method as (C).

(E) Mean spike amplitude and slope of Orf9cOE vs. control hESC-CMs. **p* < 0.05. t-test.

(F) Representative MEA recordings of spontaneous electrical activities of control hESC-CMs without and with drug treatment, with statistical results of FPD in F′. Final concentration of ivermectin and meclizine was 0.5 μM. Treatment time was 2 hrs. n.s., no significance. Same statistical method as (C).

(G) Representative traces illustrate the change of field potential duration (FPD, ms) in Orf9cOE hiPSC-CMs with vs. without drug treatments. Ivermectin and Meclizine final concentrations were 0.5 μM. Treatment time was 2 hrs.
