# Supplementary material for: Genome-wide analyses reveal the detrimental impacts of SARS-CoV-2 viral gene Orf9c on human pluripotent stem cell-derived cardiomyocytes
Source: Stem Cell Reports. 2022 Feb 17;17(3):522–37. doi: 10.1016/j.stemcr.2022.01.014 (PMC8851680; doi:10.1016/j.stemcr.2022.01.014)
Supplement: Document S2. Article plus supplemental information [file mmc6.pdf]

Stem Cell Reports
Article
OPEN ACCESS

Genome-wide analyses reveal the detrimental impacts of SARS-CoV-2 viral gene Orf9c on human pluripotent stem cell-derived cardiomyocytes

Juli Liu,[1,4] Yucheng Zhang,[2] Lei Han,[1] Shuai Guo,[1] Shiyong Wu,[1] Emma Helen Doud,[3] Cheng Wang,[1] Hanying Chen,[2] Michael Rubart-von der Lohe,[1] Jun Wan,[2] and Lei Yang[1,*]
[1]Department of Pediatrics, Indiana University School of Medicine, Herman B Wells Center for Pediatric Research, Indianapolis, IN 46202, USA
[2]Department of Medical and Molecular Genetics, Indiana University School of Medicine, Indianapolis, IN 46202, USA
[3]Department of Biochemistry and Molecular Biology, Indiana University School of Medicine, Indianapolis, IN 46202, USA
[4]Present address: Research Center of Medical Sciences, Guangdong Provincial People's Hospital, Guangdong Academy of Medical Sciences, 510080, Guangzhou, Guangdong, China
*Correspondence: lyang7@iu.edu
https://doi.org/10.1016/j.stemcr.2022.01.014

SUMMARY

Patients with coronavirus disease 2019 (COVID-19) commonly have manifestations of heart disease. Severe acute respiratory syndrome coronavirus 2 (SARS-CoV-2) genome encodes 27 proteins. Currently, SARS-CoV-2 gene-induced abnormalities of human heart muscle cells remain elusive. Here, we comprehensively characterized the detrimental effects of a SARS-CoV-2 gene, Orf9c, on human pluripotent stem cell-derived cardiomyocytes (hPSC-CMs) by preforming multi-omic analyses. Transcriptomic analyses of hPSC-CMs infected by SARS-CoV-2 with Orf9c overexpression (Orf9c^{OE}) identified concordantly up-regulated genes enriched into stress-related apoptosis and inflammation signaling pathways, and down-regulated CM functional genes. Proteomic analysis revealed enhanced expressions of apoptotic factors, whereas reduced protein factors for ATP synthesis by Orf9c^{OE}. Orf9c^{OE} significantly reduced cellular ATP level, induced apoptosis, and caused electrical dysfunctions of hPSC-CMs. Finally, drugs approved by the U.S. Food and Drug Administration, namely, ivermectin and meclizine, restored ATP levels and ameliorated CM death and functional abnormalities of Orf9c^{OE} hPSC-CMs. Overall, we defined the molecular mechanisms underlying the detrimental impacts of Orf9c on hPSC-CMs and explored potentially therapeutic approaches to ameliorate Orf9c-induced cardiac injury and abnormalities.

INTRODUCTION

Since the end of 2019, severe acute respiratory syndrome coronavirus 2 (SARS-CoV-2) has infected more than 250 million people in the world and caused more than five million deaths world widely and more than 774,000 deaths in the United States. SARS-CoV-2 infects cells via binding angiotensin-converting enzyme 2 (ACE2), as well as transmembrane serine protease 2 (TMPRSS2) (Hoffmann et al., 2020) and TMPRSS4 (Zang et al., 2020). ACE2 is highly expressed in human lung, intestine, and heart (Hikmet et al., 2020), which could potentially account for SARS-CoV-2-induced respiratory symptoms (Zhu et al., 2020) and kidney (Cheng et al., 2020) and cardiovascular diseases (Guo et al., 2020). Although the primary cause of mortality by SARS-CoV-2 infection is respiratory failure, cardiac complications, including myocarditis and arrhythmias, prominently contribute to the overall mortality (Guo et al., 2020; Huang et al., 2020; Shi et al., 2020a). A clinical study reported the elevated serum cardiac troponin T levels consistent with the clinical manifestations of cardiac dysfunction in patients of coronavirus disease 2019 (COVID-19) (Guo et al., 2020). Concomitant cardiovascular disorders have been observed in 8%–25% of the overall SARS-CoV-2 infected population (Dhakal et al., 2020; Klassen et al., 2020; Nishiga et al., 2020). Recent studies found that SARS-CoV-2 could directly infect cardiomyocytes (CMs) of human myocardium (Bailey et al., 2021) and human pluripotent stem cell-derived CMs (hPSC-CMs) (Bojkova et al., 2020; Perez-Bermejo et al., 2020; Sharma et al., 2020). SARS-CoV-2-infected hPSC-CMs show increased cell death (Bojkova et al., 2020; Sharma et al., 2020), fractionated sarcomeres (Perez-Bermejo et al., 2020), and abnormal electrical and mechanical functions, which phenocopy the myocardium injuries of patients with COVID-19 (Yang et al., 2020). Therefore, hPSC-CMs provide an ideal *in vitro* system for modeling and studying the molecular mechanisms by which SARS-CoV-2 genes induced CM injury and dysfunction, which is critical for the management of heart conditions of patients under acute and post-acute SARS-CoV-2 infection.

It is known that the SARS-CoV-2 genome processes 14 open reading frames (ORFs) and encodes 27 proteins. Currently, individual SARS-CoV-2 gene-induced abnormalities of human heart muscle remain elusive. A recent study analyzed the SARS-CoV-2 protein interaction map in human HEK293 cells (Gordon et al., 2020) to reveal targets for drug repurposing and found that Orf9c, which is a non-structural accessory protein encoded by the SARS-CoV-2 genome, could interact with mitochondrial and innate immune pathway proteins (Gordon et al., 2020). Additionally, Orf9c overexpression in human lung epithelial cells could suppress antiviral responses and impair interferon signaling, antigen processing and presentation,

 © 2022 The Author(s).
This is an open access article under the CC BY license (http://creativecommons.org/licenses/by/4.0/).

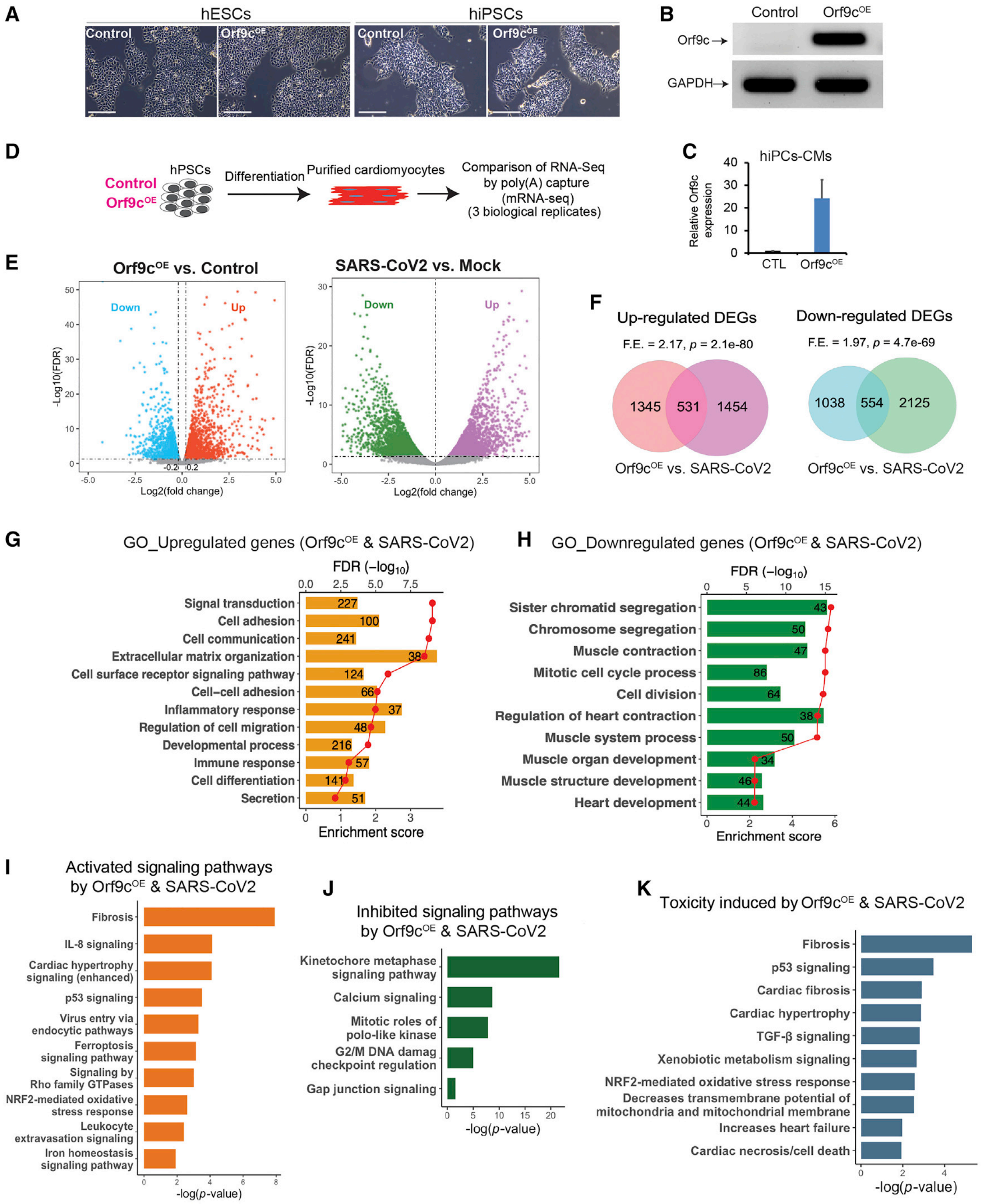

Figure 1. Integrated analyses of mRNA-seq data of hPSC-CMs with Orf9c overexpression and SARS-CoV-2 infection

(A) Stable Orf9c^{OE} hESC/hiPSC cell lines cultured with mTesR medium. Scale bar, 200 μM.

(B) qRT-PCR detection of Orf9c expression level in undifferentiated control and Orf9c^{OE} hESCs.

(C) qRT-PCR detection of Orf9c expression level in control and Orf9c^{OE} hESC-CMs. n = 3.

(D) The scheme of mRNA-seq analysis of Orf9c^{OE} and control hESC-CMs.

(E) Genes with differential expression levels in hESC-CMs (Orf9c^{OE} vs. controls) and hiPSCs (SARS-CoV-2 vs. mock).

(F) Comparison of DE-Gs between hESC-CMs (Orf9c^{OE} vs. Control) and hiPSCs (SARS-CoV-2 vs. Mock). The F.E. around 2 with remarkable *p* value indicates the significant overlap between two gene sets.

(G) GO analysis of concordantly up-regulated genes (Orf9c^{OE} and SARS-CoV-2 infection).

(legend continued on next page)

complement signaling, and induce IL-6 signaling (Andres et al., 2020), implying that Orf9c could mediate the immune evasion of SARS-CoV-2. To date, the response of human CMs to Orf9c remains unknown. Since mitochondria play a key role in CM function and immune response is critical for SARS-CoV-2-induced inflammation of heart, we decided to define the global impacts of Orf9c on human heart muscle cells by overexpressing Orf9c (Orf9c^{OE}) in hPSC-CMs. Genome-wide transcriptomic analysis revealed that both Orf9c^{OE} and SARS-CoV-2 infection of hPSC-CMs activated stress-related cell death, immune and inflammation signaling pathways, and reduced CM functional signaling pathways. Proteomics and interactomic studies uncovered the global impact of Orf9c on proteome of hPSC-CMs and Orf9c-interactive protein network within hPSC-CMs. Orf9c could interact with proteins essential for ATP metabolism and biosynthesis. Further experimental validation confirmed that Orf9c^{OE} induced prominent apoptosis, abnormal calcium handling and electrical properties, and significantly reduced ATP level of hPSC-CMs. Finally, we tested pharmacological approaches to sustain the ATP level of Orf9c^{OE} hPSC-CMs. Notably, two drugs approved by the U.S. Food and Drug Administration (FDA), namely, ivermectin and meclizine, enhanced the ATP level and ameliorated apoptosis and dysfunction of Orf9c^{OE} hPSC-CMs. Overall, we uncovered the global detrimental effects of Orf9c on hPSC-CMs, and explored the alternative usage of FDA-approved drugs for mitigating Orf9c-induced CM injury and dysfunction.

RESULTS

Orf9c^{OE} and SARS-CoV-2 infection caused concordant transcriptomic changes of hiPSC-CMs

To study the impact of Orf9c on the transcriptome of human CMs, both Orf9c^{OE} H9 hESCs and S3 hiPSCs (Carvajal-Vergara et al., 2010) were established by using lentivirus (Figures 1A and 1B). The morphology of Orf9c^{OE} hESC/hiPSC colonies was indistinguishable from that of control colonies (Figure 1A). Orf9c^{OE} slightly reduced OCT4 and SOX2, but not NANOG expression of hiPSCs (Figure S1A). Next, both control and Orf9c^{OE} hESCs were differentiated into CMs, followed with CM enrichment by adding 4 mM lactate into the medium for 4 days (Tohyama et al., 2013). Orf9c was highly expressed in Orf9c^{OE} hESCs and Orf9c^{OE} hESC-CMs (Figures 1B and 1C). We monitored the expression of key mesoderm marker genes T and MESP1 at day 3 and cardiac marker genes PDGFRα, ISL1, and NKX2.5 at day 6 of differentiation. Orf9c^{OE} only slightly reduced expression of ISL1, but not the other genes (Figure S1B), indicating that Orf9c^{OE} did not prominently affect CM differentiation. Next, mRNA sequencing (mRNA-seq) was performed with control GFP^{OE} and Orf9c^{OE} hESC-CMs (Figure 1D) to profile transcriptomic changes (Figure 1E, left). A previously published mRNA-seq dataset from SARS-CoV-2-infected hiPSC-CMs was also analyzed in parallel (Sharma et al., 2020) (Figure 1E, right). Interestingly, both Orf9c^{OE} and SARS-CoV-2 could affect the transcriptome of hPSC-CMs (Figures 1E and 1F, Table S1). Orf9c^{OE} and SARS-CoV-2 up-regulated 1,345 and 1,454 genes, respectively, in addition to 531 genes increased by both (Figure 1F, left), indicating a significant overlap with fold enrichment (F.E.) of 2.17 and a p value of 2.1×10^{-80} between Orf9c^{OE} and SARS-CoV-2. In the meantime, Orf9c^{OE} and SARS-CoV-2 down-regulated 1,592 and 2,679 genes, of which 554 genes were repressed under both conditions, showing the significant ($p = 4.7 \times 10^{-69}$) overlap (F.E. = 1.97) (Figure 1F, right). Gene ontology (GO) analyses of all differentially expressed genes (DE-Gs) found that concordantly up-regulated genes were enriched into biofunctions including signal transduction, cell communication and adhesion, extracellular matrix organization, immune response, and inflammatory response, and so on(Figure 1G), suggesting that Orf9c^{OE} and SARS-CoV-2 could possibly promote cell adhesion via increasing extracellular matrix formation and stimulate inflammation response of host hPSC-CMs. In contrast, the concordantly down-regulated DE-Gs were enriched into muscle contraction, cell division, and muscle and heart development biofunctions (Figure 1H), suggesting that Orf9c^{OE} and SARS-CoV-2 could both compromise CM functions. Furthermore, Orf9c^{OE} and SARS-CoV-2 both activated stress-related signaling pathways including fibrosis, IL-8, p53, Rho, and oxidative stress response (Figure 1I), whereas repressed calcium and gap junction signaling pathways essential for CM function (Figure 1J). Toxicity enrichment analysis found the global detrimental effects of Orf9c and SARS-CoV-2 on hPSC-CMs, focusing on fibrosis, p53, mitochondrial damage, and cardiac cell death (Figure 1K). We then zoomed in the stress-related signaling events. Both Orf9c^{OE} and SARS-CoV-2 infection activated expressions of essential genes involved in apoptosis (Figure 2A), inflammation (Figure 2B), nuclear factor κB (Figure 2C) and extracellular matrix organization (Figure 2D) signaling pathways. Orf9c^{OE} and SARS-CoV-2

(H) GO analysis of concordantly down-regulated genes (Orf9c^{OE} and SARS-CoV-2 infection).
(I) Canonical signaling pathways activated by both Orf9c^{OE} and SARS-CoV-2.
(J) Canonical signaling pathways inhibited by both Orf9c^{OE} and SARS-CoV-2.
(K) Cell toxicities induced by both Orf9c^{OE} and SARS-CoV-2.

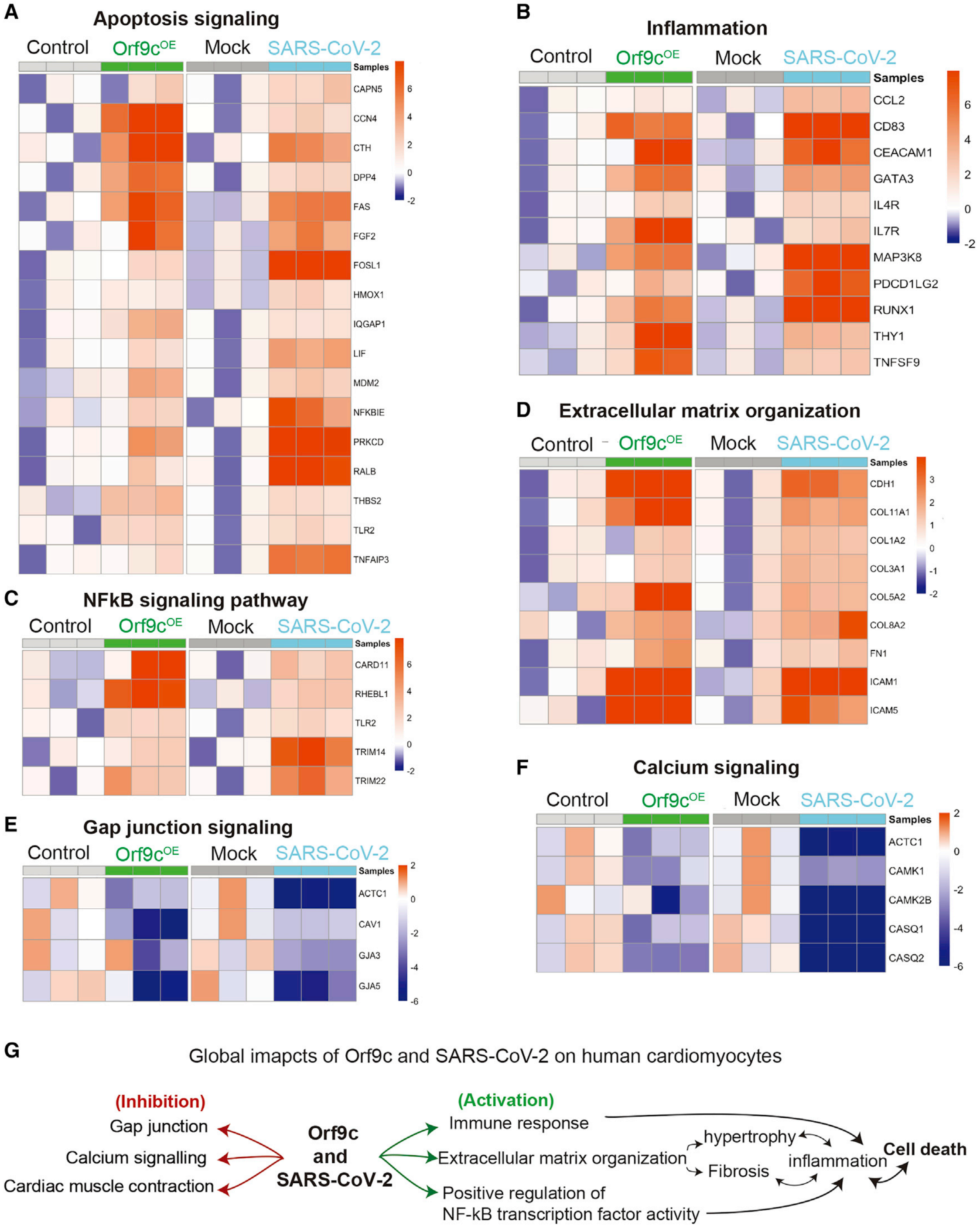

Figure 2. Transcriptomic analyses of the common impacts Orf9c^{OE} and SARS-CoV-2 infection on hPSC-CMs

(A) Heatmap showing apoptosis signaling pathway genes activated by both Orf9c^{OE} and SARS-CoV-2 in hPSC-CMs.

(B–D) Heatmap showing Orf9c^{OE} and SARS-CoV-2 co-activated stress-related signaling pathway genes in inflammation (B), nuclear factor κB (C) and extracellular matrix organization (D) signaling pathways in hPSC-CMs.

(E and F) Orf9c^{OE} and SARS-CoV-2 both reduces expression of genes of gap junction (E) and calcium signaling (F) in hPSC-CMs.

(G) Global view of the Orf9c^{OE} and SARS-CoV-2 infection-induced transcriptomic changes in hPSC-CMs from mRNA-seq data.

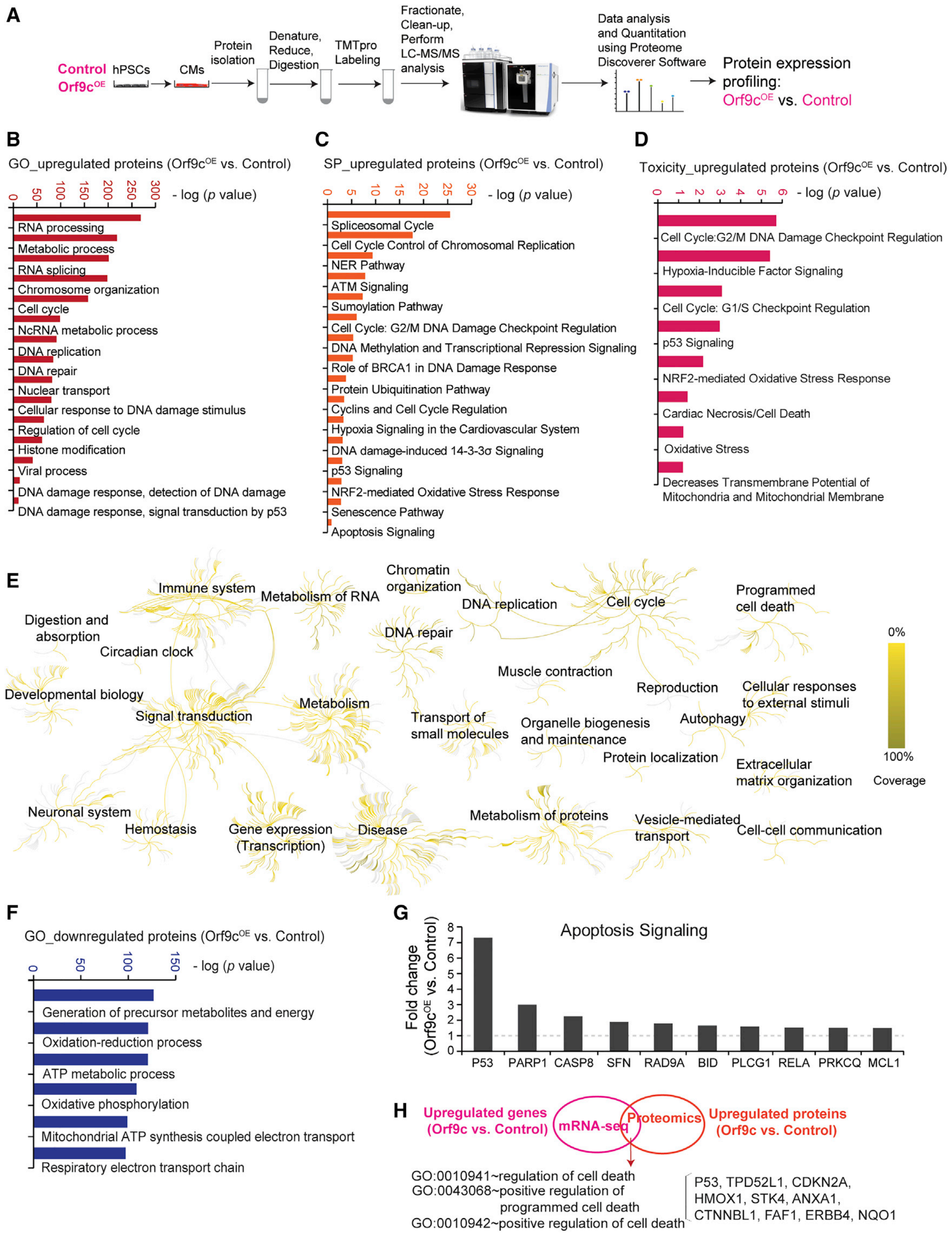

Figure 3. Proteomic analysis of Orf9c^{OE} hESC-CMs

(A) The scheme of TMT-MS analysis of Orf9c^{OE} and control hESC-CMs.
(B) GO analysis of all up-regulated proteins in Orf9c^{OE} vs. control hESC-CMs.
(C) Signaling pathway (SP) analysis of all up-regulated proteins in Orf9c^{OE} vs. control hESC-CMs.
(D) Cell toxicity analysis of all up-regulated proteins (Orf9c^{OE} vs. control).
(E) Reactome pathway analysis of all up-regulated proteins (Orf9c^{OE} vs. Control).

(legend continued on next page)

infection repressed expressions of genes that are key components of the CM gap junction (Figure 2E) and calcium signaling (Figure 2F). Together, Orf9c^{OE} and SARS-CoV-2 infection both globally influenced the transcriptome of hPSC-CMs by activating immune and inflammation responses and cell death signaling pathway, but inhibiting CM calcium and electrical functions (Figure 2G). These concordance analyses suggest that Orf9c could play a key role in SARS-CoV-2 infection-caused CM injuries. For example, Orf9c^{OE} increased mRNA and protein expression levels of P53 in hESC-CMs (Figures S1C–S1E). However, we also noticed that more than 1,000 DE-Gs were only induced by SARS-CoV-2 (Figure 1F), indicating that SARS-CoV-2 genes other than Orf9c could also affect the transcriptome of host hPSC-CMs by targeting other genes.

Orf9c globally influences the proteome of hPSC-CMs

Tandem mass tag-mass spectrometry (TMT-MS) was performed to profile the global protein expression changes in Orf9c^{OE} vs. control hESC-CMs (Figure 3A). A complete list of proteins identified by TMT-MS can be found in Table S2. GO enrichment analysis found that up-regulated proteins (Orf9c^{OE} vs. controls) were over-represented in biofunctions of RNA processing/splicing, metabolism, chromosome/histone organization and DNA damage (Figure 3B), as well as in immune and inflammation responses and cell death (Figures S2A–S2C). Canonical signaling pathway enrichment analysis found that those up-regulated proteins were also involved in spliceosome cycle, cell cycle control, DNA damage regulation, and apoptosis signaling pathways (Figure 3C). Oxidative stress response and cardiac cell death were enriched by up-regulated proteins upon Orf9c^{OE} according to cytotoxicity enrichment analysis (Figure 3D). The reactome pathway analysis provided a global view of key biofunctions that are associated with the up-regulated proteins (Orf9c^{OE} vs. control), including some significant cellular injury events, such as programmed cell death, metabolism, and immune response (Figure 3E). As a comparison, down-regulated proteins (Orf9c^{OE} vs. controls) were enriched into essential cellular energy metabolism events, such as ATP metabolic process and oxidation-reduction process and respiratory electron transport chain (Figures 3F, S2D, and S2E), suggesting that ATP homeostasis of hESC-CMs might be impaired by Orf9c. Notably, TMT-MS data found that the expression levels of many essential apoptotic-related factors were up-regulated in Orf9c^{OE} hESC-CMs compared with control hESC-CMs (Figure 3G). Immunostaining results confirmed the increased protein levels of P53 in Orf9c^{OE} vs. control hESC-CMs (Figures S1D and S1E). Furthermore, by overlapping the Orf9c^{OE}-up-regulated genes from mRNA-seq and TMT-MS, we found that the overlapped genes were condensed into cell death events (Figure 3H). Altogether, unbiased transcriptome- and proteome-wide analyses suggest that Orf9c^{OE} could compromise ATP metabolism and induce apoptosis of hPSC-CMs.

Orf9c overexpression induces apoptosis of hPSC-CMs

Orf9c^{OE} and control hESCs were differentiated into CMs using a 2D monolayer differentiation method (Figure 4A) (Lian et al., 2013). After 12 days of differentiation, flow cytometry was conducted to quantify the ratios of TUNEL^{+} cells in CTNT^{+} hESC-CMs. We found significantly increased ratios of TUNEL^{+} CMs in Orf9c^{OE} hESC-CMs when compared with control hESC-CMs (Figure 4B). Immunostaining of the 2D cultured hESC-CMs also observed increased TUNEL^{+} CMs in Orf9c^{OE} vs. control hESC-CMs (Figures 4C and 4D). Next, beating embryoid bodies (EBs) were differentiated from hiPSCs to mimic 3D heart tissues by using our established method (Lin et al., 2012) (Figure 4E). Similar as in 2D culture, prominently increased ratios of TUNEL^{+} CMs were found in Orf9c^{OE} hiPSC-derived EBs when compared with control hiPSC-EBs by using flow cytometry and immunostaining (Figures 4F–4H). To further confirm the results of TUNEL assay, we quantified the ratios of Annexin V^{+}/PI^{-} CMs and observed increased ratios of Annexin V^{+}/PI^{-} CMs in Orf9c^{OE} hESC-CMs than control hESC-CMs (Figure S3A). Annexin V has a strong affinity for phosphatidylserine residues on the cell surface that is an early marker of apoptosis. We then quantified the ratios of cleaved-CASP3^{+} CMs. Cleaved-CASP3 is an activated form of Caspase 3 responsible for apoptosis execution. Increased percentages of cleaved-CASP3^{+} CMs were found in Orf9c^{OE} hESC-CMs compared with control hESC-CMs, shown by the data from flow cytometry (Figure S3B). Last, the protein levels of apoptotic markers, cleaved-CASP3 and cleaved-CASP9, both increased in Orf9c^{OE} hESC/hiPSC-CMs when compared with control (Figures 4I, 4J, and S3C). Overall, these results demonstrate that Orf9c could induce apoptosis of human heart muscle cells.

Orf9c interactome in hPSC-CMs

In the Orf9c^{OE} and control GFP hPSCs, Orf9c and GFP were each fused with a Strep-tag (Gordon et al., 2020), which allowed us to pull down Orf9c interactive proteins. Therefore, affinity purification was performed in Orf9c^{OE} and

(F) GO analysis of all down-regulated proteins (Orf9c^{OE} vs. Control).

(G) Relative expression levels of proteins related to apoptosis signaling pathway. Fold change was calculated by normalizing Orf9c^{OE} to control. Bars represent the average from duplicates.

(H) GO analysis of up-regulated genes/proteins identified by overlapping mRNA-seq and proteomic data.

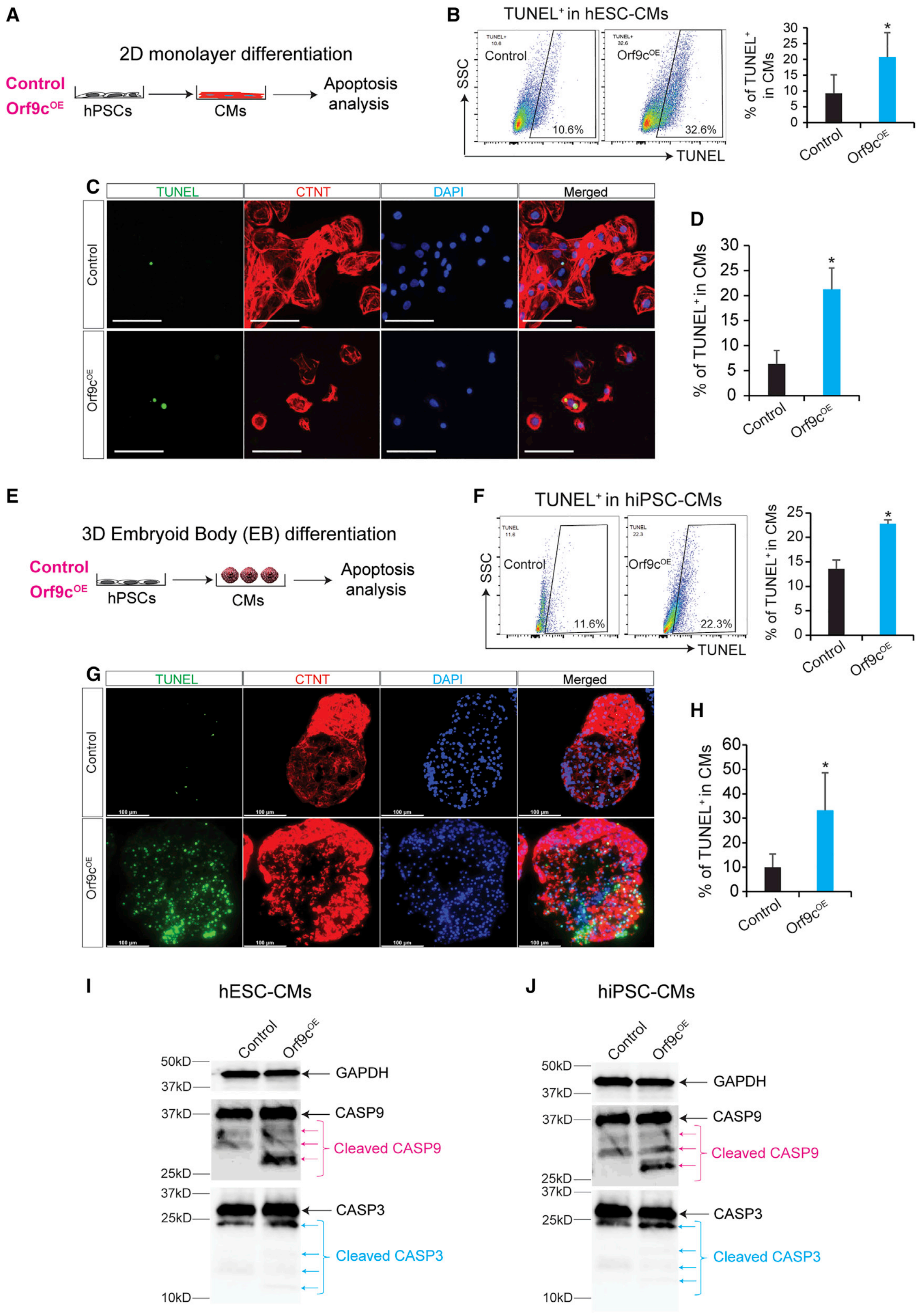

(legend on next page)

control hESC-CMs by using an anti-Strep antibody, followed with mass spectrometry (Co-IP-MS) to capture and identify all Orf9c interacting proteins (Figure 5A). A complete list of Orf9c interactors can be found in Table S3. GO enrichment analysis revealed that Orf9c interactors were involved in important cellular events, including viral process, protein transport/targeting, mRNA splicing, and ATP metabolic process (Figures 5B and S4A). Notably, we conducted protein-protein interaction map analysis as previously described (Gordon et al., 2020) and found that Orf9c interacted with essential protein factors of ATP metabolism and biosynthesis (Figure 5C). Additionally, from the proteomic data, we found that Orf9c^{OE} reduced protein levels of several ATPase subunits in hESC-CMs (Figures 5D and S2D). These results implied that Orf9c could possibly interfere with ATP biosynthesis of hPSC-CMs. Therefore, we quantified the cellular ATP level of hPSC-CMs and found Orf9c^{OE} significantly reduced the ATP level of hESC-CMs/hiPSC-CMs when compared with control CMs (Figures 5E and 5F). These data confirmed that Orf9c^{OE} impaired ATP biosynthesis of hPSC-CMs. Since compromised ATP biosynthesis could lead to cell death (Eguchi et al., 1997; Izyumov et al., 2004; Shiraishi et al., 2001; Skulachev, 2006; Tatsumi et al., 2003), these data also suggested that the enhanced apoptosis of Orf9c^{OE} hESC-CMs could possibly be due to the reduced ATP level.

Orf9c compromises calcium handling and function of hPSC-CMs

Cellular ATP level is critical for the electrophysiology of heart muscle in that insufficient ATP level could impair intracellular Ca^{2+} signaling and excitation-contraction coupling of CMs, which result in reduced contractile capacity (Bers, 2008; Bers and Guo, 2005; Fearnley et al., 2011). Accordingly, we recorded electrically evoked $[Ca^{2+}]_i$ transients in fluo-4-loaded Orf9c^{OE} hESC-CMs using confocal fluorescence microscopy. Representative ⊿F/F0 transients recorded from a control and an Orf9-expressing hiPSC-CM during 0.5 Hz electrical field stimulation are shown in Figure 5G. Peak ⊿F/F0 elevation was smaller and ⊿F/F0 transient duration was shorter in the Orf9c^{OE} CMs. Diastolic ⊿F/F0 returned to pre-stimulation, i.e., resting, levels in the Orf9c^{OE} CMs, but remained elevated in the control CMs. Plots of temporally averaged transients on an expanded timescale (right panels in Figure 5G) revealed distinct differences in ⊿F/F0 amplitude and kinetics. The ⊿F/F0 rise was biphasic in the control CM, with a fast early and a slow late phase, whereas it was monophasic and peaked earlier in the Orf9c^{OE} CM. The ⊿F/F0 recovery was faster and the overall duration of the ⊿F/F0 transient was shorter in the Orf9c^{OE} vs. control CM. Properties of electrically evoked ⊿F/F0 transients are summarized in Figure 5H. Median values for peak ⊿F/F0, 10%–90% rise time, CaT_{30}, CaT_{50}, and CaT_{90} were all significantly reduced in Orf9c^{OE} vs. control CMs. Overall, these results indicate that Orf9c^{OE} altered mechanisms underlying $[Ca^{2+}]_i$ rise and decay of human CMs.

Next, multiple electrodes array (MEA) assays were performed to capture the field potential of spontaneously beating Orf9c^{OE} hPSC-CMs. While control hESC-CMs exhibited normal and rhythmic beating, Orf9c^{OE} hESC-CMs showed increased beating irregularities (Figures 5I and 5J). In MEAs assay, field potential duration (FPD) was used to assess the repolarization of beating CMs (Figure 5K). FPDs of Orf9c^{OE} hESC-CMs were shorter than those of control hESC-CMs (Figure 5L). The same result was observed in Orf9c^{OE} hiPSC-CMs (Figure S4B). Statistically, Orf9c^{OE} significantly reduced the beating rate (Figure 5M), elongated the beat period mean (Figure S4C), and shortened the FPDs (Figure S4D) of hESC-CMs. Spike amplitude, but not spike slope, was reduced by Orf9c^{OE} (Figure S4E). After FPD was adjusted for beating rate by using Bazett's formula, the corrected FPD (FPD_c) value was still significantly decreased in Orf9c^{OE} vs. control hESC-CMs (Figure 5N), suggesting that Orf9c altered the repolarization properties of hESC-CMs. Altogether, our data demonstrate that Orf9c^{OE} could compromise the calcium signaling, automaticity, and repolarization of hPSC-CMs.

Figure 4. Orf9c^{OE} induces apoptosis of hPSC-CMs

(A) HESCs were differentiated into CMs in a 2D monolayer culture, followed by apoptosis analyses.
(B) Flow cytometry analysis of the ratios of TUNEL$^+$ CMs in hESC-CMs.
(C) Representative immunostaining shows TUNEL$^+$ and CTNT$^+$ CMs in hESC-CMs. Scale bar, 100 μm.
(D) Statistical data analysis of immunostaining results of (C).
(E) HiPSCs were differentiated into CMs by forming 3D EBs, followed with apoptosis assays.
(F) Flow cytometry analysis of TUNEL$^+$ CMs in hiPSC-EBs.
(G) Representative immunostaining shows TUNEL$^+$ CMs in hiPSC-EBs. Scale bar, 100 μm.
(H) Statistical analysis of immunostaining results of (G).
(I) Western blotting results of CASP3/9, cleaved-CASP3/9 and GAPDH in hESC-CMs.
(J) Western blotting results of CASP3/9, cleaved-CASP3/9 and GAPDH in hiPSC-CMs. All bars are shown as mean ± SD (n = 3). A two-tailed unpaired *t* test was used to calculate p values: *p < 0.05. n = 3.

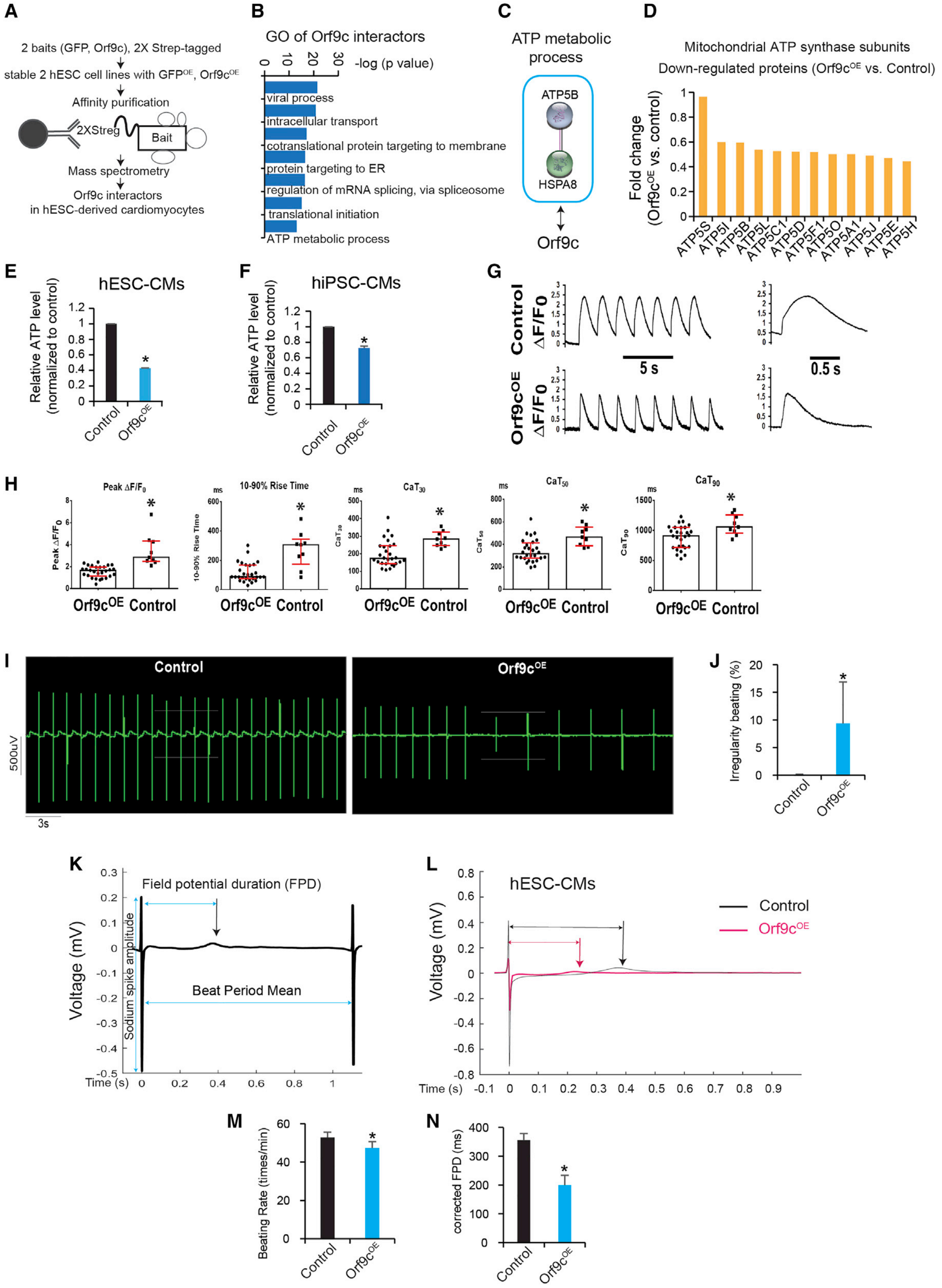

(legend on next page)

FDA-approved drugs ameliorate Orf9c-induced apoptosis and dysfunction of hPSC-CMs

Given the central role of cellular ATP level in viability of various cell types including CMs (Eguchi et al., 1997; Miyoshi et al., 2006; Tatsumi et al., 2003; Tsujimoto, 1997), we posited that enhancing ATP level could rescue Orf9c-induced CM abnormalities. We found two FDA-approved drugs, ivermectin (antiparasitic) and meclizine (antiemetic), were previously reported to protect mitochondrial function and sustain cellular ATP level (Caly et al., 2020; Gohil et al., 2010; Nagai et al., 2017). Interestingly, ivermectin (0.5 μM) or meclizine (0.5 μM) treatment for 3 h could significantly enhance the ATP level of $Orf9c^{OE}$ hESC-CMs (Figure 6A). Importantly, 48 h of ivermectin (0.5 μM) or meclizine (0.5 μM) treatment prominently reduced the ratios of $TUNEL^+$ $Orf9c^{OE}$ hiPSC-CMs when compared with non-treated $Orf9c^{OE}$ hiPSC-CMs (Figures 6B and 6C). These results demonstrate that ivermectin and meclizine could preserve cellular ATP levels to mitigate Of9c-induced apoptosis. Since reduced ATP levels could also impair intracellular Ca^{2+} signaling and excitation-contraction coupling of CMs (Bers, 2008; Bers and Guo, 2005; Fearnley et al., 2011), we next asked whether drug treatment would attenuate $[Ca^{2+}]_i$ abnormality of $Orf9c^{OE}$ hESC-CMs. We found that ivermectin (Figure 6D) (0.5 μM) or meclizine (Figure 6E) (0.5 μM) treatment for 1 h prominently reduced the irregularity of $[Ca^{2+}]_i$ transients of $Orf9c^{OE}$ hESC-CMs. Histogram analysis of the interspike intervals (ISIs) generated from $[Ca^{2+}]_i$ transient recordings showed that drug treatment markedly reduced the variations of ISIs of $Orf9c^{OE}$ hESC-CMs (Figure 6F). Finally, ivermectin and meclizine did not affect the regular beating and FPD of control hESC-CMs (Figures S4F–S4F′), but prolonged the FPD of $Orf9c^{OE}$ hESC-CMs when compared with non-treated hESC-CMs (Figures 6G and 6H). Similar results were observed in ivermectin- or meclizine-treated $Orf9c^{OE}$ hiPSC-CMs (Figure S4G). A statistical analysis of MEA data showed ivermectin increased both FPD and FPD_c, whereas meclizine only increased FPD of $Orf9c^{OE}$ hESC-CMs (Figures 6I and 6J). Collectively, these results demonstrate that the FDA-approved drugs ivermectin and meclizine could ameliorate Orf9c-induced apoptosis and dysfunctions of hPSC-CMs.

DISCUSSION

Understanding the molecular etiology of cardiac disorders of patients with COVID-19 is critical for developing therapies. This study provides insights of the global impacts of Orf9c on transcriptome and proteome of host hPSC-CMs. $Orf9c^{OE}$ in hPSC-CMs led to concordant transcriptomic changes with SARS-CoV-2-infected hPSC-CMs, particularly concentrated on apoptosis, inflammation, and the CM dysfunction signaling pathways. $Orf9c^{OE}$ induced global protein expression changes and interacted with ATPase subunits, which reduced cellular ATP level of hPSC-CMs. Ivermectin and meclizine could restore ATP levels and

Figure 5. $Orf9c^{OE}$ affects ATP biosynthesis, calcium handing and electrical properties of hPSC-CMs

(A) The scheme of co-immunoprecipitation-mass spectrometry (Co-IP MS) to study Orf9c interactors in hESC-CMs.

(B) GO analysis of Orf9c interactors in hESC-CMs.

(C) Orf9c interaction with ATP metabolic process proteins shown by protein-protein interaction map analysis.

(D) Proteomic data of protein expression changes of mitochondrial ATP synthase subunits in $Orf9c^{OE}$ vs. control hESC-CMs. Fold change was calculated by normalizing $Orf9c^{OE}$ to control.

(E) Quantification of cellular ATP level of hESC-CMs. All bars are shown as mean ± SD (n = 3). A two-tailed unpaired *t* test was used to calculate p values: $*p < 0.05$.

(F) Quantification of cellular ATP level of hiPSC-CMs. All bars are shown as mean ± SD (n = 3). A two-tailed unpaired *t* test was used to calculate p values: $*p < 0.05$.

(G) Representative ΔF/F0(t) traces recorded in a control and an $Orf9c^{OE}$ hESC-CM during 0.5 Hz electrical stimulation. Right panels show the average of three consecutive transients of the same cells on the left. $Orf9c^{OE}$ CM exhibits faster upstroke and decay compared with control CM. The increase in $[Ca^{2+}]$i was biphasic in the control CM, with a fast early and a slow late phase.

(H) Summary of $[Ca^{2+}]$i transient properties. Bar height represents median, whereas the whiskers (shown in red) indicate the 25%–75% IQR. Values are from 28 $Orf9c^{OE}$ CMs and nine control CMs. $*p < 0.01$ by Mann-Whitney test (peak ΔF/F0, 10–90% rise time, CaT30) or t test (CaT50, CaT90).

(I) Representative recordings of spontaneous electrical activities of $Orf9c^{OE}$ and control hESC-CMs by using multi-electrode arrays (MEAs).

(J) Statistical results of beating irregularity of MEA data in hESC-CMs.

(K) Scheme of electrical properties by MEAs in hPSC-CMs.

(L) Electrophysiology analysis of hESC-CMs by MEAs. Representative traces illustrate the change of FPD (ms) between $Orf9c^{OE}$ and control hESC-CMs.

(M) Beating rate analysis of MEA data in hESC-CMs.

(N) The FPD_c of MEA data in hESC-CMs. In (J, M, and N), all bars are shown as mean ± SD (n = 4). A two-tailed unpaired *t* test was used to calculate p values. $*p < 0.05$. All bars are shown as mean ± SD.

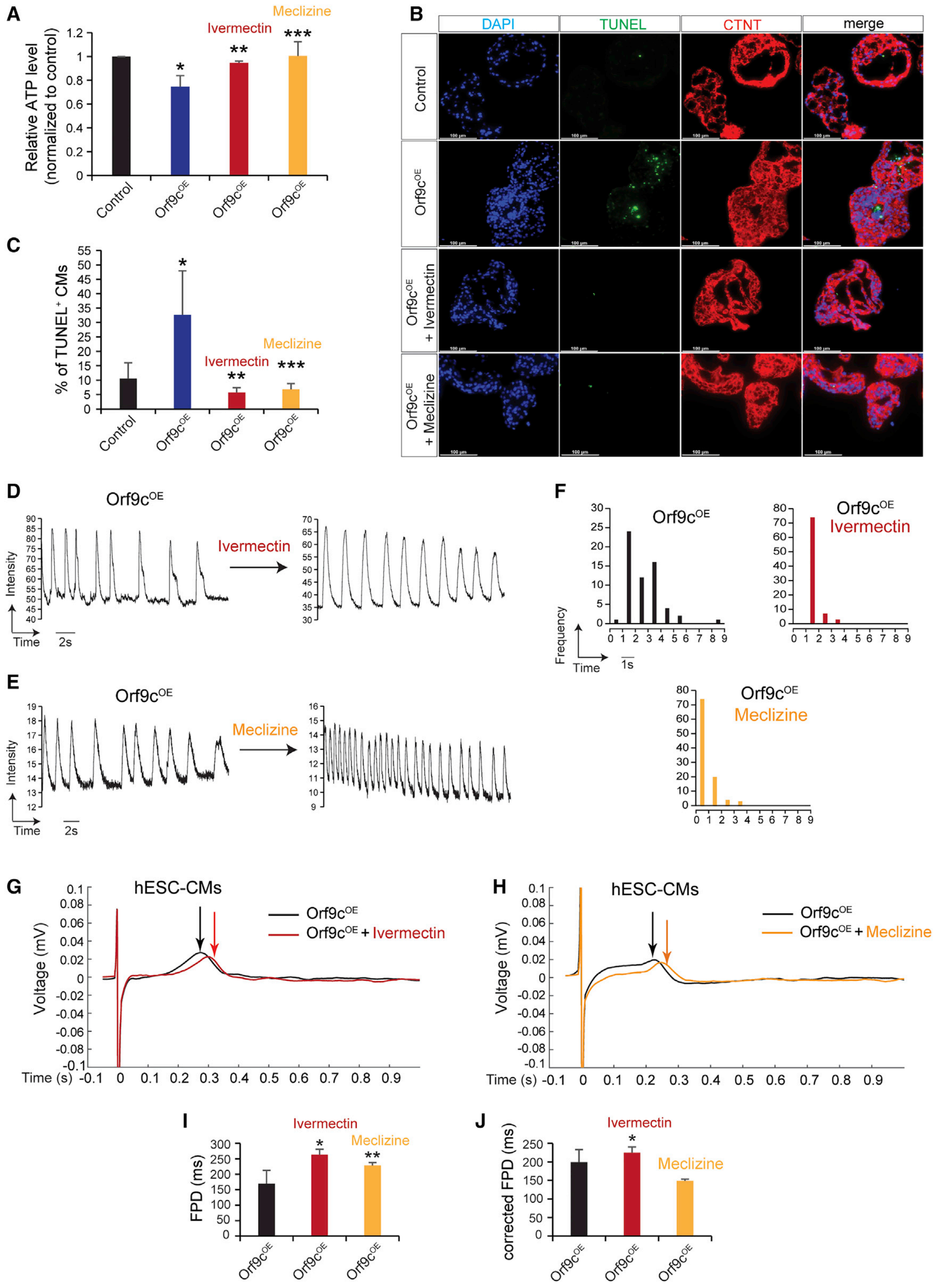

Figure 6. FDA-approved drugs ameliorate cell death and electrical dysfunction of Orf9c^{OE} hPSC-CMs

(A) Quantification of cellular ATP level of hESC-CMs. Final concentration of ivermectin and meclizine was 0.5 μM. Treatment time was 3 h. All bars are shown as mean ± SD (n = 3). A two-tailed unpaired *t* test was used to calculate p values. *p < 0.05 (vs. control), **p < 0.05 (vs. Orf9c^{OE}), ***p < 0.05 (vs. Orf9c^{OE}).

(legend continued on next page)

ameliorate Orf9c^{OE}-induced apoptosis and functional abnormalities of hPSC-CMs. Our findings thus provide mechanistic insights of the detrimental effects of a SARS-CoV-2 gene, Orf9c, on survival and function of human CMs.

COVID-19-associated cardiac manifestations include acute myocardial infarction, myocarditis, inflammatory response, acute heart failure, arrhythmia, hypoxemia, pulmonary embolism, and even stress cardiomyopathy, and multiple cardiac conditions could even coexist in the same patient (Shi et al., 2020b). Recently, Bailey et al. provided evidence that SARS-CoV-2 can directly and specifically infect CMs of patients with COVID-19 (Bailey et al., 2021). CM infection was identified by intramyocyte expressions of viral RNA and protein, and macrophage infiltration associated with areas of myocyte cell death. Although SARS-CoV-2 genome encodes 27 genes (Gordon et al., 2020), most studies were focused on the spike protein. Orf9c is one of the eight accessory proteins encoded by the SARS-CoV-2 genome, and interacts with mitochondrial and innate immune pathway proteins in human HEK293 cells (Gordon et al., 2020). In this study, we observed that Orf9c^{OE} in hPSC-CMs activated mRNA and protein levels of essential genes of immune and inflammation signaling pathways, and Orf9c interacted with ATPase subunits, which were consistent from previous findings from non-CMs. Notably, in hPSC-CMs, Orf9c^{OE} significantly reduced the ATP level and enhanced the protein levels of apoptotic markers, cleaved-CASP3 and cleaved-CASP9. Additionally, Orf9c impaired calcium handling and function of hPSC-CMs, which phenocopied SARS-CoV-2-infected hPSC-CMs showing enhanced apoptosis (Bojkova et al., 2020; Sharma et al., 2020) and electrical dysfunctions (Marchiano et al., 2021). Particularly, both Orf9c^{OE} and SARS-CoV-2 infection showed concordant impacts on transcriptome of hPSC-CMs (Figures 1E and 1F), with 531 elevated genes and 554 repressed genes by both conditions (Figure 1F). Considering the significant p values of 2.1×10^{-80} for up-regulated genes and 4.7×10^{-69} for down-regulated genes between Orf9c^{OE} and SARS-CoV-2, and the fact that those genes with concordant expression changes were concentrated into key signaling pathways for survival, function, and inflammation signaling pathways of human CMs, our results indicate that, of all SARS-CoV-2 viral genes, Orf9c might play a critical role in SARS-CoV-2-induced detrimental effects on human heart muscle cells. Interestingly, the integrated analysis of mRNA-seq datasets from Orf9c^{OE} hPSC-CMs and SARS-CoV-2-infected hPSC-CMs also identified more than 1,000 DEGs solely induced by SARS-CoV-2 but not by Orf9c^{OE} (Figure 1F), suggesting that the other SARS-CoV-2 genes might affect the transcriptome of host human CMs via different mechanisms than Orf9c, which will be further pursued.

An interactome analysis found that Orf9c interacted with ATPase subunits and compromised cellular ATP level of hPSC-CMs. Although the detailed mechanism requires further investigation, our results reveal the central role of ATP homeostasis in Orf9c-induced CM injuries, which is probably also important in SARS-CoV-2-casued tissue injuries of lung, kidney, and blood mononuclear cells (Ajaz et al., 2021). Besides an impaired cellular ATP level of hPSC-CMs, Orf9c^{OE} also led to the up-regulated expressions of proteins for mRNA splicing, and protein translation and transport (Figures 3B and 3C), which were observed in SARS-CoV-2-infected HEK293T cells (Gordon et al., 2020). A protein-protein interaction map analysis found Orf9c interacted with RPS15A, which catalyzes protein synthesis and is associated with viral mRNA translation and activation of the mRNA upon binding of the cap-binding complex and eIFs (Genuth and Barna, 2018; Li, 2019). All these findings suggest that Orf9c could broadly affect host human CMs through various mechanisms.

HPSC-CMs are not identical to mature CMs of adult human heart. Orf9 caused distinct alterations of CM $[Ca^{2+}]_i$ transients, including reduced peak amplitude and

(B) Representative immunostaining of TUNEL^{+} and CTNT^{+} CMs in hiPSC-EBs. Final concentration of ivermectin and meclizine was 0.5 μM. Treatment time was 48 h.
(C) Statistical analysis of immunostaining results of (B).
(D) Calcium imaging of hESC-CMs treated with ivermectin. The y axis shows intensity and the x axis represents time (in seconds, s). Ivermectin final concentration was 0.5 μM. Treatment time was 1 h.
(E) Calcium imaging of hESC-CMs treated with meclizine. The y axis shows intensity and the x axis represents time (in seconds, s). Meclizine final concentration was 0.5 μM. Treatment time was 1 h.
(F) ISI distribution data from (D and E). The y axis means beating frequency in a specific time window. The x axis means time (in seconds, s).
(G and H) Analysis of data collected by multi-electrode arrays (MEAs) in Orf9c^{OE} hESC-CMs treated with ivermectin (G) or meclizine (H). Representative traces illustrate the change of FPD (ms) after treatment. Final concentration of ivermectin and meclizine was 0.5 μM. Treatment time was 2 h.
(I) FPD of hESC-CMs. All bars are shown as mean ± SD (n = 4). A two-tailed unpaired *t* test was used to calculate p values. *p < 0.05 (vs. control), **p < 0.05 (vs. control).
(J) The FPD_c of hESC-CMs. All bars are shown as mean ± SD (n = 4). A two-tailed unpaired *t* test was used to calculate p values. *p < 0.05 (vs. control).

accelerated rise and decay times (Figure 5H). Our proteomic analyses revealed the reduced expressions of SERCA2 and RyR2 in Orf9c^{OE} hPSC-CMs (Table S2), suggesting the possibility that reduced SR Ca^{2+} content and/or density of the SR Ca^{2+} release channels might contribute to Orf9c-induced negative inotropy. In mature CMs, transverse tubular membranes enable the fast radial spread of the action potential, resulting in a spatiotemporally uniform SR Ca^{2+} release throughout the cell. However, it is known that hPSC-CMs typically exhibit an immature phenotype with a poorly developed tubular system (Guo et al., 2020). Thus, the monophasic and abbreviated $[Ca^{2+}]_i$ rise seen in Orf9c^{OE} hPSC-CMs could possibly be due to the altered organization of an immature transverse tubular network compared with that in the control hPSC-CMs. Although reduced expressions of SERCA2 and SLC8A1 were expected to slow diastolic recovery of $[Ca^{2+}]_i$, Orf9c^{OE} accelerated the $[Ca^{2+}]_i$ diastolic recovery of CMs. We can only speculate that this is a combined effect by Orf9c^{OE} to dysregulate the compensatory mechanisms to effectively offset the decreased SERCA2 and SLC8A1 in immature hPSC-CMs.

Ivermectin is one of a series of 16-membered macrocyclic lactone compounds discovered in 1967, and received FDA approval in 1987 for treating parasitic infections. Ivermectin was also found to enhance ATP production in the mitochondria of HL-1 CMs by up-regulating the transcription of Cox6a2, a subunit of the mitochondrial respiratory chain (Nagai et al., 2017). In this study, we observed that Orf9c^{OE} in hPSC-CMs down-regulated expressions of protein factors essential for mitochondrial ATP biosynthesis (Figure S2D) and respiratory electron transport chain, including multiple COX5/6/7 genes (Figure S2E), suggesting that ivermectin might mitigate apoptosis and dysfunction of Orf9c^{OE} hPSC-CMs at the transcription level. Additionally, a recent study reported that ivermectin could inhibit the replication of SARS-CoV-2 virus *in vitro* (Caly et al., 2020). However, a recent clinic study including 476 adult patients with COVID-19 with mild disease and symptoms found that a 5-day course of ivermectin did not significantly improve the time to resolution of symptoms when compared with placebo (Lopez-Medina et al., 2021). Although these findings suggested that ivermectin was not generally efficacious for treatment of patients with mild COVID-19 (Lopez-Medina et al., 2021), whether ivermectin could be used alone or in combination with other drugs for treating cardiac manifestations remains unknown, and more randomized controlled trials are required.

Meclizine is primarily used as an antihistamine. However, a nutrient-sensitized screening identified a cardioprotection effect of meclizine through promoting CM glycolysis to increase ATP synthesis (Gohil et al., 2010). Meclizine could stimulate glycolysis, mitigate ATP depletion, and protect mitochondrial function (Zhuo et al., 2016). These studies support our findings and can, at least in part, explain why meclizine could increase the ATP level to mitigate apoptosis of Orf9c^{OE} hPSC-CMs. Overall, our results indicate that maintaining ATP balance could possibly be a central target for the therapy of COVID-19-associated heart injuries and abnormalities.

EXPERIMENTAL PROCEDURES

Detailed methods are provided in the supplemental experimental procedures.

Human pluripotent stem cells culture and CM differentiation and purification

Human embryonic stem cell (hESC) line H9 and human induced pluripotent stem cell (hiPSC) line S3 were cultured on Matrigel (BD Biosciences)-coated plates in mTesR1 medium (Ludwig et al., 2006a, 2006b). For CM differentiation, the monolayer differentiation method was used based on a published protocol (Lian et al., 2013). After 14 days of differentiation, DMEM medium (no glucose) with 4 mM lactate (Sigma, #CAT-L1750-10G) was used to enrich CMs according to the published protocol (Tohyama et al., 2013). For EBs differentiation (3D differentiation), hPSCs were differentiated toward CMs using the previously established protocol (Lin et al., 2012; Yang et al., 2008). For drug treatment assay, all beating EBs were maintained in DMEM medium (no glucose, Gibco) with 10% FBS. The final concentration of ivermectin or meclizine was 0.5 μM (drugs were dissolved in DMSO). The drug treatment time for apoptosis assay was 2 days. All cytokines were from R&D Systems. All chemicals and drugs were from Sigma Aldrich.

Lentivirus production and cell transduction

The lentiviral vector pLVX-EF1alpha-IRES-Puro-2xStrep-GFP or pLVX-EF1alpha-IRES-Puro-2xStrep-Orf9c (Addgene plasmid #141395 and #141393) was transfected into the HEK293T cells with packaging plasmids psPAX2 and pMD2.G using X-treme GENE nine transfection reagent (Roche). After 48 h, viral supernatant was collected and cellular debris was removed by syringe filtering (0.45 μm pore size; Millipore). H9 hESCs or S3 hiPSCs were incubated with virus supernatant (20% vol:vol) for 4 h and same infection was repeated after 24 h. Puromycin was added to select puromycin-resistant H9 hESCs or S3 hiPSCs clones after 48 h of viral infection.

Whole mRNA-seq and proteomic experiment and data analysis

The mRNA-seq were conducted by Indiana University Genomic Core, and the mass spectrometry (MS) analysis, bioinformatics and data evaluation were performed in the Proteomics Core Facility at the Indiana University School of Medicine. Details are provided in the Supplemental information.

Protein-protein interaction analysis

Protein-protein interaction networks analysis was carried out with the STRING software (https://string-db.org/).

Quantification and statistical analysis

Data comparisons between two groups (gene overexpression versus control) were conducted using an unpaired two-tailed *t* test. All data were presented as mean ± SD from at least three independent experiments. Differences with *p* values of less than 0.05 were considered significant. The enrichment of overlap between two sets of genes, e.g., DE-Gs from Orf9c^{OE} and SARS-CoV-2 infection as shown in Figure 1F, was evaluated by the F.E., defined as the ratio of number of observed overlapping genes to number of expected overlaps by the random selections. The statistical significance was calculated by hypergeometric model.

Data and code availability

The accession number for the mRNA-seq dataset reported in this paper is GSE171370.

SUPPLEMENTAL INFORMATION

Supplemental information can be found online at https://doi.org/10.1016/j.stemcr.2022.01.014.

AUTHOR CONTRIBUTIONS

J.L. and L.Y. initiated and designed studies. J.L. performed most of experiments and data analyses. L.H., S.W., C.W., and H.C. assisted in CM differentiation, IHC, and RT-PCR. E.H.D. supported proteomics. Y.Z. and J.W. assisted in bioinformatics analyses. S.G. and M.L. supported calcium handling and MEA experiments and data interpretation. J.L., J.W., M.L., and L.Y. wrote and revised the manuscript.

CONFLICTS OF INTEREST

The authors declare that they have no conflict of interest.

ACKNOWLEDGMENTS

The authors thank the Proteomics Core of Indiana University School of Medicine (IUSM). Work in the IUSM Proteomics Core was supported, in part, with support from the Indiana Clinical and Translational Sciences Institute, which is funded by NIH UL1TR002529 from the National Center for Advancing Translational Sciences, Clinical and Translational Sciences Award. Acquisition of the IUSM Proteomics core instrumentation used for this project, the Orbitrap Fusion Lumos, was provided by the Indiana University Precision Health Initiative. We thank Indiana Center for Biological Microscopy of IUSM for cell imaging.

Supported by Startup, Showalter Scholar Award and American Heart Association Established Investigator Award (20EIA35260114) to L.Y.

Received: April 19, 2021
Revised: January 17, 2022
Accepted: January 18, 2022
Published: February 17, 2022

REFERENCES

Ajaz, S., McPhail, M.J., Singh, K.K., Mujib, S., Trovato, F.M., Napoli, S., and Agarwal, K. (2021). Mitochondrial metabolic manipulation by SARS-CoV-2 in peripheral blood mononuclear cells of patients with COVID-19. Am. J. Physiol. Cell Physiol. *320*, C57–C65.

Andres, A.D., Feng, Y., Campos, A.R., Yin, J., Yang, C.C., James, B., Murad, R., Kim, H., Deshpande, A.J., Gordon, D.E., et al. (2020). SARS-CoV-2 ORF9c is a membrane-associated protein that suppresses antiviral responses in cells. bioRxiv https://doi.org/10.1101/2020.08.18.256776.

Bailey, A.L., Dmytrenko, O., Greenberg, L., Bredemeyer, A.L., Ma, P., Liu, J., Penna, V., Winkler, E.S., Sviben, S., Brooks, E., et al. (2021). SARS-CoV-2 infects human engineered heart tissues and models COVID-19 myocarditis. JACC Basic Transl. Sci. *6*, 331–345.

Bers, D.M. (2008). Calcium cycling and signaling in cardiac myocytes. Annu. Rev. Physiol. *70*, 23–49.

Bers, D.M., and Guo, T. (2005). Calcium signaling in cardiac ventricular myocytes. Ann. N. Y. Acad. Sci. *1047*, 86–98.

Bojkova, D., Wagner, J.U.G., Shumliakivska, M., Aslan, G.S., Saleem, U., Hansen, A., Luxan, G., Gunther, S., Pham, M.D., Krishnan, J., et al. (2020). SARS-CoV-2 infects and induces cytotoxic effects in human cardiomyocytes. Cardiovasc. Res. *116*, 2207–2215.

Caly, L., Druce, J.D., Catton, M.G., Jans, D.A., and Wagstaff, K.M. (2020). The FDA-approved drug ivermectin inhibits the replication of SARS-CoV-2 in vitro. Antivir. Res. *178*, 104787.

Carvajal-Vergara, X., Sevilla, A., D'Souza, S.L., Ang, Y.S., Schaniel, C., Lee, D.F., Yang, L., Kaplan, A.D., Adler, E.D., Rozov, R., et al. (2010). Patient-specific induced pluripotent stem-cell-derived models of LEOPARD syndrome. Nature *465*, 808–812.

Cheng, Y., Luo, R., Wang, K., Zhang, M., Wang, Z., Dong, L., Li, J., Yao, Y., Ge, S., and Xu, G. (2020). Kidney disease is associated with in-hospital death of patients with COVID-19. Kidney Int. *97*, 829–838.

Dhakal, B.P., Sweitzer, N.K., Indik, J.H., Acharya, D., and William, P. (2020). SARS-CoV-2 infection and cardiovascular disease: COVID-19 heart. Heart Lung Circ. *29*, 973–987.

Eguchi, Y., Shimizu, S., and Tsujimoto, Y. (1997). Intracellular ATP levels determine cell death fate by apoptosis or necrosis. Cancer Res. *57*, 1835–1840.

Fearnley, C.J., Roderick, H.L., and Bootman, M.D. (2011). Calcium signaling in cardiac myocytes. Cold Spring Harb. Perspect. Biol. *3*, a004242.

Genuth, N.R., and Barna, M. (2018). Heterogeneity and specialized functions of translation machinery: from genes to organisms. Nat. Rev. Genet. *19*, 431–452.

Gohil, V.M., Sheth, S.A., Nilsson, R., Wojtovich, A.P., Lee, J.H., Perocchi, F., Chen, W., Clish, C.B., Ayata, C., Brookes, P.S., and Mootha, V.K. (2010). Nutrient-sensitized screening for drugs that shift energy metabolism from mitochondrial respiration to glycolysis. Nat. Biotechnol. *28*, 249–255.

Gordon, D.E., Jang, G.M., Bouhaddou, M., Xu, J., Obernier, K., White, K.M., O'Meara, M.J., Rezelj, V.V., Guo, J.Z., Swaney, D.L.,

et al. (2020). A SARS-CoV-2 protein interaction map reveals targets for drug repurposing. Nature *583*, 459–468.

Guo, T., Fan, Y., Chen, M., Wu, X., Zhang, L., He, T., Wang, H., Wan, J., Wang, X., and Lu, Z. (2020). Cardiovascular implications of fatal outcomes of patients with coronavirus disease 2019 (COVID-19). JAMA Cardiol. *5*, 811–818.

Hikmet, F., Mear, L., Edvinsson, A., Micke, P., Uhlen, M., and Lindskog, C. (2020). The protein expression profile of ACE2 in human tissues. Mol. Syst. Biol. *16*, e9610.

Hoffmann, M., Kleine-Weber, H., Schroeder, S., Kruger, N., Herrler, T., Erichsen, S., Schiergens, T.S., Herrler, G., Wu, N.H., Nitsche, A., et al. (2020). SARS-CoV-2 cell entry depends on ACE2 and TMPRSS2 and is blocked by a clinically proven protease inhibitor. Cell *181*, 271–280 e278.

Huang, C., Wang, Y., Li, X., Ren, L., Zhao, J., Hu, Y., Zhang, L., Fan, G., Xu, J., Gu, X., et al. (2020). Clinical features of patients infected with 2019 novel coronavirus in Wuhan, China. Lancet *395*, 497–506.

Izyumov, D.S., Avetisyan, A.V., Pletjushkina, O.Y., Sakharov, D.V., Wirtz, K.W., Chernyak, B.V., and Skulachev, V.P. (2004). "Wages of fear": transient threefold decrease in intracellular ATP level imposes apoptosis. Biochim. Biophys. Acta *1658*, 141–147.

Klassen, S.L., Kwan, G.F., and Bukhman, G. (2020). The COVID-19 pandemic A massive threat for those living with cardiovascular disease among the poorest billion. Circulation *142*, 1887–1889.

Li, S. (2019). Regulation of ribosomal proteins on viral infection. Cells *8*, 508.

Lian, X., Zhang, J., Azarin, S.M., Zhu, K., Hazeltine, L.B., Bao, X., Hsiao, C., Kamp, T.J., and Palecek, S.P. (2013). Directed cardiomyocyte differentiation from human pluripotent stem cells by modulating Wnt/beta-catenin signaling under fully defined conditions. Nat. Protoc. *8*, 162–175.

Lin, B., Kim, J., Li, Y., Pan, H., Carvajal-Vergara, X., Salama, G., Cheng, T., Li, Y., Lo, C.W., and Yang, L. (2012). High-purity enrichment of functional cardiovascular cells from human iPS cells. Cardiovasc. Res. *95*, 327–335.

Lopez-Medina, E., Lopez, P., Hurtado, I.C., Davalos, D.M., Ramirez, O., Martinez, E., Diazgranados, J.A., Onate, J.M., Chavarriaga, H., Herrera, S., et al. (2021). Effect of ivermectin on time to resolution of symptoms among adults with mild COVID-19 A randomized clinical trial. JAMA *325*, 1426–1435.

Ludwig, T.E., Bergendahl, V., Levenstein, M.E., Yu, J., Probasco, M.D., and Thomson, J.A. (2006a). Feeder-independent culture of human embryonic stem cells. Nat. Methods *3*, 637–646.

Ludwig, T.E., Levenstein, M.E., Jones, J.M., Berggren, W.T., Mitchen, E.R., Frane, J.L., Crandall, L.J., Daigh, C.A., Conard, K.R., Piekarczyk, M.S., et al. (2006b). Derivation of human embryonic stem cells in defined conditions. Nat. Biotechnol. *24*, 185–187.

Marchiano, S., Hsiang, T.Y., Khanna, A., Higashi, T., Whitmore, L.S., Bargehr, J., Davaapil, H., Chang, J., Smith, E., Ong, L.P., et al. (2021). SARS-CoV-2 infects human pluripotent stem cell-derived cardiomyocytes, impairing electrical and mechanical function. Stem Cell Rep. *16*, 478–492.

Miyoshi, N., Oubrahim, H., Chock, P.B., and Stadtman, E.R. (2006). Age-dependent cell death and the role of ATP in hydrogen peroxide-induced apoptosis and necrosis. Proc. Natl. Acad. Sci. U S A *103*, 1727–1731.

Nagai, H., Satomi, T., Abiru, A., Miyamoto, K., Nagasawa, K., Maruyama, M., Yamamoto, S., Kikuchi, K., Fuse, H., Noda, M., and Tsujihata, Y. (2017). Antihypertrophic effects of small molecules that maintain mitochondrial ATP levels under hypoxia. Ebiomedicine *24*, 147–158.

Nishiga, M., Wang, D.W., Han, Y., Lewis, D.B., and Wu, J.C. (2020). COVID-19 and cardiovascular disease: from basic mechanisms to clinical perspectives. Nat. Rev. Cardiol. *17*, 543–558.

Perez-Bermejo, J.A., Kang, S., Rockwood, S.J., Simoneau, C.R., Joy, D.A., Ramadoss, G.N., Silva, A.C., Flanigan, W.R., Li, H., Nakamura, K., et al. (2020). SARS-CoV-2 infection of human iPSC-derived cardiac cells predicts novel cytopathic features in hearts of COVID-19 patients. Sci. Transl. Med. *590*, eabf7872.

Sharma, A., Garcia, G., Wang, Y.Z., Plummer, J.T., Morizono, K., Arumugaswami, V., and Svendsen, C.N. (2020). Human iPSC-derived cardiomyocytes are susceptible to SARS-CoV-2 infection. Cell Rep. Med. *1*, 100052.

Shi, S., Qin, M., Shen, B., Cai, Y., Liu, T., Yang, F., Gong, W., Liu, X., Liang, J., Zhao, Q., et al. (2020a). Association of cardiac injury with mortality in hospitalized patients with COVID-19 in Wuhan, China. JAMA Cardiol. *5*, 802–810.

Shi, S., Qin, M., and Yang, B. (2020b). Coronavirus disease 2019 (COVID-19) and cardiac injury-reply. JAMA Cardiol. *7*, 802–810.

Shiraishi, J., Tatsumi, T., Keira, N., Akashi, K., Mano, A., Yamanaka, S., Matoba, S., Asayama, J., Yaoi, T., Fushiki, S., et al. (2001). Important role of energy-dependent mitochondrial pathways in cultured rat cardiac myocyte apoptosis. Am. J. Physiol. Heart Circ. Physiol. *281*, H1637–H1647.

Skulachev, V.P. (2006). Bioenergetic aspects of apoptosis, necrosis and mitoptosis. Apoptosis *11*, 473–485.

Tatsumi, T., Shiraishi, J., Keira, N., Akashi, K., Mano, A., Yamanaka, S., Matoba, S., Fushiki, S., Fliss, H., and Nakagawa, M. (2003). Intracellular ATP is required for mitochondrial apoptotic pathways in isolated hypoxic rat cardiac myocytes. Cardiovasc. Res. *59*, 428–440.

Tohyama, S., Hattori, F., Sano, M., Hishiki, T., Nagahata, Y., Matsuura, T., Hashimoto, H., Suzuki, T., Yamashita, H., Satoh, Y., et al. (2013). Distinct metabolic flow enables large-scale purification of mouse and human pluripotent stem cell-derived cardiomyocytes. Cell Stem Cell *12*, 127–137.

Tsujimoto, Y. (1997). Apoptosis and necrosis: intracellular ATP level as a determinant for cell death modes. Cell Death Differ. *4*, 429–434.

Yang, L., Han, Y., Nilsson-Payant, B.E., Gupta, V., Wang, P., Duan, X., Tang, X., Zhu, J., Zhao, Z., Jaffre, F., et al. (2020). A human pluripotent stem cell-based platform to study SARS-CoV-2 tropism and model virus infection in human cells and organoids. Cell Stem Cell *27*, 125–136.e7.

Yang, L., Soonpaa, M.H., Adler, E.D., Roepke, T.K., Kattman, S.J., Kennedy, M., Henckaerts, E., Bonham, K., Abbott, G.W., Linden,

R.M., et al. (2008). Human cardiovascular progenitor cells develop from a KDR+ embryonic-stem-cell-derived population. Nature *453*, 524–528.

Zang, R., Gomez Castro, M.F., McCune, B.T., Zeng, Q., Rothlauf, P.W., Sonnek, N.M., Liu, Z., Brulois, K.F., Wang, X., Greenberg, H.B., et al. (2020). TMPRSS2 and TMPRSS4 promote SARS-CoV-2 infection of human small intestinal enterocytes. Sci. Immunol. *47*, eabc3582.

Zhu, N., Zhang, D., Wang, W., Li, X., Yang, B., Song, J., Zhao, X., Huang, B., Shi, W., Lu, R., et al. (2020). A novel coronavirus from patients with pneumonia in China, 2019. N. Engl. J. Med. *382*, 727–733.

Zhuo, M., Gorgun, M.F., and Englander, E.W. (2016). Augmentation of glycolytic metabolism by meclizine is indispensable for protection of dorsal root ganglion neurons from hypoxia-induced mitochondrial compromise. Free Radic. Biol. Med. *99*, 20–31.

Stem Cell Reports, Volume *17*

Supplemental Information

Genome-wide analyses reveal the detrimental impacts of SARS-CoV-2 viral gene Orf9c on human pluripotent stem cell-derived cardiomyocytes

Juli Liu, Yucheng Zhang, Lei Han, Shuai Guo, Shiyong Wu, Emma Helen Doud, Cheng Wang, Hanying Chen, Michael Rubart-von der Lohe, Jun Wan, and Lei Yang

Supplemental experimental procedures

RT-qPCR

RNAs were isolated by RNeasy Kit (Qiagen, USA). CDNA synthesis was carried out using High-Capacity RNA-to-cDNA™ Kit (Applied Biosystems). RT-qPCR was performed on the QuantStudio 6 Real-Time PCR Systems with Fast SYBR Green Master Mix (Applied Biosystems, USA) according to the manufacturer's instructions. The RT-qPCR analysis were normalized to internal control GAPDH or beta-ACTIN using $2^{-\Delta\Delta Ct}$ method (Peltier and Latham, 2008). RT-qPCR data were presented with mean ± S.D. from at least three independent experiments. For all primer information, please see the Table S4 Oligonucleotides.

Western blotting

Proteins were extracted by a Complete™ Lysis-M EDTA-free kit (Roche, 04719964001, USA). Protein samples were mixed with NuPAGE™ LDS Sample Buffer (4X), boiled for 5 min at 95 °C, run on 4–15% Mini-PROTEAN TGX Gels (Bio-Rad, USA), transferred to PVDF membrane by using Trans-Blot® Turbo™ Transfer System (Bio-Rad, USA), blocked with 1X TBST buffer containing 10% not-fat milk, and incubated overnight at 4 °C with the corresponding primary antibodies with 5% BSA in 1X TBST buffer. Membranes were washed three times X 5 mins each with 1X TBXT buffer, and then incubated for 1 h at room temperature with horseradish-peroxidase-conjugated secondary antibodies in blocking solution with 5% BSA in 1X TBST buffer. Membranes were washed again three

times X 5 mins with 1X TBST, loaded with ECL Western Blotting Substrate (Pierce, USA), and imaged using a ChemiDoc Imaging System (Bio-Rad, USA).

Flow cytometry

Flow cytometry was performed as previously described (Lu et al., 2013). Briefly, CMs were harvested and dissociated by using 0.25% trypsin-EDTA at 37°C incubator for 10 mins. The dissociated cells were fixed in 4% PFA (diluted with 16% Paraformaldehyde (formaldehyde) aqueous solution) at room temperature for 10 mins and washed 3 times with 1X PBS. CMs were incubated in 1X blocking PBS buffer (containing 2% goat serum or 5% BSA plus 0.1% saponin) with corresponding primary antibodies at 37°C for 1h, following with corresponding secondary antibodies staining at 37°C for 1 hr. For TUNEL (Terminal deoxynucleotidyl transferase dUTP nick end labeling) experiments, staining was carried out by using an In Situ Cell Death Detection Kit, Fluorescein (11684795910 Roche, USA) according to the manual. Flow cytometry analysis was performed on an Attune NxT Flow Cytometer (Thermo Fisher Scientific, USA). Data were analyzed by using the FlowJo software (Treestar, USA).

Whole mRNA-seq

Total RNAs were extracted by using the RNeasy Kit (Qiagen, USA), followed with QC by using an Agilent Bioanalyzer 2100. One hundred nanograms of total RNAs were used for cDNA library preparation includes mRNA purification/enrichment, RNA fragmentation, cDNA synthesis, ligation of index adaptors and amplification, by following the KAPA

mRNA Hyper Prep Kit Technical Data Sheet, KR1352-v4.17 (Roche Corporate). Each resulting indexed library was quantified and its quality was accessed by Qubit and Agilent Bioanalyzer, and multiple libraries were pooled in equal molarity. The pooled libraries were then denatured and neutralized before being loaded to a NovaSeq 6000 sequencer at 300pM final concentration for 100b paired-end sequencing (Illumina, Inc.). Approximately 40-60M reads per library were generated, more than 90% of which reached a high Phred quality score, Q30 (corresponding to 99.9% base call accuracy). The mRNA-seq data could be accessed via the GEO number GSE171370.

mRNA-seq Data analyses

Quality control for raw mRNA-seq data was generated by FastQC v0.11.5 (http://www.bioinformatics.babraham.ac.uk/projects/fastqc/). Illumina adapter sequences and low-quality bases were trimmed by Trim Galore v0.4.5 (http://www.bioinformatics.babraham.ac.uk/projects/trim_galore/), followed by sequence mapping of high-quality paired-end reads to human genome (hg38) with the aligner STAR v2.7.2b (Dobin et al., 2013). We further used bam-filter in ngsutilsj v0.4.8 (https://compgen.io/ngsutilsj) to keep only properly and uniquely mapped paired reads (MAPQ ≥ 10) for downstream analysis. FeatureCounts from package subread v1.6.5 (Liao et al., 2019) was employed for quantification of gene expression by summarizing mapped reads to corresponding genes according to GENECODE v31 annotation. Analysis of differential expression genes (DEGs) was performed by edgeR v3.32.1(Robinson et al., 2010), with read counts normalized by the trimmed mean of M-values (TMM) method after lowly expressed genes filtered out by the filterByExpr function using default settings. DEGs due to Orf9c^{OE} were

identified if their FDR-adjusted *p*-values were less than 0.05 and amplitudes of fold changes (FCs) in base-2 log scale were larger than 0.2 given the comparison between the Orf9c^{OE} and the control. The gene expression profile of SARS-CoV-2-infected hiPSC-CMs (GSE150392) were achieved from NCBI GEO database (https://www.ncbi.nlm.nih.gov/geo/). The dataset includes raw read counts on genes from three hiPSC-CM samples infected with SARS-CoV-2 and three mock control hiPSC-CMs without virus infection (Sharma et al., 2020). DEG analysis was conducted by using edgeR with the same parameters used for the Orf9c^{OE} study. The cutoff of DEGs were set as FDR-adjusted *p*-values less than 0.05.

Co-immunoprecipitation mass spectrometry (Co-IP MS)

Beads were submitted to the IU Proteomics Core where they were covered in 8 M Urea, 50 mM Tris-HCl, pH 8.5, reduced with 5 mM tris (2-carboxyethyl) phosphine hydrochloride (TCEP) at room temperature for 30 mins and alkylated with 10 mM chloroacetamide (CAM) for 30 mins in dark at room temperature. Digestion was carried out using Trypsin/Lys-C Mass spec grade protease mix (Promega, V5072) at a 1:100 protease to substrate ratio, overnight at 37 °C. The reaction was quenched with 0.5 % formic acid prior to LC-MS. Samples were analyzed using a 5 cm trap column and 15 cm (2 μm particle size, 50 μm diameter) EasySpray (801A) column on an UltiMate 3000 HPLC and Q-Exactive Plus mass spectrometer (Thermo Fisher Scientific). Solvent B was increased from 5%-28% over 155 mins, to 35% over 5 mins, to 65% over 10 mins and back to 5% over 12 mins (Solvent A: 95% water, 5% acetonitrile, 0.1% formic acid; Solvent B: 100% acetonitrile, 0.1% formic acid). A data dependent top 20 method acquisition method was

used with MS scan range of 350-1600 m/z, resolution of 70,000, AGC target 3e6, maximum IT of 50 ms. MS2 settings of fixed first mass 100 m/z, normalized collision energy of 36, isolation window of 1.5 m/z, resolution of 35,000, target AGC of 1e5, and maximum IT of 250 ms. For dd acquisition, a minimum AGC of 2e3 and charge exclusion of 1, and ⩾7 were used.

Tandem Mass Tag-mass spectrometry (TMT-MS)

Cell and tissue preparation. Cells were lysed in 8 M urea, 50 mM Tris-HCl, pH 8.5. Samples were sonicated in a Bioruptor® sonication system from Diagende Inc. (30 sec/30 sec on/off cycles for 15 mins, 4 °C). Following centrifugation at 12,000 rpm for 15 mins, protein concentrations were determined using a Bradford protein assay (cat. num. 5000002, Bio-Rad). Protein samples in equal amounts (30 μg) were reduced with 5 mM TCEP and alkylated with 10 mM (CAM). Samples were diluted with 100 mM Tris-HCl to a final urea concentration of 2 M and digested overnight with Trypsin/Lys-C Mix Mass Spectrometry (1:100 protease: substrate ratio, cat. num. V5072, Promega) (Levasseur et al., 2019; Peck Justice et al., 2020; Plubell et al., 2017).

Peptide purification and labeling. Peptides were desalted on 50 mg Sep-Pak® Vac (Waters Corporation) employing a vacuum manifold. After elution from the column in 70% acetonitrile (ACN) 0.1% formic acid (FA), peptides were dried by speed vacuum and resuspended in 24 μL of 50 mM triethylammonium bicarbonate (TEAB). Peptide concentration was measured using Pierce Quantitative Colorimetric Peptide Assay Kit (cat. num. 23275, Thermo Fisher Scientific) to ensure that an equal amount of each

sample was labeled. Samples were then Tandem Mass Tag (TMT) labeled with 0.2 mg of reagent resuspended in 20 µL acetonitrile for two hours at room temperature (cat. num. 90309, Thermo Fisher Scientific TMT10plex™ Isobaric Label Reagent Set; lot no. UH285567 and 131C lot UD280157A). Labelling reactions were quenched with hydroxylamine at room temperature 15 minutes. Labelled peptides were then mixed and dried by speed vacuum.

High pH basic fractionation. The peptide mixture was resuspended in 0.1% TFA (trifluoroacetic acid) and fractionated on a Pierce™ High pH reversed-phase peptide fractionation spin column using vendor methodology (cat. num. 84868). Each fraction was dried by speed vacuum and resuspended in 30 µL 0.1% FA.

Nano-LC-MS/MS Analysis. Nano-LC-MS/MS analyses were performed on an EASY-nLC™ HPLC system coupled to an Orbitrap Fusion™ Lumos™ mass spectrometer (Thermo Fisher Scientific). Half of each fraction was loaded onto a reversed phase PepMap™ RSLC C18 column with Easy-Spray tip at 400 nL/min (ES802A, 2 µm, 100 Å, 75 µm x 25 cm). Peptides were eluted from 4-33% B over 120 minutes, 33%-80% B over 5 mins, and dropping from 50-10%B over the final 4 min (Mobile phases A: 0.1% FA, water; B: 0.1% FA, 80% Acetonitrile). Mass spectrometer settings include capillary temperature of 300 °C and ion spray voltage was kept at 1.9 kV. The mass spectrometer method was operated in positive ion mode with a 4 second cycle time data-dependent acquisition with advanced peak determination and Easy-IC on (internal calibrant). Precursor scans (m/z 375-1600) were done with an orbitrap resolution of 120000, 30% RF lens, 105 ms maximum inject time (IT), standard automatic gain control (AGC) target. MS2 filters included an intensity threshold of 2.5e-4, charges states of 2 to 6, 70%

precursor fit threshold, and 60 s dynamic exclusion with dependent scan being performed on only one charge state per precursor. Higher-energy collisional dissociation (HCD) MS2 scans were performed at 50k orbitrap resolution, fixed collision energy of 37%, 200% normalized AGC target, and dynamic maximum IT.

Data analysis. Resulting RAW files were analyzed in Proteome Discover™ 2.4 (Thermo Fisher Scientific) with FASTA databases including Swiss-Prot UniProt *Homo sapiens* sequences (downloaded 09/17/2019) plus common contaminants. SEQUEST HT searches were conducted with a maximum number of 2 missed cleavages; precursor mass tolerance of 10 ppm; and a fragment mass tolerance of 0.02 Da. Static modifications used for the search were, 1) carbamidomethylation on cysteine (C) residues; 2) TMT sixplex label on lysine (K) residues and the N-termini of peptides (for TMT quant samples only). Dynamic modifications used for the search were oxidation of M, phosphorylation on S, T, Y, and acetylation of N-termini. IP-MS Sequest results were imported into Scaffold (Proteome Software) for Fishers exact test comparison. TMT quantification methods utilized isotopic impurity levels available from Thermo Fisher. Percolator False Discovery Rate was set to a strict setting of 0.01 and a relaxed setting of 0.05. Values from both unique and razor peptides were used for quantification. In the consensus workflow, peptides were normalized by total peptide amount with no scaling. Resulting abundance values for each sample, and abundance ratio values from Proteome Discoverer™ were exported to Microsoft Excel and are available in a supplemental file.

Apoptosis assay

Apoptosis analysis of live cells was performed using Annexin-V-FLUOS Staining Kit (11858 777001, Roche, USA) according to the manual. Briefly, cells were harvested and dissociated by using 0.25% trypsin-EDTA at 37°C incubator for 10 mins. Single cells were washed in 1X PBS, then resuspend and incubated in 100 μl of Annexin-V-FLUOS labeling solution for 15 mins at room temperature, followed with the analysis on BD LSRII cytometer (Becton Dickinson, USA) or Attune NxT Flow Cytometer (Thermo Fisher Scientific, USA). Data were analyzed by using FlowJo software (Treestar, USA).

ATP detection assay

The cellular ATP level was measured by using the Luminescent ATP Detection Assay Kit (ab113849, Abcam, USA) according to the manual. Briefly, a hemocytometer was used to count the number of live cells. The same number of cells for each group was used for the ATP quantification. Live cells were resuspended in 50 μl detergent solution with 1200 rpm shaking on Eppendorf ThermoMixer C for 5 mins at room temperature. Then 50 μl substrate solution was added to the detergent solution with 1200 rpm shaking on Eppendorf ThermoMixer C for 5 mins at room temperature. All solution was transferred to Nunc™ MicroWell™ 96-Well, (Nunclon Delta-Treated, Flat-Bottom Microplate, White Polystyrene Plate, Thermo Scientific, 136101, USA), followed with luminescence detection on GloMax Discover Microplate Reader (GM3000, Promega, USA). For drug treatment assays, the final concentration of ivermectin or meclizine was 0.5 μM, and the treatment time was 3 hrs.

Intracellular calcium imaging

For confocal Ca^{2+} imaging, $[Ca^{2+}]_i$ transients were recorded in hPSC-CMs loaded with fluorescent Ca^{2+} dye fluo-4 AM, on a laser scanning confocal microscope (Olympus Fluoview 1000) equipped with a 60x oil immersion lens (NA = 1.4). Fluo-4 (loaded by 20 min incubation with 10 μM of acetoxymethylester dissolved in normal Tyrode's solution supplemented with F-127. Fluo-4 was excited with the 488-nm line of an argon laser. Fluorescence was collected through a 515- to 560-nm bandpass filter. Fluo-4 images were recorded in line-scan mode with 512 pixels per line at line rates ranging from 1.5 to 2 ms/line. To record cell-wide $[Ca^{2+}]_i$ transients, cells were excited at 0.5 Hz by field stimulation (20-40 V, 4 ms) using two parallel platinum electrodes. To determine the properties of electrically evoked changes in fluo-4 fluorescence, we first calculated the average signal intensity of each successive line in a line-scan image, to obtain the time course of the line average fluorescence, *F*(t). The *F*(t) traces were condensed into one-cycle recordings by averaging over three consecutive transients, and converted to $\Delta F/F_0(t)$, where F_0 is the average *F* over the time before the Ca^{2+} rise, and ΔF equals $F_{(t)}$-F_0. From the averaged $\Delta F/F_0(t)$ we determined the magnitudes of peak $\Delta F/F_0$, 10-90% rise time, and 30%, 50% and 90% recovery times (CaT_{30}, CaT_{50}, CaT_{90}; computed as the interval from peak ΔF/F0 to the time when ΔF/F0 is 30, 50 or 90% recovered). For Calcium irregularity analysis, hPSC-CMs were attached on 12-well plate coated with Matrigel and loaded with X-Rhod-1 (X14210, Invitrogen, USA) in DMEM medium (no glucose) with 10% FBS containing Pluronic F-127 (final concentration 0.02%, P2443, Sigma, USA) for 15 mins at 37 °C incubator. Videos were acquired at a rate of 50 frames per second using the All-in-One Fluorescence Microscope BZ-X800 (KEYENCE CORPORATION, Japan).

For drug treatment assay, the final concentration of ivermectin or meclizine was 0.5 μM (drugs were dissolved in DMSO). The calcium handling signaling was acquired after 1 hr. of drug treatment. Videos were analyzed by ImageJ software using a custom script that calculated the temporal changes in calcium fluorescence intensity.

Multi-electrode arrays (MEAs)

MEAs assay was performed with cardiomyocytes after 30 days of differentiation. All experiments were performed in DMEM medium (no glucose, Gibco, USA) with 10% FBS. Cardiomyocytes were attached on the cytoview MEA 24-well white plate (M384-tMEA-24W-5, Axion Biosystems, USA) coated with Matrigel. Electrophysiology data were acquired by using the Maestro Edge MEA System (Axion Biosystems, USA) with the integrated environment chamber controlling the heat at 37 °C and CO_2 at 5%. The Interspike interval was the time duration of two adjacent spikes (beating peaks). For the Interspike interval (ISI) distribution analysis, we calculated both of the number of spikes (beating peaks) and the ISI. Then we grouped the ISI as the time window 0-1s, 1-2s, 2-3s,3-4s, 4-5s, 5-6s, 6-7s and 7-8s and calculated the spike number frequency according to every time window to generate ISI distribution. In the Interspike interval (ISI) distribution analysis results figures. Y-axis means spikes (beating peaks) number (frequency) in a specific time window. X-axis means time (in second, s).

Immunofluorescence

For immunostaining of attached cells, cells were fixed with 4% PFA (diluted from 16% Paraformaldehyde aqueous solution) for 10 mins at room temperature. After washing with 1X PBS, cells were blocked for 1 h with 1X PBS blocking buffer containing 2% goat serum (or 5% BSA) and 0.1% saponin. Staining with corresponding primary antibodies diluted with blocking buffer was performed at 4°C for overnight. Staining with secondary antibodies were performed on the next day, following with nucleus staining with DAPI. For beating EBs immunostaining, live EBs were fixed with 4% PFA (diluted from 16% Paraformaldehyde aqueous solution) for 10 mins at room temperature, followed with 15% sucrose solution at room temperature until all EBs sunk. Then EBs were embed in OCT and cryosectioning was performed. The EBs immunostaining protocol was the same as that of attached cells. For TUNEL (Terminal deoxynucleotidyl transferase dUTP nick end labeling) experiments, staining of attached cells or EBs sections were carried out using In Situ Cell Death Detection Kit, Fluorescein (11684795910 Roche, USA) according to the manual. Leica DM6B image system was used for imaging.

Functional enrichment analysis

The GO enrichment analysis was performed by using DAVID (https://david.ncifcrf.gov/), with assistance from Gene Ontology enRIchment anaLysis and visuaLizAtion tool (http://cbl-gorilla.cs.technion.ac.il/). The canonical signaling pathway and toxicity analysis were performed by Ingenuity Pathway Analysis (QIAGEN IPA).

Supplemental References

Dobin, A., Davis, C.A., Schlesinger, F., Drenkow, J., Zaleski, C., Jha, S., Batut, P., Chaisson, M., and Gingeras, T.R. (2013). STAR: ultrafast universal RNA-seq aligner. Bioinformatics *29*, 15-21.
Levasseur, E.M., Yamada, K., Pineros, A.R., Wu, W., Syed, F., Orr, K.S., Anderson-Baucum, E., Mastracci, T.L., Maier, B., Mosley, A.L., et al. (2019). Hypusine biosynthesis in beta cells links polyamine metabolism to facultative cellular proliferation to maintain glucose homeostasis. Sci Signal *12*. eaax0715.
Liao, Y., Smyth, G.K., and Shi, W. (2019). The R package Rsubread is easier, faster, cheaper and better for alignment and quantification of RNA sequencing reads. Nucleic Acids Res *47*, e47.
Lu, T.Y., Lin, B., Li, Y., Arora, A., Han, L., Cui, C., Coronnello, C., Sheng, Y., Benos, P.V., and Yang, L. (2013). Overexpression of microRNA-1 promotes cardiomyocyte commitment from human cardiovascular progenitors via suppressing WNT and FGF signaling pathways. J Mol Cell Cardiol *63*, 146-154.
Peck Justice, S.A., Barron, M.P., Qi, G.D., Wijeratne, H.R.S., Victorino, J.F., Simpson, E.R., Vilseck, J.Z., Wijeratne, A.B., and Mosley, A.L. (2020). Mutant thermal proteome profiling for characterization of missense protein variants and their associated phenotypes within the proteome. J Biol Chem. *295*, 16219-16238.
Peltier, H.J., and Latham, G.J. (2008). Normalization of microRNA expression levels in quantitative RT-PCR assays: Identification of suitable reference RNA targets in normal and cancerous human solid tissues. Rna *14*, 844-852.
Plubell, D.L., Wilmarth, P.A., Zhao, Y., Fenton, A.M., Minnier, J., Reddy, A.P., Klimek, J., Yang, X., David, L.L., and Pamir, N. (2017). Extended Multiplexing of Tandem Mass Tags (TMT) Labeling Reveals Age and High Fat Diet Specific Proteome Changes in Mouse Epididymal Adipose Tissue. Mol Cell Proteomics *16*, 873-890.
Robinson, M.D., McCarthy, D.J., and Smyth, G.K. (2010). edgeR: a Bioconductor package for differential expression analysis of digital gene expression data. Bioinformatics *26*, 139-140.
Sharma, A., Garcia, G., Wang, Y.Z., Plummer, J.T., Morizono, K., Arumugaswami, V., and Svendsen, C.N. (2020). Human iPSC-Derived Cardiomyocytes Are Susceptible to SARS-CoV-2 Infection. Cell Rep Med *1*. 100052.

Legends of Supplementary Tables

Table S1. Whole mRNA-seq data analysis

Table S2 Global proteomics data analysis

Table S3 CoIP-MS results of hPSC-CMs

Table S4 Oligonucleotides information

REAGENT or RESOURCE	SOURCE	IDENTIFIER
**Antibodies**		
Rabbit IgG	Millipore	MAGNARIP01, RRID:AB_10681285
Mouse IgG	Millipore	MAGNARIP01, RRID:AB_2617156
Mouse IgG Isotype Control	R&D system	MAB002, RRID:AB_357344
Rabbit IgG	Cell Signaling Technology (CST)	#2729, RRID:AB_1031062
Alexa Fluor® 647 Mouse Anti-Cardiac Troponin T	BD Pharmingen	# 565744, RRID:AB_2739341
Cardiac Troponin T antibody (mouse)	Thermo Fisher	MS-295-P, RRID:AB_61806
APC goat anti-mouse IgG	BD	550826, RRID:AB_398465
Strep Tag Monoclonal Antibody (GT661)	Thermo Fisher Scientific	# MA5-17283, RRID:AB_2538749
Anti-Oct4 antibody	Cell Signaling Technology (CST)	Cat# 5177, RRID:AB_10693303
Cleaved Caspase-3 (Asp175) (5A1E) Rabbit mAb	Cell Signaling Technology (CST)	# 9664, RRID:AB_2070042
Anti-CASP3 antibody	neomarkers	RB-1197-P0, RRID:AB_60471
Anti-GAPDH Mouse mAb (6C5)	Sigma-Aldrich	CB1001, RRID:AB_2107426
Anti-cleaved CASP9 antibody (D315)	Cell Signaling Technology (CST)	9505P, RRID:AB_2290727
Anti-CASP9 antibody	Cell Signaling Technology (CST)	9502P, RRID:AB_2068621
Goat anti-Mouse IgG (H+L) Cross-Adsorbed Secondary Antibody, Alexa Fluor 647	Invitrogen	A-21235, RRID:AB_2535804
Goat anti-Rabbit IgG (H+L), Secondary Antibody, Alexa Fluor 488	Invitrogen	A27034, RRID:AB_2536097

Goat anti-Mouse IgG (H+L) Cross-Adsorbed Secondary Antibody, Cyanine5	Invitrogen	A10524, RRID:AB_2534033
**Chemicals, Peptides, and Recombinant Proteins**		
RhBMP4	R&D system	314-BP
RhFGF2	R&D system	233-FB
RActivin A	R&D system	338-AC
XAV 939	R&D system	3748
Meclizine hydrochloride	Sigma-Aldrich	1377009
Ivermectin	Sigma-Aldrich	I8898
X-Rhod-1, AM, cell permeant	Invitrogen	X14210
Pluronic® F-127	Sigma-Aldrich	P2443
DreamTaq Green PCR Master Mix (2X)	Thermo Fisher Scientific	K1081
L-(+)-Lactic acid	Sigma-Aldrich	L1750
CHIR99021	R&D system	4423
Saponin	Sigma	S4521-25G
L-Ascorbic acid 2-phosphate sesquimagnesium salt hydrate	Sigma-Aldrich	A8960-5G
Puromycin dihydrochloride	Sigma-Aldrich	P8833-10MG
Polybrene	Sigma-Aldrich	H9268-5G
TrypLE™ Express Enzyme (1X), phenol red	Gibco	12605010
DAPI Fluoromount-G	southernbiotech	0100-20
UltraPure™ 0.5M EDTA, pH 8.0	Thermo Fisher Scientific	15575020
Fast SYBR Green Master Mix	Applied Biosystems	4385612
**Critical Commercial Assays**		
B-27™ Supplement, minus insulin	Thermo Fisher Scientific	A1895601
B-27™ Supplement (50X), serum free	Thermo Fisher Scientific	17504044
4–20% Mini-PROTEAN® TGX™ Precast Protein Gels (60X)	Bio-rad	4561096
SuperSignal™ West Atto Ultimate Sensitivity Substrate	Thermo Fisher Scientific	A38555
In Situ Cell Death Detection Kit, TMR red	Roche	12156792910
Luminescent ATP Detection Assay Kit	abcam	ab113849
High-Capacity RNA-to-cDNA™ Kit	Applied Biosystems	4387406
Classic Magnetic IP/Co-IP Kit	Pierce	88804
RPMI 1640 Medium	Gibco	11875093
DMEM/F12	Gibco	11320082

DMEM, high glucose	Gibco	11965118
DMEM, no glucose	Gibco	11966025
StemPro™-34 SFM (1X)	Gibco	10639011
Pierce™ ECL Western Blotting Substrate	Pierce	32209
cOmplete™ Lysis-M EDTA-free	Roche	04719964001
RNeasy Mini Kit	Qiagen	74106
QIAquick Gel Extraction Kit Print	Qiagen	28706
QIAquick PCR Purification Kit Print	Qiagen	28106
Corning® Matrigel® Growth Factor Reduced (GFR) Basement Membrane Matrix, *LDEV-Free, 10mL	Corning	354230
**Experimental Models: Cell Lines**		
293T cells	ATCC	CRL-3216
Wildtype Human iPSC line S3	Carvajal-Vergara et al., 2010	N/A
Wildtype Human H9 hESCs	ATCC	HTB-176
Human S3 iPSCs with overexpression of SARS-CoV-2 gene Orf9c<br>Human S3 iPSCs with overexpression of EGFP	This paper	N/A
Human H9 hESCs with overexpression of SARS-CoV-2 gene Orf9c<br>Human H9 hESCs with overexpression of EGFP<br>Human H9 hESCs with overexpression of blank lentivirus backbone	This paper	N/A
**Oligonucleotides**		
Primers see Table S oligonucleotides	This paper	N/A
**Recombinant DNA**		
pLVX-EF1alpha-eGFP-2xStrep-IRES-Puro	Addgene, please see the citation [6].	Addgene number: 141395
pLXV-EF1alpha-2xStrep-SARS-CoV-2-orf9c-IRES-Puro	Addgene, please see the citation [6].	Addgene number: 141393
psPAX2	From Dr. Gang Hu lab (NIH)	N/A
pMD2.G	From Dr. Gang Hu lab (NIH)	N/A
**Software and Algorithms**		
Image J	National Institutes of Health	https://imagej.nih.gov/ij/

Ingenuity Pathway Analysis (IPA)	QIAGEN	https://www.qiagen.com/us/products/discovery-and-translational-research/next-generation-sequencing/informatics-and-data/interpretation-content-databases/ingenuity-pathway-analysis/?clear=true#orderinginformation
GENE ONTOLOGY	THE GENE ONTOLOGY RESOURCE	http://geneontology.org/
STRING	STRING CONSORTIUM	https://string-db.org
Adobe Illustrator	Adobe Inc.	https://www.adobe.com/products/illustrator.html
FlowJo (Treestar)	FlowJo, LLC	https://www.flowjo.com/about/company
**Bacterial and viral strains**		
MAX Efficiency™ DH5α Competent Cells	Invitrogen	18258012
One Shot™ Stbl3™ Chemically Competent E. coli	Invitrogen	C737303

Supplementary Figure 1

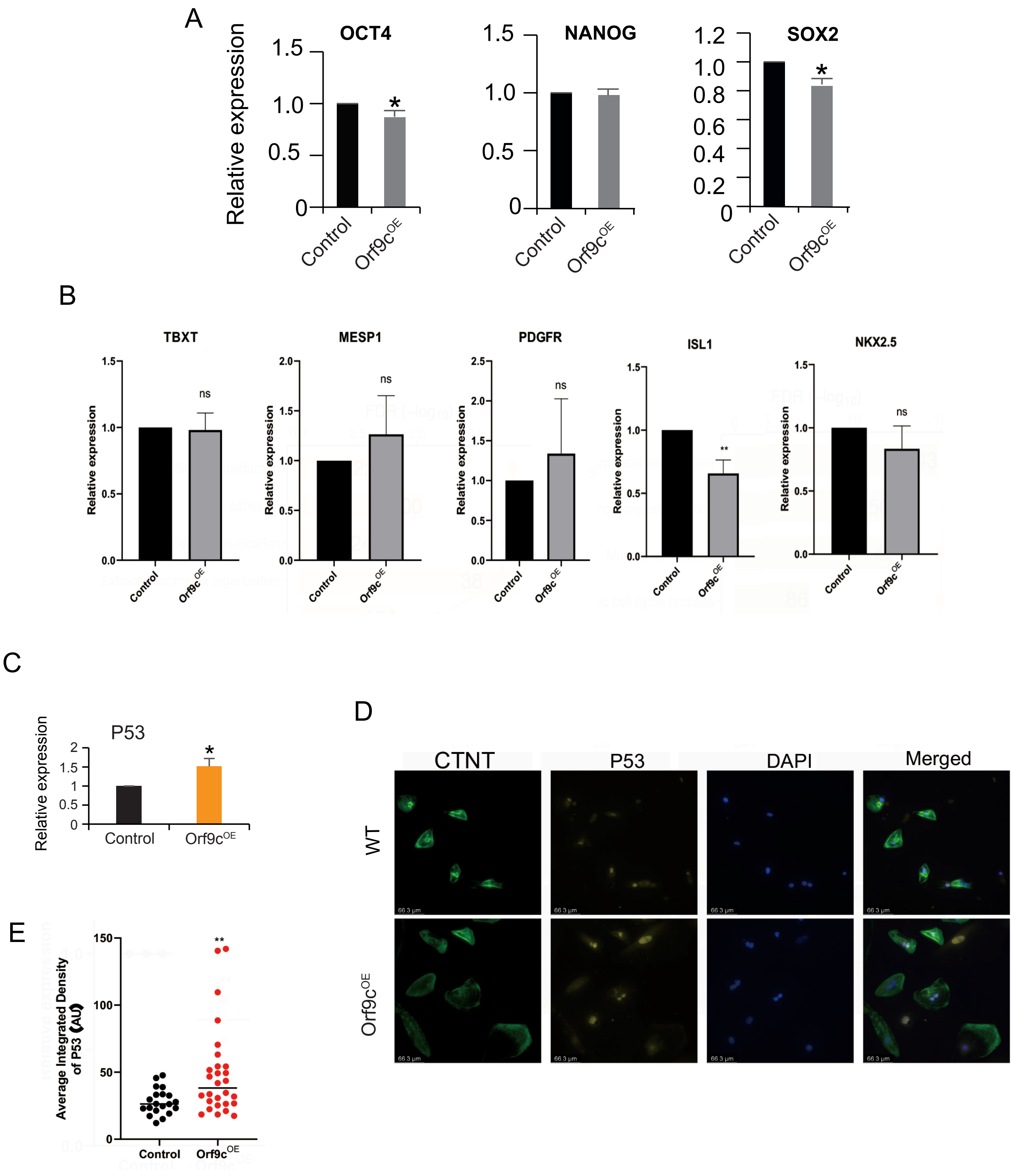

Supplementary Figure 1. Effect of Orf9c on gene expressions of hPSC-CMs.
(A) RT-qPCR analysis of the expression levels of pluripotency marker genes. n=3. t-test. *$p < 0.05$.
(B) RT-qPCR analysis of the expression levels of cardiac marker genes. n=3. t-test. **$p < 0.01$.
(C) RT-qPCR analysis of the P53 in control and Orf9cOE hPSC-CMs. n=3. t-test. *$p < 0.05$.
(D) Representative immunostaining of P53 in in control and Orf9cOE hPSC-CMs, with statistical analysis of fluorescence desnsity of P53 in CMs of (E). t-test. **$p < 0.01$.

Supplementary Figure 2

A GO_upregulated proteins (Orf9c^{OE} vs. Control)

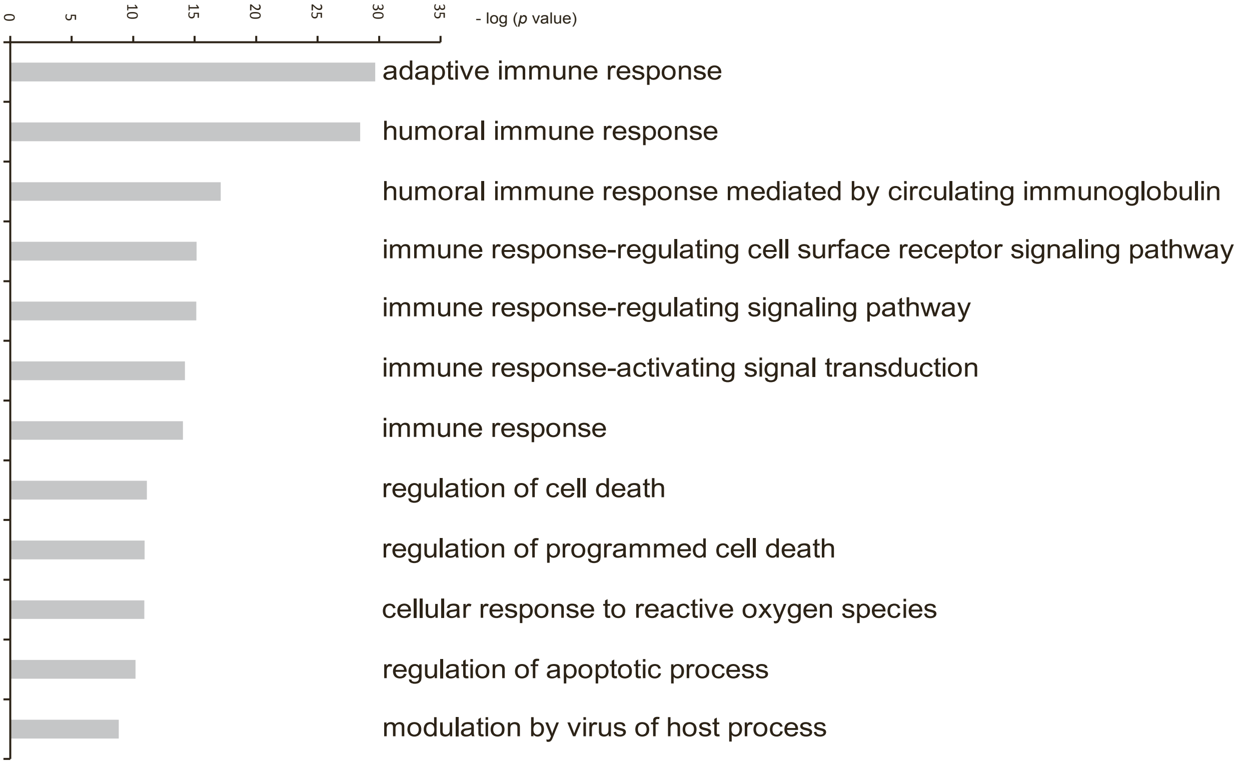

B Upregulated proteins (Orf9c^{OE} vs. Control)
Immune response

ATF2 CAMK4 GNA13 GNB1L GNG4 HLA-A HLA-B ITPR2 JUN MAP2K1 NFATC4 NFKB2 PIK3CD PLCG1 PRKCQ RALA RAP2B RELA XPO1

C Upregulated proteins (Orf9c^{OE} vs. Control)
Inflammation response

ATF2 BIRC5 CALB2 CASP8 CREB1 HLA-A HLA-B HMGB1 HMOX1 IRAK1 JUN MAPK7 MAPT NFATC4 NFKB2 PIK3CD PLCG1 RELA RIPK1 WNT1

D Down-regulated proteins (Orf9c^{OE} vs. Control)
Mitochondrial ATP biosynthesis

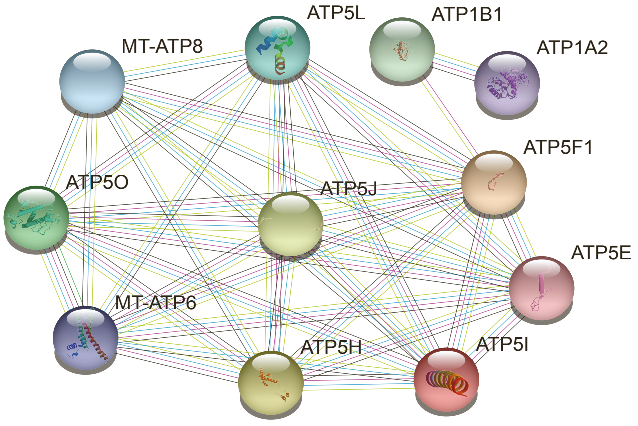

E Down-regulated proteins (Orf9c^{OE} vs. Control)
Respiratory electron transport chain

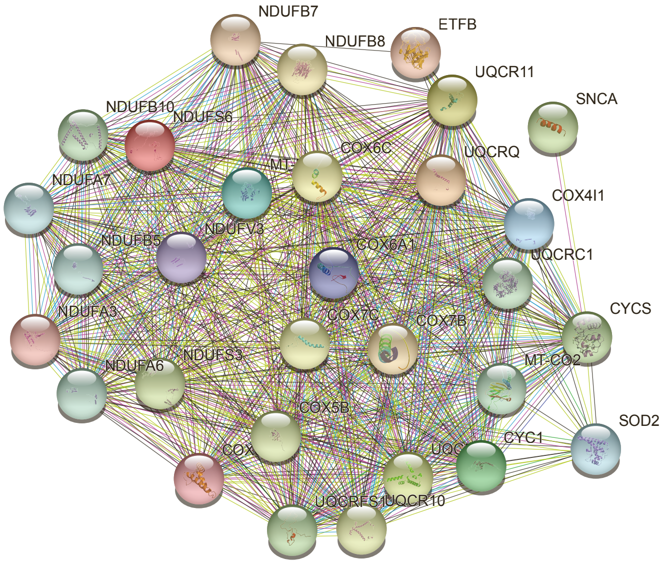

Supplementary Figure 2. Genome-wide proteomics and protein-protein interaction analysis reveals proteins with differential expression levels induced by SARS-CoV-2 gene Orf9c.
(A) Gene Ontology (GO) analysis of upregulated proteins (Orf9cOE vs. Control) in hiPSC-CMs.
(B) Upregulated proteins involved in immune response by Orf9c overexpression in hiPSC-CMs.
(C) Upregulated proteins involved in inflammation response induced by Orf9c overexpression in hiPSC-CMs.
(D) Protein-protein interaction analysis showing down-regulated proteins, induced by Orf9c overexpression in hiPSC-CMs, associate with mitochondrial ATP biosynthesis.
(E) Protein-protein interaction analysis showing down-regulated proteins, induced by Orf9c in hiPSC-CMs, associate with respiratory electron transport chain.

Supplementary Figure 3

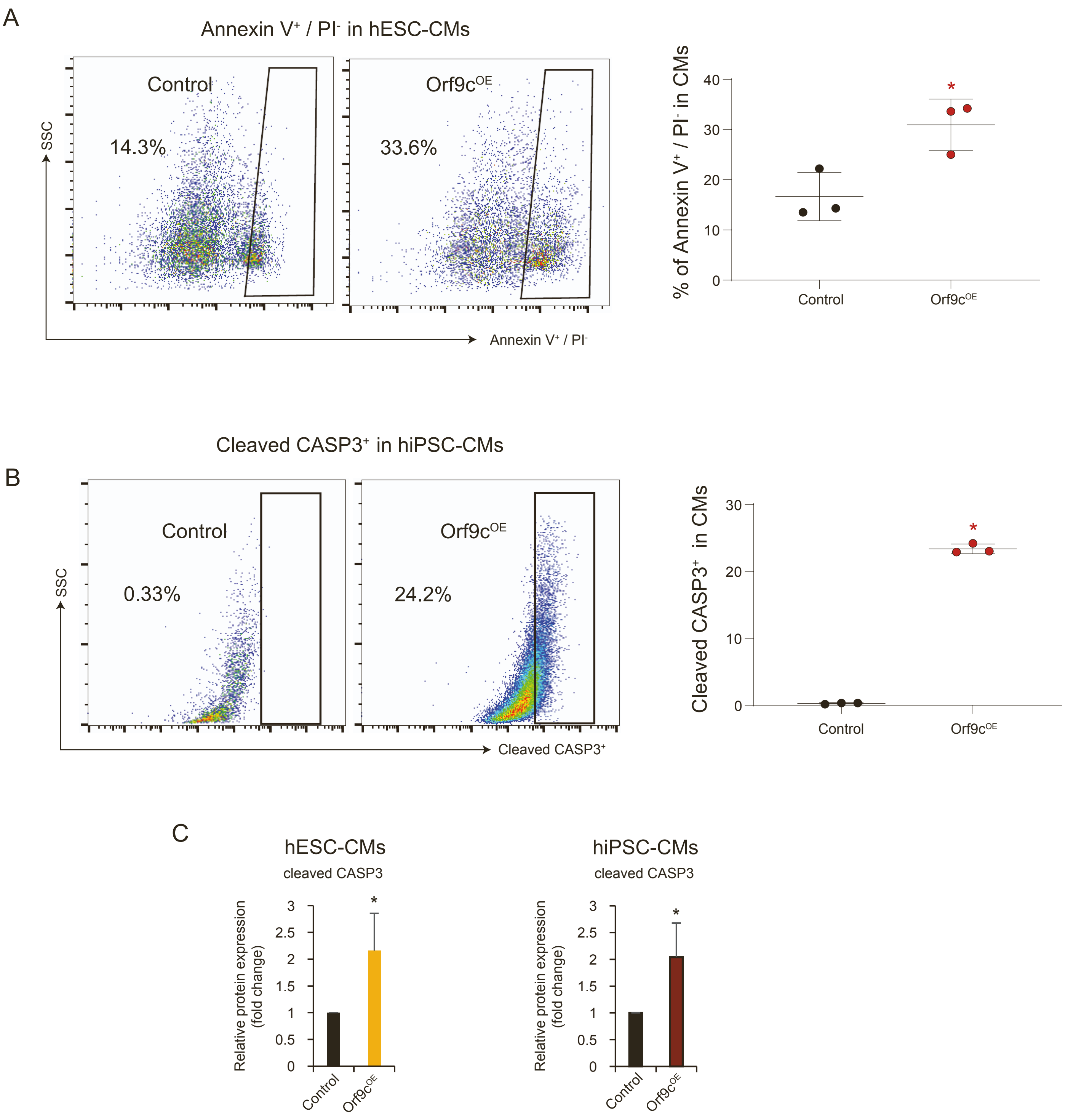

Supplementary Figure 3. Orf9c induces cell death in hPSC-CMs.

(A) Flow cytometry analysis of Annexin V+ / PI- CMs in CM population of hESC-CMs. All bars are shown as mean ± SD. (n=3). A two-tailed unpaired t-test was used to calculate P-values: *$p < 0.05$ (vs. Control).

(B) Flow cytometry analysis of Cleaved-CASP3+ CMs in CM population of hiPSC-CMs. All bars are shown as mean ± SD. (n=3). A two-tailed unpaired t-test was used to calculate P-values: *$p < 0.05$ (vs. Control).

(C) Statistical results of the cleaved CASP3 protein levels in control and Orf9c-OE hPSC-CMs. A two-tailed unpaired t-test was used to calculate P-values: *$p < 0.05$ (vs. Control).n=3.

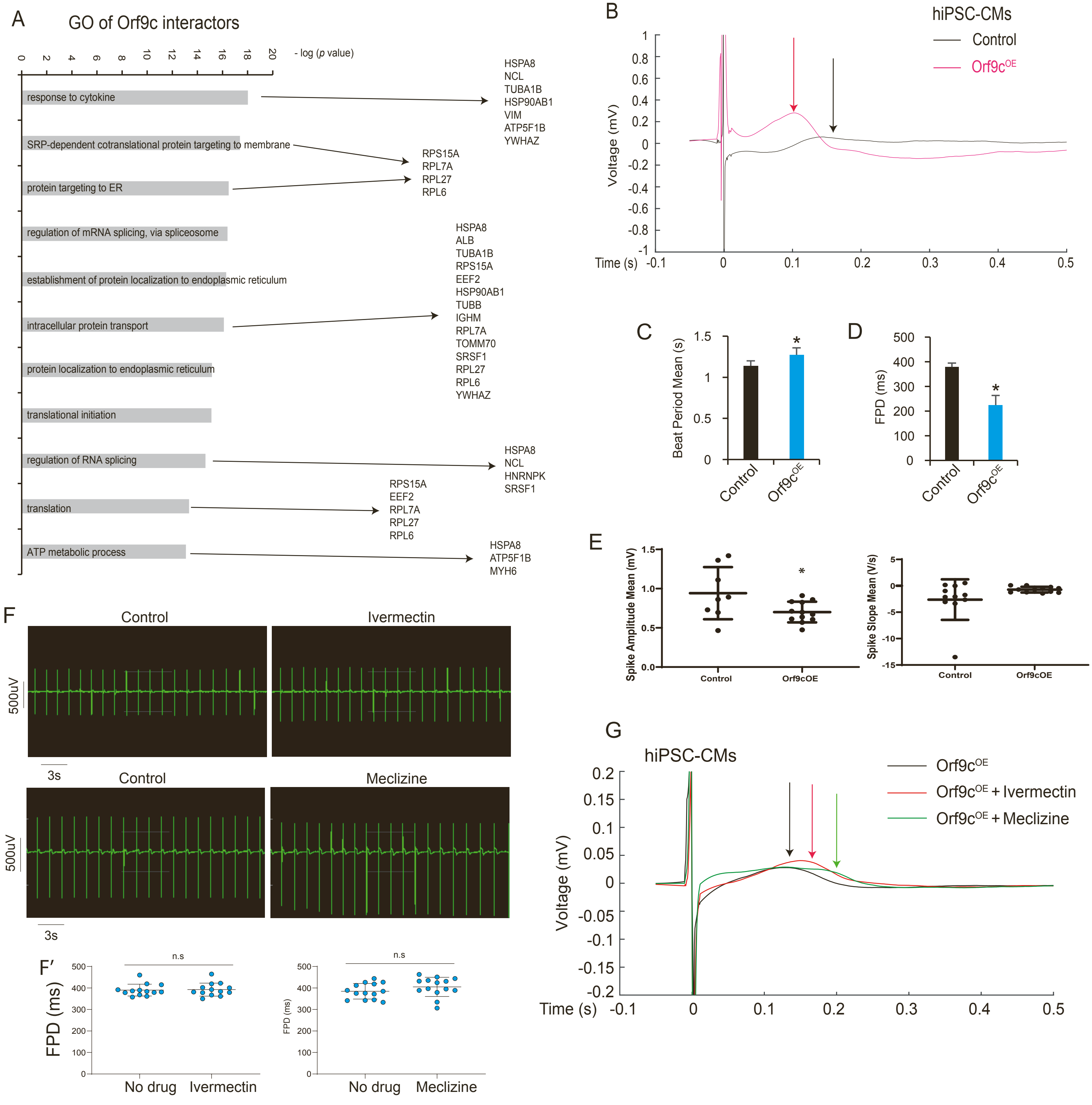

Supplementary Figure 4. The function of Orf9c in hPSC-CMs and FDA-approved drugs elongated FPDs of Orf9cOE hPSC-CMs.

(A) Gene Ontology (GO) analysis of Orf9c interactors in hESC-CMs.

(B) Representative traces illustrate the change of field potential duration (FPD, ms) in Orf9c-OE vs. control hiPSC-CMs.

(C) Beat Period Mean of MEA data in hESC-CMs. Bars are shown as mean ± SD. (n = 4). A two-tailed unpaired t-test was used to calculate P-values. *p < 0.05.

(D) Field potential duration (FPD) analysis of MEA data in hESC-CMs. *p < 0.05. Same statistical method as (C).

(E) Mean spike amplitude and slope of Orf9cOE vs. control hESC-CMs. **p* < 0.05. t-test.

(F) Representative MEA recordings of spontaneous electrical activities of control hESC-CMs without and with drug treatment, with statistical results of FPD in F′. Final concentration of ivermectin and meclizine was 0.5 μM. Treatment time was 2 hrs. n.s., no significance. Same statistical method as (C).

(G) Representative traces illustrate the change of field potential duration (FPD, ms) in Orf9cOE hiPSC-CMs with vs. without drug treatments. Ivermectin and Meclizine final concentrations were 0.5 μM. Treatment time was 2 hrs.
